# Asymmetric by Design: Heteroleptic Coordination Compounds with Redox-Active Dithiolene and 1,2,4,5-Tetrakis(isopropylthio)benzene Ligands

**DOI:** 10.1021/acs.inorgchem.3c02928

**Published:** 2023-12-22

**Authors:** Che Wu, Lakshmi Nishanth Kakarla, Chandru P. Chandrasekaran, Xiaodong Zhang, Joel T. Mague, Stephen Sproules, James P. Donahue

**Affiliations:** † Department of Chemistry, Tulane University, 6400 Freret Street, New Orleans, Louisiana 70118-5698, United States; ‡ Department of Chemistry and Biochemistry, Lamar University, Beaumont, Texas 77710, United States; § WestCHEM, School of Chemistry, University of Glasgow, Glasgow G12 8QQ, United Kingdom

## Abstract

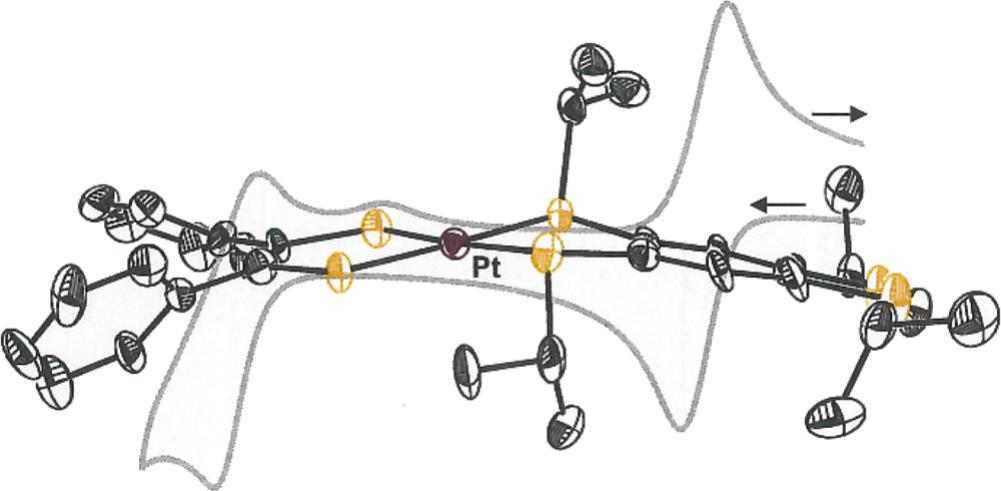

The 1,2,4,5-tetrakis(alkylthio)benzenes are redox-active organosulfur molecules that support oxidation to a stable radical cation. Their utility as ligands for the assembly of multimetal complexes with tailored functionality/property is unexamined. Here, 1,2,4,5-tetrakis(isopropylthio)benzene (tptbz, **1**) is shown to bind PdCl_2_ at either one end, leaving the other open, or at both ends to form centrosymmetric [Cl_2_Pd(tptbz)PdCl_2_], **4**. Ligand metathesis between Na_2_[(N≡C)_2_C_2_S_2_] (Na_2_mnt) and [Cl_2_M(tptbz)] (M = Pd, **2**; M = Pt, **3**) yields [(mnt)M(tptbz)] (M = Pd, **5**; M = Pt, **6**), but an alternative route involving transmetalation with [(mnt)SnMe_2_] delivers substantially greater yield. The mixed dithiolene-dithioether compound [(Ph_2_C_2_S_2_)Pt(tptbz)] (**8**) is formed by a similar transmetalation protocol using [(Ph_2_C_2_S_2_)Sn^*n*^Bu_2_]. Compounds **5**, **6**, and **8** are the first such heteroleptic complexes prepared by deliberate synthesis. The cyclic voltammetry of **8** reveals anodic waves at +0.14 and +0.97 V vs Fc^+^/Fc, which are attributed to successive dithiolene oxidation processes. While oxidized at +0.73 V as a free ligand, the redox-active MO of tptbz is pushed to a higher potential upon coordination to Pt^2+^ and is inaccessible. Calculations of the structures of [**8**]^+^ and of [((Cl_2_-3,5-C_6_H_3_)_2_C_2_S_2_)Pt(tptbz)]^+^ show that, in the latter, the dithiolene MOs are drawn down in energy into proximity with the tptbz MOs.

## Abstract 2

Tetrakis-1,2,4,5-isopropylthiobenzene (tptbz), a redox-active molecule with oxidation at +0.73 V vs Fc^+^/Fc in CH_2_Cl_2_, binds MCl_2_ (M = Pd, Pt) to afford [Cl_2_M(tptbz)] or dinuclear [Cl_2_Pd(tptbz)PdCl_2_]. Treatment of [Cl_2_M(tptbz)] with [((N≡C)_2_C_2_S_2_)SnMe_2_] or [(Ph_2_C_2_S_2_)Sn^*n*^Bu_2_] produces heteroleptic dithiolene-dithioether compounds [((N≡C)_2_C_2_S_2_)M(tptbz)] and [(Ph_2_C_2_S_2_)Sn(tptbz)]. Two oxidations are observed for [(Ph_2_C_2_S_2_)Sn(tptbz)], both attributable to the [Ph_2_C_2_S_2_]^2−^ ligand. Calculations suggest that electron-withdrawing substituents on [Ph_2_C_2_S_2_]^2−^ draw the tptbz-based MOs close enough to the HOMO to be competitive as locus for oxidation.

## Introduction

**Figure 1:**
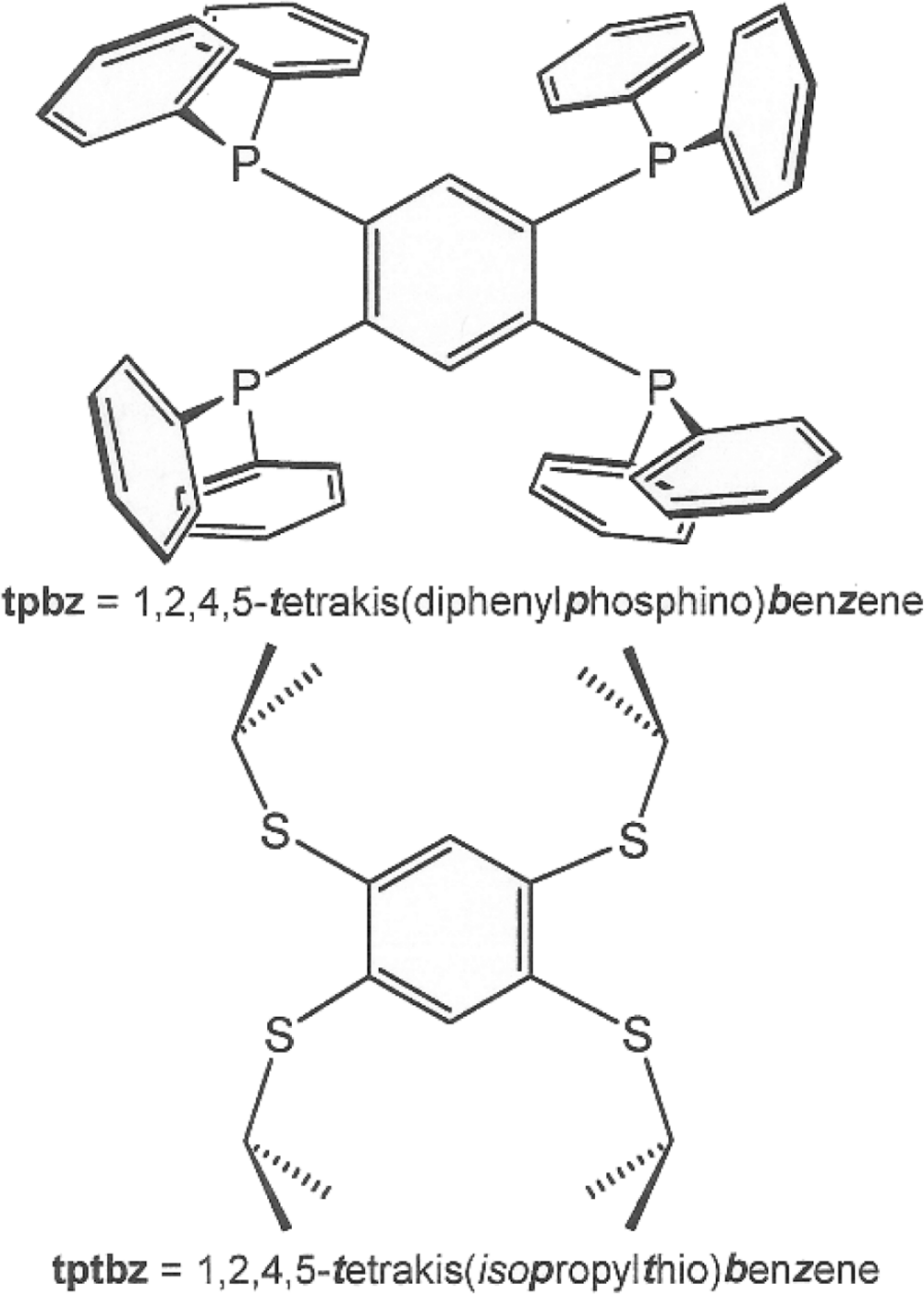
Structures of the tpbz and tptbz ligands.

**Scheme 1:**
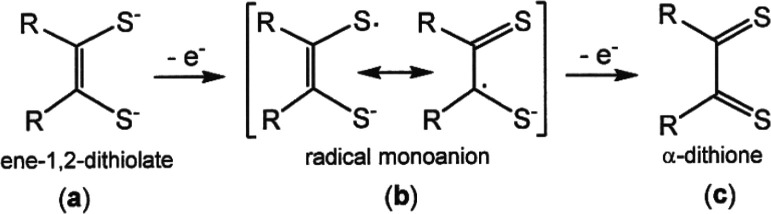
Redox Levels of the Dithiolene Ligand: (a) Fully Reduced Ene-1,2-dithiolate, (b) Half-Oxidized Radical Monoanion, and (c) Fully Oxidized α-Dithione This figure is a modification of a scheme previously published.^2^

In a series of recent reports,^1−4^ we have described the utility of 1,2,4,5-tetrakis(diphenylphosphino)benzene (tpbz; Figure 1) as a rigid connector between redox-active metallodithiolene groups, which can be reversibly oxidized to access the radical monoanionic form of the dithiolene ligand ((**b**) in Scheme 1). The tpbz ligand is an effective electronic insulator that permits only weak dipolar coupling between the peripheral ligand radicals, thereby enabling the creation of a coherent quantum state that in principle could function as a qubit in quantum computing or memory applications. Improvement of coherence lifetimes in qubit candidates based upon electron-spins can be accomplished by minimizing the presence of spin-active nuclei in the vicinity,^5^ the effect of which is to induce decoherence in the entangled state. In this regard, ^31^P with *I* = 1/2 at 100% natural abundance is a less desirable nuclide for incorporation into a molecule-based qubit than is ^32^S, with *I* = 0 at 95% natural abundance, because its nuclear spin contributes to the decohering magnetic background “noise”.

Quite apart from the removal of spin-active nuclei that undercut coherent quantum state lifetimes, substitution of the tpbz connector for a tetrathioarene bridge creates altogether new possibilities for qubit engineering in that it, just as the dithiolene ligand opposite it in a heteroleptic complex, can sustain reversible oxidation to a spin-delocalized radical. Indeed, the cations that are accessible from 1,2,4,5-tetrathioarenes have been the subject of spectroscopic study, first by Pedulli and co-workers,^6^ and later by Chen and associates,^7^ who also reported solid-state conductivity measurements and the first instances of structural characterization of such radical cationic salts by X-ray crystallography. Thus, if amenable to the formation of heteroleptic complexes with dithiolene ligands, tetrathioarenes might enable the formation of multiqubit systems whose component spins reside in chemically distinctive environments and thus might be separately addressable.

The foregoing considerations motivated us to examine the feasibility of substituting the tpbz connector with the 1,2,4,5-tetrakis(isopropylthioether)benzene (tptbz) platform. Like tpbz, this tetrathioether has the virtues of amenability to scaled synthesis and stability in air, but it enjoys the further advantage that the alkyl groups of the thioether groups can be readily varied for effect upon solubility and steric profile. However, thioethers are generally weak ligands with a decided binding preference for soft, later transition metals of the second and third rows. Nonetheless, the potential advantages offered by tptbz in contrast to tpbz in supporting ligand-based coherent quantum states are sufficient to justify efforts to synthesize mono- and dimetallic compounds of the forms [(R_2_C_2_S_2_)M(tptbz)] and [(R_2_C_2_S_2_)M(μ-tptbz)M(S_2_C_2_R_2_)]. In this report, we present the leading results of this exploratory synthetic foray.

## Experimental Section

### Physical Methods

All ^1^H spectra were recorded at 25 °C with a Bruker Avance spectrometer operating at 300.13 MHz and referenced to the protonated solvent residual. Mass spectra (ESI^+^) were obtained with a Bruker micrOTOF II mass spectrometer with an Agilent Technologies 1200 Series LC. The UV–vis spectra were acquired on a Hewlett-Packard 8752A diode array spectrometer. Elemental analyses were performed by Galbraith Laboratories, Inc. of Nashville, TN, or by Kolbe Microanalytical Laboratory in Oberhausen, Germany. Electrochemical measurements were performed using a CHI 620C electrochemical analyzer workstation with a Ag/AgCl reference electrode, glassy carbon working electrode, Pt wire as the auxiliary electrode, and [^*n*^Bu_4_N][PF_6_] as the supporting electrolyte. Under these conditions, the Cp_2_Fe^+^/Cp_2_Fe couple consistently occurred at +0.50 V. Spectroelectrochemical measurements were made with an Ocean Optics HR2000 spectrophotometer along with a Pine Research Instruments platinum honeycomb working electrode and a Ag/AgCl reference electrode. Procedural details regarding crystal growth, X-ray diffraction data collection, data processing, and structure solution and refinement are deferred to the Supporting Information (SI). Unit cell data and selected refinement statistics for the compounds that have been structurally identified are presented in Table 1; more complete crystallographic data are summarized in Table S1.

**Table 1:** Unit Cell Refinement Data for Crystallographically Characterized Compounds

	tptbz	C_6_(S^*i*^Pr)_6_	[(pdt)Sn^*n*^Bu_2_]	[Cl_2_Pd(tptbz)]	[Cl_2_Pt(tptbz)]
formula	C_18_H_30_S_4_	C_24_H_42_S_6_	C_22_H_28_S_2_Sn	C_18_H_30_Cl_2_PdS_4_	C_18_H_30_Cl_2_PtS_4_
fw, g/mol	374.66	522.93	475.25	551.96	640.65
xtl system	monoclinic	triclinic	tetragonal	monoclinic	monoclinic
space grp	*P*2_1_/*c*	*P*1̅	*I*4̅	*P*2_1_/*c*	*P*2_1_/*c*
*a*, Å	10.1189(12)	9.1370(2)	21.0089(3)	13.8803(12)	13.8706(8)
*b*, Å	8.3483(10)	9.6108(2)	21.0089(3)	9.7851(9)	9.6553(6)
*c*, Å	12.5378(15)	9.9046(2)	10.0948(2)	18.3062(16)	18.5310(11)
α, deg	90	72.062(1)	90	90	90
β, deg	98.834(2)	71.949(1)	90	98.921(3)	100.312(3)
γ, deg	90	63.875(1)	90	90	90
*V*, Å^3^	1046.6(2)	727.32(3)	4455.58(15)	2456.3(4)	2441.7(3)
*Z*	2	1	8	4	4
xtl size, mm	0.10 × 0.13 × 0.27	0.09 × 0.19 × 0.29	0.16 × 0.18 × 0.23	0.06 × 0.13 × 0.20	0.09 × 0.20 × 0.25
color, habit	colorless column	yellow plate	colorless block	yellow slat	yellow plate
indices, *h*	–13 ≤ *h* ≤ 13	–11 ≤ *h* ≤ 11	–32 ≤ *h* ≤ 32	–17 ≤ *h* ≤ 17	–18 ≤ *h* ≤ 18
indices, *k*	–11 ≤ *k* ≤ 10	–12 ≤ *k* ≤ 12	–32 ≤ *k* ≤ 32	–12 ≤ *k* ≤ 12	–13 ≤ *k* ≤ 13
indices, *l*	–16 ≤ *l* ≤ 16	–12 ≤ *l* ≤ 12	–15 ≤ *l* ≤ 15	–23 ≤ *l* ≤ 23	–25 ≤ *l* ≤ 25
2θ range, deg	2.074–57.586	4.818–53.106	4.476–66.450	4.374–54.526	4.774–58.666
refl. coll.^a^	19285	25064	128770	105892	116883
indp. refl.	19285	3018	7176	5476	6591
GooF^b^	1.038	1.038	1.045	1.124	1,018
R1,^c^^,^^d^ wR2^e^^,^^d^	0.0446, 0.1185	0.0569, 0.1591	0.0236, 0.0649	0.0822, 0.2262	0.0453, 0.1137
R1,^c^^,^^f^ wR2^e^^,^^f^	0.0598, 0.1270	0.0643, 0.1692	0.0250, 0.0653	0.1006, 0.2387	0.0618, 0.1269

	{[Cu(tptbz)][PF_6_]}_*n*_	[Cl_2_Pd(tptbz)PdCl_2_]	[(mnt)Pd(tptbz)]	[(mnt)Pt(tptbz)]	[(pdt)Pt(tptbz)]
solvent	DMF	2DMI	–	–	–
formula	C_21_H_33_CuF_6_NOPS_4_	C_28_H_50_Cl_4_N_4_O_2_Pd_2_S_4_	C_22_H_30_N_2_PdS_6_	C_22_H_30_N_2_PtS_6_	C_32_H_40_PtS_6_
fw, g/mol	652.23	957.56	621.24	709.93	812.09
xtl system	monoclinic	triclinic	monoclinic	monoclinic	triclinic
space grp	*C*2/*m*	*P*1̅	*P*2_1_/*c*	*P*2_1_/*c*	*P*1̅
*a*, Å	19.133(3)	7.2811(9)	5.7962(1)	5.7765(1)	15.9373(8)
*b*, Å	17.253(2)	10.6186(14)	19.2210(5)	19.2629(4)	16.3234(8)
*c*, Å	18.461(3)	12.9352(18)	24.1842(6)	24.2681(5)	22.6186(12)
α, deg	90	75.021(6)	90	90	85.242(2)
β, deg	107.016(4)	80.911(6)	94.386(1)	94.555(1)	78.027(2)
γ, deg	90	88.730(5)	90	90	62.321(2)
*V*, Å^3^	5827.3(14)	953.82	2686.44(11)	2691.83(9)	5096.9(5)
*Z*	8	1	4	4	6
2θ range, deg	2.306–66.588	5.784–52.984	4.562–63.908	2.276–41.251	1.84–61.276
xtl size, mm	0.08 × 0.21 × 0.23	0.10 × 0.18 × 0.41	0.06 × 0.26 × 0.37	0.06 × 0.07 × 0.31	0.07 × 0.18 × 0.30
color, habit	colorless tablet	yellow bar	green plate	yellow column	yellow plate
indices, *h*	–29 ≤ *h* ≤ 29	–8 ≤ *h* ≤ 9	–8 ≤ *h* ≤ 8	–10 ≤ *h* ≤ 10	–22 ≤ *h* ≤ 22
indices, *k*	–26 ≤ *k* ≤ 26	–12 ≤ *k* ≤ 13	–28 ≤ *k* ≤ 28	–35 ≤ *k* ≤ 35	–23 ≤ *k* ≤ 23
indices, *l*	–28 ≤ *l* ≤ 28	0 ≤ *l* ≤ 16	–35 ≤ *l* ≤ 35	–44 ≤ *l* ≤ 45	0 ≤ *l* ≤ 32
refl. coll.^a^	151087	3351	177124	213713	48672
indp. refl.	11481	3351	9273	18001	48672
GooF^b^	1.035	1.141	1.175	1.099	1.095
R1,^c^^,^^d^ wR2^e^^,^^d^	0.0423, 0.1193	0.0876, 0.2270	0.0255, 0.0536	0.0433, 0.0874	0.0381, 0.0791
R1,^c^^,^^f^ wR2^e^^,^^f^	0.0588, 0.1336	0.1110, 0.2386	0.0282, 0.0547	0.0578, 0.0925	0.0602, 0.0892

a refl. coll. = total reflections collected.

b GooF = {∑[*w*(*F*_o_^2^ – *F*_c_^2^)^2^]/(*n* – *p*)}^1/2^, where *n* is the number of reflections and *p* is the total number of parameters refined.

c R1 = ∑||*F*_o_| – |*F*_c_||/∑|*F*_o_|.

d R indices for data cut off at *I* > 2σ(*I*).

e wR2 = {∑[*w*(*F*_o_^2^ – *F*_c_^2^)^2^]/∑[*w*(*F*_o_^2^)^2^]}^1/2^; *w* = 1/[σ^2^(*F*_o_^2^) + (*xP*)^2^ + *yP*], where *P* = [2*F*_c_^2^ + max(*F*_o_^2^, 0)]/3.

f R indices for all data.

### General Considerations

Literature methods were implemented for the syntheses of the tptbz ligand, **1**,^8^ disodium maleonitriledithiolate(2−),^9^ [(mnt)SnMe_2_],^10^ and 4,5-diphenyl-1,3-dithiol-2-one.^11^ All other reagents were purchased from commercial sources and used as received. Solvents were either dried with a system of drying columns from the Glass Contour Company (CH_2_Cl_2_, Et_2_O) or freshly distilled according to standard procedures (MeOH, CH_3_CN).^12^ All reactions described below were conducted under an atmosphere of N_2_, where mnt = *m*aleo*n*itriledi*t*hiolate(2−) = [(NC)_2_C_2_S_2_]^2–^; pdt = [Ph_2_C_2_S_2_]^2–^ = 1,2-diphenylethylene-1,2-dithiolate(2−).

### Syntheses

#### C_6_(S^*i*^Pr)_6_

The following procedure is a modification of a published preparation of C_6_(S^*i*^Pr)_6._^12^ Sodium isopropyl thiolate (1.341 g, 13.66 mmol) was dissolved in 10 mL of dry DMF, and the solution was cooled in an ice bath to 0 °C. To this mixture, C_6_F_6_ (0.16 mL, 1.39 mmol) was added dropwise, and the mixture was allowed to warm to room temperature overnight, whereupon it was diluted with H_2_O (50 mL). The mixture was then extracted with Et_2_O (3 × 50 mL), and the combined organic extracts were washed with 2 × 50 mL of H_2_O and dried over anhydrous MgSO_4_. Hexakis(isopropylthio)benzene was obtained as a yellow crystalline solid after the removal of the solvent and trituration with 30 mL of cold MeOH. Yield: 0.4175 g, 58%. ^1^H NMR (δ, CDCl_3_): 3.75 (sept, 6 H, *J*_HH_ = 6.7 Hz, −C*H*(CH_3_)_2_), 1.16 (d, 36 H, *J*_HH_ = 6.7 Hz, −CH(C*H*_3_)_2_). ^13^C NMR (δ, CDCl_3_): 146.1, 41.0, 22.9.

#### [Cl_2_Pd(tptbz)], **2**

To a 25 mL Schlenk flask charged with a stirring bar and PdCl_2_ (0.248 g, 1.41 mmol), 20 mL of MeCN were added under an active N_2_ flow. The mixture was heated to ∼70 °C until most of the solids dissolved. To this mixture was then added solid tptbz (0.528 g, 1.40 mmol), and the mixture was left to stir at ambient temperature overnight. All volatiles were removed from the reaction mixture under reduced pressure, and the orange solid residue was washed with Et_2_O (2 × 20 mL) and collected by filtration. Yield: 0.651 g, 1.18 mmol, 85%. ^1^H NMR (δ, CDCl_3_): 7.38 (s, 2 H, aromatic C–*H*), 3.98 (sept, 2 H, *J*_HH_ = 6.8 Hz, PdSC*H*(CH_3_)_2_), 3.55 (sept, 2 H, *J*_HH_ = 6.6 Hz, SC*H*(CH_3_)_2_)), 1.39 (d, 24 H, *J*_HH_ = 6.5 Hz, −CH(C*H*_3_)_2_). Anal. Calcd for C_18_H_30_S_4_Cl_2_Pd: C, 39.16; H, 5.48; S, 23.24. Found: C, 39.17; H, 5.14; S, 20.84.

#### [Cl_2_Pt(tptbz)], **3**

A 50 mL Schlenk flask with PtCl_2_ (0.241 g, 0.906 mmol) was charged with MeCN (15 mL) via a syringe, and the mixture was heated at ∼70 °C until most of the solids dissolved and a yellow solution was formed. To this mixture was added solid tptbz (0.345 g, 0.921 mmol) under an outward flow of N_2_, and the mixture was left to stir at ambient temperature overnight. All volatiles were then removed *in vacuo*. The solid residue was redissolved in 2 mL of CH_2_Cl_2_ and then precipitated by the addition of 50 mL of Et_2_O. The resulting pale yellow solid was isolated by cannula filtration, washed with an additional portion of Et_2_O (25 mL), and then dried under vacuum. Yield: 0.348 g, 60% yield. ^1^H NMR (δ, CDCl_3_): 7.47 (s, 2 H, aromatic C–*H*), 3.98–3.81 (m, 2 H, PtSC*H*(CH_3_)_2_), 3.63–3.51 (m, 2 H, SC*H*(CH_3_)_2_), 1.48–1.29 (m, 24 H, SCH(C*H*_3_)_2_). Anal. Calcd for C_18_H_30_S_4_Cl_2_Pt: C, 33.74; H, 4.72. Found: C, 33.71; H, 4.77.

#### [Cl_2_Pd(tptbz)PdCl_2_], **4**

##### Method A

To a 50 mL Schlenk flask charged with PdCl_2_ (0.040 g, 0.22 mmol) was added MeCN (15 mL) via a syringe, and the mixture was heated at ∼70 °C until most solids dissolved. To this mixture was added a solution of [(tptbz)PdCl_2_] (0.124 g, 0.225 mmol) in CH_2_Cl_2_ (15 mL) via a cannula, which induced the immediate formation of a yellow precipitate. Stirring was continued at ambient temperature for 12 h. All volatile materials were removed *in vacuo*, and the remaining solids were collected as the product (0.165 g, 100%). ^1^H NMR (δ, DMSO-*d*_6_): 7.79 (s, 2 H, aromatic C–*H*), 3.83 (septet, 4 H, *J*_H–H_ = 6.5 Hz, SC*H*(CH_3_)_2_), 1.46 (s, br, 12 H, SCH(C*H*_3_)_2_), 1.30 (d, 12 H, *J*_H–H_ = 6.5 Hz, SCH(C*H*_3_)_2_). Calcd for [C_18_H_28_S_4_Cl_4_Pd_2_ + Na^+^]^+^: *m*/*z* 752.7897; Observed: *m*/*z* 752.7932; Error (δ) 4.72 ppm.

##### Method B

In a 50 mL Schlenk flask charged with PdCl_2_ (0.112 g, 0.632 mmol), MeCN (10 mL) was added under an active N_2_ flow. The mixture was heated to ∼70 °C until most of the solids dissolved, and the solvent was then removed under reduced pressure. Dichloromethane (20 mL) and solid tptbz (0.120 g, 0.320 mmol) were added, and the mixture was stirred at ambient temperature overnight. The solvent was again removed under vacuum, and the yellow solid residue was collected as the product.

#### [(mnt)Pd(tptbz)], **5**

##### Method A

In a 25 mL Schlenk tube, Na_2_[mnt] (0.042 g, 0.23 mmol) was combined with dry CH_2_Cl_2_ (10 mL) under an atmosphere of N_2_. To this mixture, solid [Cl_2_Pd(tptbz)] (0.104 g, 0.188 mmol) was added along with an additional portion of dry CH_2_Cl_2_ (5 mL). The mixture was then stirred at ambient temperature overnight, at which point it was then filtered. The filtrate was reduced to a volume of ∼0.5 mL before being combined with dry MeOH (∼15 mL). The resulting solid precipitate was collected and recrystallized as green plates from dry CH_2_Cl_2_ (2 mL) by slow introduction of dry MeOH (50 mL). Yield: 0.0329 g, 0.0530 mmol, 28.1%. ^1^H NMR (δ, CDCl_3_): 7.49 (s, 2 H, aromatic C–*H*), 3.56 (sept, 2 H, *J*_H–H_ = 6.6 Hz, PdSC*H*(CH_3_)_2_), 3.47, (sept, 2 H, *J*_H–H_ = 6.6 Hz, SC*H*(CH_3_)_2_), 1.45 (d, 12 H, *J*_HH_ = 6.7 Hz, PdSCH(C*H*_3_)_2_), 1.41 (d, 12 H, *J*_HH_ = 6.7 Hz, SCH(C*H*_3_)_2_). UV–vis [CH_2_Cl_2_, λ_max_, nm (ε, *M*^–1^·cm^–1^)]: ∼338 (sh, ∼6600), 378 (2300). MS (ESI^+^) Calcd for [C_22_H_30_N_2_PdS_6_]^+^: *m*/*z* 620.9851; Observed: *m*/*z* 620.9839; Error (δ) 2.07 ppm. Anal. Calcd for C_22_H_30_N_2_S_6_Pd: C, 42.53; H, 4.87; N, 4.51. Found: C, 42.49; H, 4.86; N, 4.50.

##### Method B

In a 50 mL Schlenk flask, **2** (0.1080 g, 0.1957 mmol) was dissolved in 10 mL of dry CH_2_Cl_2_ under an atmosphere of N_2_. To this mixture, solid [(mnt)SnMe_2_] (0.0577 g, 0.200 mmol) was added under an outward flow of N_2_. While the mixture was stirred at room temperature overnight, it turned dark green. All volatiles were removed *in vacuo*, and the solid residue was purified on silica chromatography column packed with 1:1 hexanes/CH_2_Cl_2_ and eluted with 1:2 hexanes/CH_2_Cl_2_. The product was collected as the leading deep blue band. After the removal of the solvents, the solid residue was crystallized by a layered diffusion of MeOH into a CH_2_Cl_2_ solution. Yield: 0.0782 g, 0.126 mmol, 64.3%.

#### [(mnt)Pt(tptbz)], **6**

In a 25 mL Schlenk flask, PtCl_2_ (0.1046 g, 0.393 mmol) was combined with MeCN (10 mL) under a N_2_ atmosphere and heated until a clear yellow solution was attained. This solution was then cooled to room temperature, whereupon solid tptbz (0.1493 g, 0.398 mmol) was added, and stirring was continued at ambient temperature overnight. The solvent was then removed *in vacuo*, and the solid residue was redissolved in CH_2_Cl_2_ (10 mL). Solid [(mnt)SnMe_2_] (0.1212 g, 0.419 mmol) was added, and stirring was maintained for an additional 12 h. The reaction mixture was then gravity filtered through paper, and the solid material thus separated was washed with further CH_2_Cl_2_ (40 mL). The filtrate was taken to dryness under reduced pressure, and the resulting solid residue was purified on a silica gel column that was packed as a slurry in, and eluted with, 1:1 CH_2_Cl_2_/hexanes. Compound **6** was collected as the leading orange-red band and crystallized by diffusion of Et_2_O vapor into a CH_2_Cl_2_ solution. Yield: 0.1516 g, 54.3%. ^1^H NMR (δ, CDCl_3_): 7.59 (s, 2 H aromatic C–*H*), 3.66–3.55 (m, 2 H, SC*H*(CH_3_)_2_, *J*_H–H_ = 6.7 Hz), 3.53–3.46 (m, 2 H, SC*H*(CH_3_)_2_, *J*_H–H_ = 6.7 Hz), 1.41 (d, 20 H, SCH(C*H*_3_)_2_, *J*_H–H_ = 6.7 Hz), 1.21–1.17 (m, 4 H, SCH(C*H*_3_)_2_). UV–vis [CH_2_Cl_2_, λ_max_, nm (ε, *M*^–1^·cm^–1^)]: ∼294 (sh, ∼32,000), ∼333 (sh, 8200), 368 (6800). Calcd *m*/*z* for [M + H]^+^: 711.045431; Found: 711.04456; Error: 1.22 ppm. Anal. Calcd for C_22_H_30_N_2_S_6_Pt: C, 37.22; H, 4.26; N, 3.95; S, 27.10. Found: C, 37.21; H, 4.27; N, 3.94; S, 27.06.

#### [(pdt)Sn^*n*^Bu_2_], **7**

To a solution of NaOMe (0.151 g, 2.80 mmol) in 20 mL of dry MeOH in a 50 mL Schlenk flask was added solid 4,5-diphenyl-1,3-dithiol-2-one (0.300 g, 1.11 mmol) under an active flow of N_2_. The mixture was left to stir at ambient temperature for 2 h, at which point solid ^*n*^Bu_2_SnCl_2_ (0.337 g, 1.11 mmol) was then added. Stirring was continued overnight, and all volatiles were then removed *in vacuo*. The solid residue was dissolved in CH_2_Cl_2_ (20 mL). This solution was filtered through packed Celite to remove the finely suspended NaCl byproduct, and the filtrate was then reduced to a volume of ∼4 mL. Hexanes (40 mL) were slowly introduced via a syringe, which induced the formation of the product as a yellow crystalline solid. Yield: 0.115 g, 21.8%. ^1^H NMR (δ, CDCl_3_): 7.17–6.96 (m, 10 H, aromatic C–*H*), 1.92–1.75 (m, 4 H, C*H*_2_), 1.74–1.64 (m, 4 H, C*H*_2_), 1.54–1.34 (m, 4 H, C*H*_2_), 0.97 (t, 6 H, *J*_H–H_ = 7.3 Hz, C*H*_3_). MS (ESI^+^) Calcd for [C_22_H_28_S_2_Sn]^+^: *m*/*z* 477.0733; Observed: *m*/*z* 477.0735; Error (δ) 0.32 ppm.

#### [(pdt)Pt(tptbz)], **8**

In a 50 mL Schlenk flask, PtCl_2_ (0.1262 g, 0.4745 mmol) and 20 mL of MeCN were combined under a N_2_ atmosphere and heated at ∼80 °C until a clear yellow solution formed. To this mixture was added solid tptbz (0.1782 g, 0.476 mmol), and the mixture was left to stir overnight at this same temperature. The solvent was then removed *in vacuo*, and the remaining solid residue was combined with dry CH_2_Cl_2_ (20 mL) followed by solid [(pdt)Sn^*n*^Bu_2_] (0.2295 g, 0.483 mmol). The resulting red mixture was stirred at ambient temperature overnight under N_2_, after which time the solvent was removed under reduced pressure and the remaining solid residue was washed with MeOH (20 mL) and Et_2_O (2 × 20 mL). Purification was accomplished on a silica gel column eluted with 1:1 CH_2_Cl_2_/hexanes. Following the collection of a leading purple band, **8** emerged as the subsequent orange-red band. After the removal of the solvent mixture, **8** was crystallized as deep orange-red crystals after diffusion of Et_2_O into a CH_2_Cl_2_ solution. Yield: 0.1099 g, 0.1353 mmol, 28.6%. *R_f_* = 0.71 (CH_2_Cl_2_). ^1^H NMR (δ, CDCl_3_): 7.58 (s, 2 H, tptbz aromatic C–*H*), 7.37–7.29 (m, 4 H, phenyl C–*H*), 7.18–7.05 (m, 6 H, phenyl C–*H*), 3.57 (sept, 4 H, *J*_H–H_ = 6.7 Hz, SC*H*(CH_3_)_2_), 1.44–1.32 (m, 24 H, SCH(C*H*_3_)_2_). UV–vis [CH_2_Cl_2_, λ_max_, nm (ε, *M*^–1^·cm^–1^)]: ∼335 (sh, 8050). MS (ESI^+^) Calcd for [C_32_H_40_S_6_Pt]^+^: *m*/*z* = 812.1101; Observed: *m*/*z* 812.108; Error (δ) 2.61 ppm.

#### [[Cu(tptbz)][PF_6_]]_*n*_, **9**

To a Schlenk flask with a stirring solution of [Cu(MeCN)_4_][PF_6_] (0.398 g, 1.07 mmol) in CH_2_Cl_2_ (10 mL), solid tptbz (0.4009 g, 1.070 mmol) was added under an outward flow of N_2_ followed by additional CH_2_Cl_2_ (10 mL). The reaction mixture became turbid, and a white precipitate quickly formed. The mixture was left to stir at ambient temperature overnight, after which time the solid residual was collected by filtration, washed with fresh CH_2_Cl_2_ (2 × 10 mL), and dried under vacuum. Yield: 0.2763 g, 0.474 mmol, 44.3%. Crystallization of polymeric **9** as colorless tablets was accomplished by the diffusion of Et_2_O into a DMF solution. ^1^H NMR (δ, DMSO-*d*_6_): 7.78 (s, 2 H, aromatic C–*H*), 3.95–3.64 (m, 4 H, SC*H*(CH_3_)_2_), 1.24 (d, 24 H, *J*_H–H_ = 6.5 Hz, SCH(C*H*_3_)_2_).

### Syntheses and Structures

**Scheme 2:**
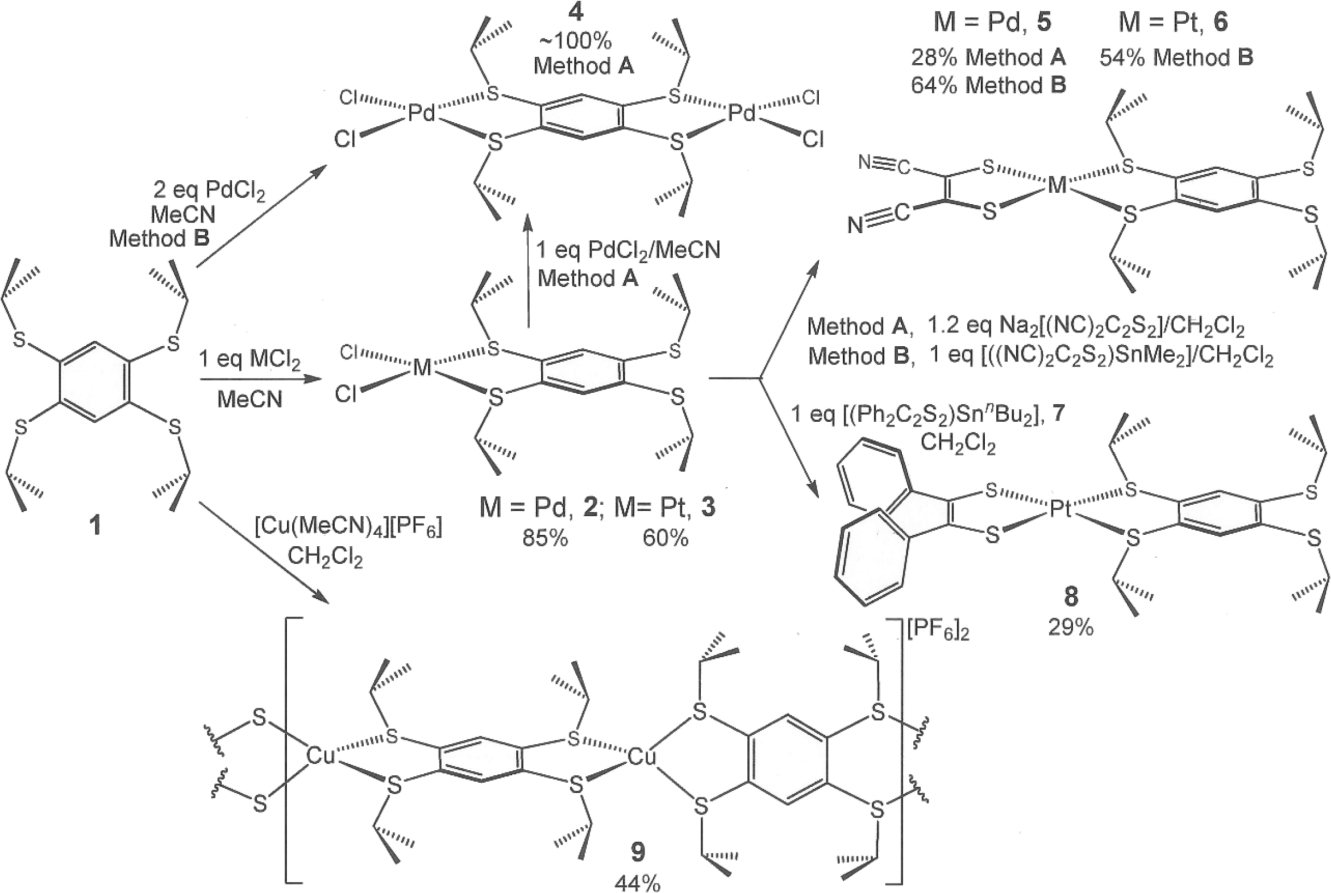
Synthesis of 1,2,4,5-Tetrakis(isopropylthio)benzene Compounds

**Figure 2:**
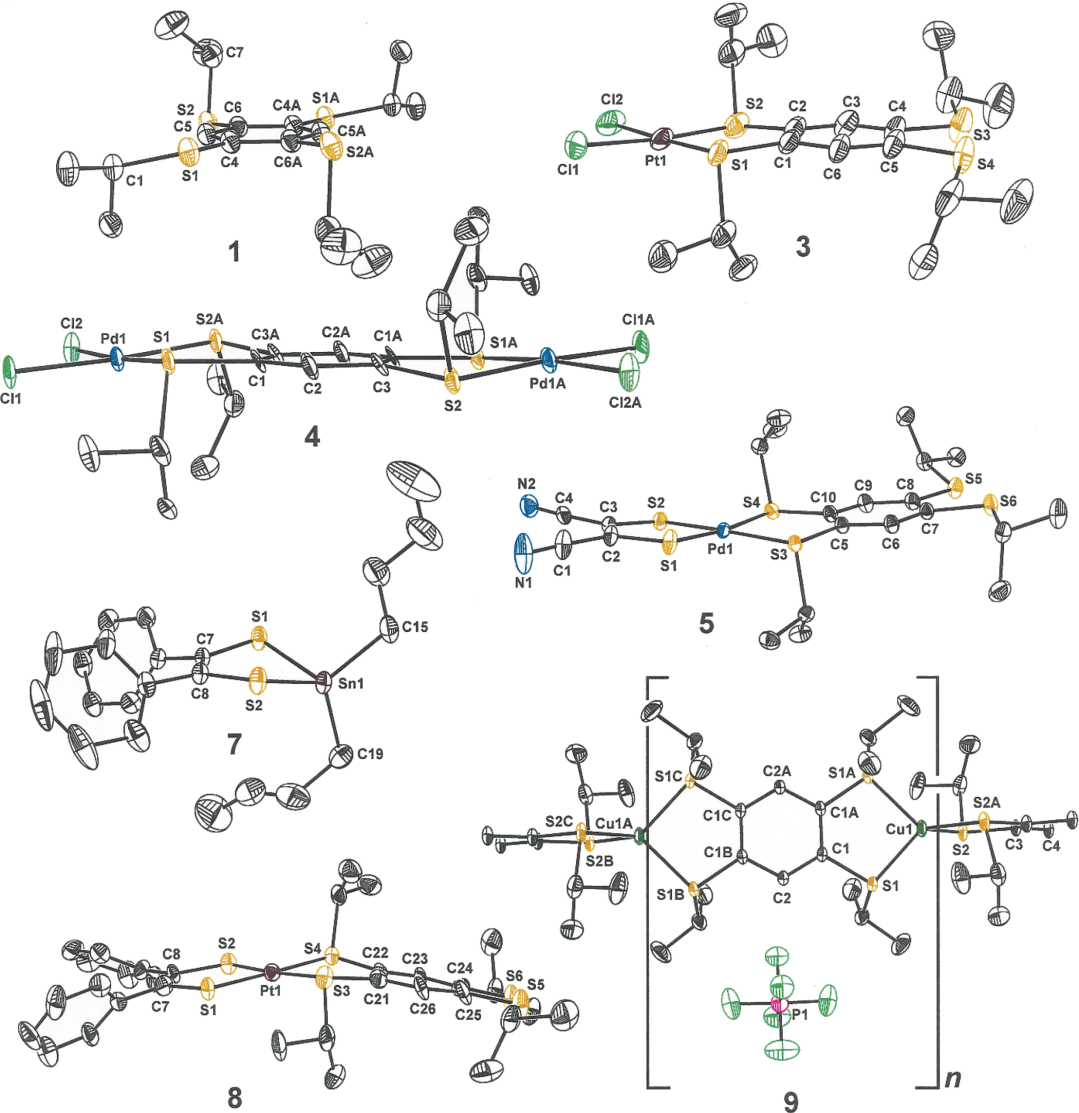
Thermal ellipsoid plots (50% ellipsoids) of selected compounds characterized by X-ray diffraction. For clarity, all H atoms are omitted, and disorder in the alkyl groups of **5** and **7** is not shown.

The tptbz ligand (**1**; Scheme 2) is attained in good yield from the corresponding 1,2,4,5-tetrachlorobenzene by straightforward reaction with ^*i*^PrS^–^Na^+^ in *N*,*N*-dimethylformamide^8^ or hexamethylphosphoramide.^13^ Although this approach is general in leading to arene tetrathioethers, other alkyl thiolates are less conveniently obtained (e.g., MeS^–^) or less conducive to the formation of the crystalline material (e.g., ^*n*^BuS^–^). Slow evaporation of an acetonitrile solution of **1** reliably deposited columnar crystals in monoclinic *P*2_1_/*c* (no. 14) with the central arene ring coincident with a crystallographic inversion center (Figure 2). The S–C_arene_ bond lengths in **1** are ∼0.06 Å shorter than the S–C_*i*Pr_ bond lengths, likely reflecting the effect of S p-π arene-π interaction. An earlier structural characterization reported for **1** was conducted at a somewhat higher temperature (200 vs 150 K) than that used here.^14^

Surprisingly little well-defined coordination chemistry has been described with 1,2,4,5-arene tetrathioethers in general, and with tptbz in particular and its simpler homologue 1,2-bis(*iso*propylthio)benzene, no metal complexes of which we are aware have been reported. As expected for a chelating dithioether, the tptbz ligand shows affinity primarily for soft or borderline soft late transition metals. Thus, when introduced to 1 equiv of tptbz, both PdCl_2_ and PtCl_2_ are readily coordinated at one end, while NiCl_2_ resists binding under the same conditions. Dithioether ligation for Ni^2+^ is known, but the majority of well-defined complexes that are known have this donor atom set incorporated within tetradendate^15−17^ or macrocyclic hexadentate ligands^18,19^ whose kinetic and thermodynamic advantages overcome the otherwise tepid Ni–S_thioether_ interaction. The use of 2 equiv of PdCl_2_ in the reaction with 1 equiv of tptbz led to the formation of the dimetallic [Cl_2_Pd(tptbz)PdCl_2_] (**4**), as marked by its immediate precipitation due to lowered solubility in consequence of its centrosymmetry.

Compounds **2** and **3** are isostructural (Table 1 and Figure 2) and reveal quite similar bond lengths and degrees of planarity (Table 2). The M–S_thioether_ bond lengths in these complexes are, within experimental resolution, shorter than the M–Cl bond lengths by 0.035 and 0.059 Å for Pd and Pt, respectively. This difference appears to reflect a modest contribution from the chelate effect, as the (Pt–Cl)_ave_ – (Pt–S)_ave_ difference in *cis*-[PtCl_2_(SMe_2_)_2_] is 0.047 Å.^20^ A modest difference with no clearly discernible explanation is the S–C_isopropyl_ distance at the metalated end (∼1.86 Å) vs the open end (∼1.79 Å). Compared to the S–C_isopropyl_ distance in the free tptbz ligand (∼1.83 Å), this difference appears to have equal contribution from S–C_isopropyl_ elongation at the metalated end and S–C_isopropyl_ contraction at the open end. The structure of **4** occurs on an inversion center in triclinic *P*-1, its unique half being effectively indistinguishable from **2** in all metrical details (Table 2). The intramolecular Pd···Pd separation of 8.651(1) Å is slightly shorter than the 8.857 Å observed for [Cl_2_Pd(tpbz)PdCl_2_].^2^

**Table 2:** Selected Interatomic Distances (Å) and Angles (deg.) for Crystallographically Characterized Compounds^a^

	tptbz	C_6_(S^*i*^Pr)_6_	[(pdt)Sn^*n*^Bu_2_]	
S–C_arene_	1.7695[14]	1.779[1]	Sn–S	2.4401[5]
S–C_*i*Pr_	1.8303[14]	1.838[1]	S–C	1.775[2]
δ^b^	0.074	0.119	Sn–C	2.142[3]
σ^c^	0.038–0.109	0.057–0.164	θ (deg)^d^	28.3

	[Cl_2_M(tptbz)]			
	M = Pd	M = Pt	[Cl_2_Pd(tpbzPdCl_2_]	
M–S	2.2718[13]	2.2528[12]	M–S	2.276[2]
M–Cl	2.3070[14]	2.3122[13]	M–Cl	2.307[2]
_M_S–C_*i*Pr_	1.859[6]	1.858[5]	_M_S–C_*i*Pr_	1.857[9]
_M_S–C_arene_	1.775[5]	1.788[4]	_M_S–C_arene_	1.789[9]
_open_S–C_*i*Pr_	1.779[8]	1.778[7]	Pd···Pd	8.651(1)
_open_S–C_arene_	1.765[5]	1.766[5]	Cl–M–Cl	90.77(11)
Cl–M–Cl	91.98(9)	90.35(8)	S–M–S	89.99(10)
S–M–S	90.13(6)	90.45(6)	θ^e^	4.9(2)
θ^e^	6.2(1)	4.9(1)		

	[(mnt)M(tptbz)]				
	M = Pd	M = Pt	[(pdt)Pt(tptbz)]^f^	[Cu(tptbz)]^+^_*n*_	
M–S_dithiolene_	2.2693[3]	2.2712[4]	2.2614[5]	Cu–S	2.2980[3]
M–S_thioether_	2.3246[3]	2.3025[4]	2.3095[5]	S–C_thioether_	1.7764[8]
S_dithiolene_–C	1.7370[11]	1.738[2]	1.764[2]	S–C_*i*Pr_	1.851[1]
_M_S_thioether_–C_arene_	1.7797[10]	1.780[1]	1.781[2]	Cu···Cu	8.619, 8.635
_M_S_thioether_–C_*i*Pr_	1.8721[11]	1.860(2)^g^	1.866[3]	S–Cu–S_chelate_	91.90[1]
_open_S_thioether_–C_arene_	1.7662[9]	1.757[1]	1.762[2]	θ (deg)^h^	88.96
_open_S_thioether_–C_*i*Pr_	1.8178[10]	1.825[2]	1.824[2]		
θ^i^	6.69(2)	5.46(4)	2.83[5]		

a Uncertainties are propagated according to Taylor, J. R. *An Introduction to Error Analysis*; 2nd ed.; University Science Books: Sausalito, CA, 1997, pp 73–77. The square bracket represents the uncertainties propagated in the averaging of chemically identical values.

b δ = mean displacement of S atoms from the C_6_ arene plane.

c σ = range of S atom displacements from the C_6_ arene plane.

d θ = fold angle between S_2_Sn and S_2_C_2_ planes.

e θ = angle between Cl_2_M and S_2_M planes.

f All values are averages for three independent molecules in the asymmetric unit of the cell.

g Because of static disorder afflicting one of the S*^i^*Pr groups, this table entry is a single, unaveraged bond length.

h θ = angle between S_2_Cu chelate planes.

i θ = angle between S_2_,_dithiolene_M and S_2,dithioether_M planes.

When introduced to [Cu(MeCN)_4_][PF_6_] in CH_2_Cl_2_, tptbz forms a 1D cationic coordination polymer (**9**; Scheme 2 and Figure 2) similar to that formed by hexakis(methylthio)benzene and Cu(I) and Ag(I).^21^ The intraligand tptbz bond lengths in **9** differ little from those of the free ligand. With the 1,2,4,5-tetramethylmercaptobenzene (tmmb) ligand, Cu(I) halide precursors yield halide-linked 1D or 2D polymers,^22^ as well as discrete halide-bridged dimetallic species,^23^ instead of homoleptic thioether polymers.

Compounds **2** and **3** are readily subject to halide substitution for dithiolene reactions via either salt metathesis or through the agency of tin-transmetallating compounds, whose usefulness in chloride-for-dithiolene exchange is well-established.^1−3,24−29^ Thus, [(mnt)Pd(tptbz)] (**5**) is produced in modest yield (28%) from the heterogeneous reaction between **2** and Na_2_mnt in CH_2_Cl_2_ but, as has been our consistent observation, in appreciably better yield (64%) via chloride-for-mnt^2–^ exchange with [(mnt)SnMe_2_]. The better margins produced by such tin reagents are likely due to the effect of greater homogeneity in the reaction solution. Similar reactions of **3** with [(mnt)SnMe_2_] and [(pdt)Sn^*n*^Bu_2_] (**7**) afforded [(mnt)Pt(tptbz)] (**6**) and [(pdt)Pt(tptbz)] (**8**). Compounds **5** and **6**, as well as their precursors **2** and **3**, are indefinitely stable to moisture and air, both in the solid state and in solution. In contrast, while **8** is amenable to purification by column chromatography and to crystallization in the open air, its solutions slowly deteriorate to a dark orange oily material over a period of days if not maintained in the dark under a protecting N_2_ atmosphere. Considering the quite extensive body of heteroleptic dithiolene compounds of the group 10 metals, which includes many dithiolene-dithione compounds,^30−33^ it is noteworthy that **5**, **6**, and **8** appear to be first heteroleptic dithiolene-dithioether complexes made by deliberate synthesis. A platinum bis(trifluoromethyl)dithiolene (tfd) dithioether complex, formed serendipitously by the addition of 2 equiv of 2,3-dimethyl-1,3-butadiene to [Pt(tfd)_2_], stands as the only prior example.^34^

Compounds **5**, **6**, and **8** retain the near ideal planarity of **2** and **3**, as gauged by θ values of ∼3–7° (Table 2). The principal metrical change in the immediate metal ion environment is a substantial lengthening of the M–S_thioether_ bond lengths by ∼0.05 Å compared to the precursor dichlorides (Table 2), which reflects the appreciably greater ligand field strength of the chelating dithiolene ligand. The Pt–S_dithiolene_ bond length in **8** is shorter than that observed in a series of [(pdt)Pt(C≡NR)_2_] complexes by a modest, but significant, amount within experimental resolution, ∼0.01 Å.^35^ The comparative binding weakness of tptbz that is implicated by this difference is borne out in the finding that **8** is not directly accessible from [(pdt)_2_Pt] by dithiolene displacement with tptbz, though such an approach is efficacious for the synthesis of a range of other heteroleptic dithiolene complexes of the group 10 metals.^33,35,36^

In the crystalline state, the packing arrangement for isostructural **5** and **6** is such that the square planar molecules form orderly, columnar stacks that arrange approximately along the *a*-axis of the monoclinic cell (Figure S23). Compound **8**, however, occurs in triclinic *P*-1 with an atypical three full molecules in the asymmetric unit such that *Z* = 6. The disposition of these separate molecules relative to one another is not simply described, as they are not coplanar or mutually orthogonal or related by any pseudosymmetry operation. Rather, the planes defined by their PtS_4_ coordination cores form angles of ∼56°, ∼64°, and ∼67° with one another that appear to be guided by intermolecular S_thioether_···S_thioether_ close contacts (Figure S24). Distances of 3.414 Å separating S(4) of molecule 1 from S(10) from molecule 2 and 3.508 Å between S(9) of molecule 2 and S(15) of molecule 3 are less than twice the crystallographic van der Waals radius for sulfur (1.8 Å)^37^ and suggest that soft–soft dispersion-type forces may be operative in the crystal packing. Furthermore, C–H···arene_centroid_ hydrogen bonding contacts likely play an additional role in dictating the packing, as seen in the pair of H-bonds that relate molecule 3 of compound **8** to its inversion counterpart (Figure S25).

### Electrochemistry and Electronic Structure

**Figure 3:**
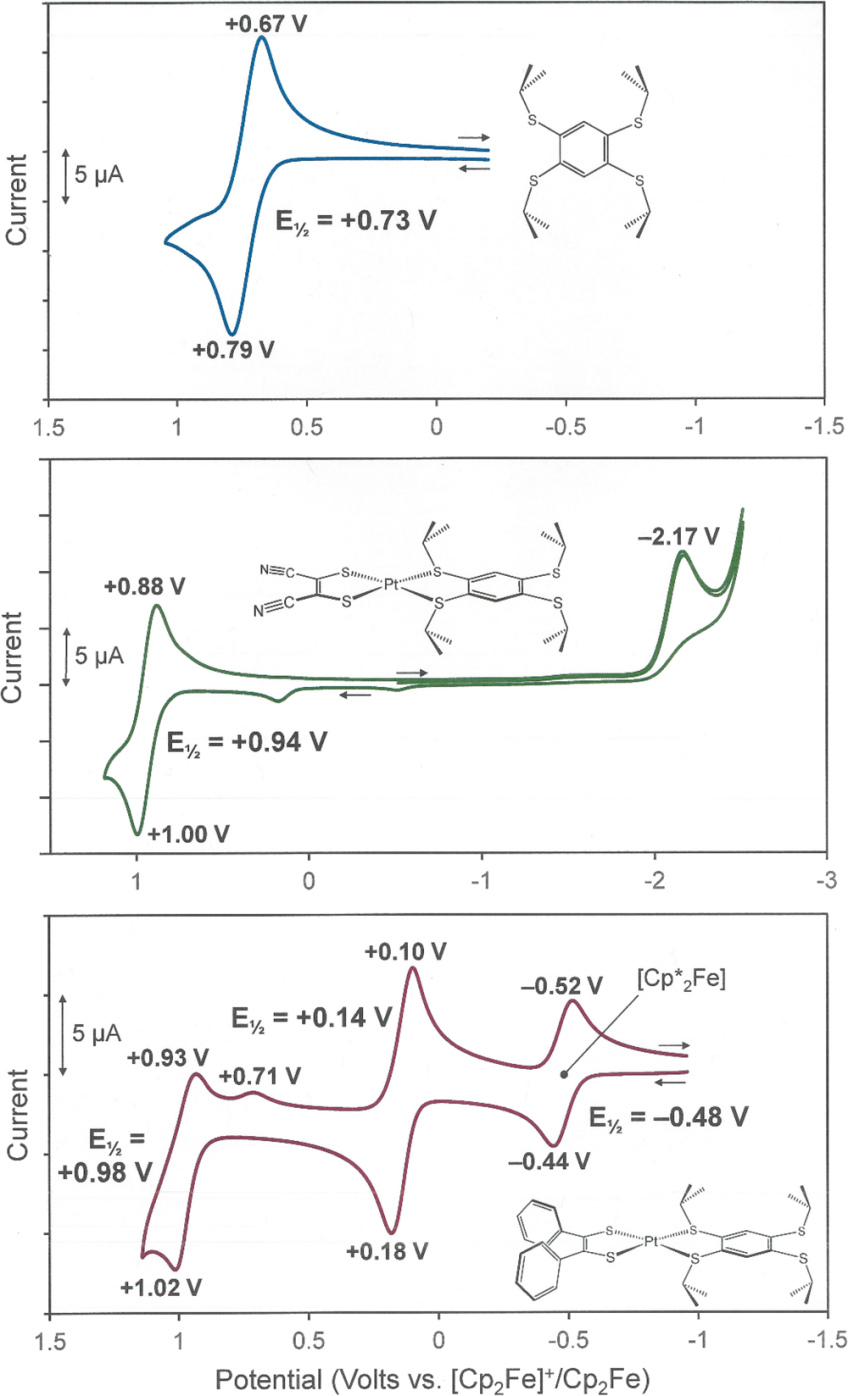
Cyclic voltammogram of the free tptbz ligand in CH_2_Cl_2_ with the [*^n^*Bu_4_N][PF_6_] supporting electrolyte (top). Cyclic voltammogram of [(mnt)Pt(tptbz)] revealing a 1-e^–^ oxidation that is largely dithiolene ligand-based (middle). Cyclic voltammogram of [(pdt)Pt(tptbz)] revealing successive oxidations that are assigned to the [Ph_2_C_2_S_2_]^2–^ ligand (bottom). Potentials are referenced to [Cp_2_Fe]^+^/Cp_2_Fe, but decamethyl ferrocene was used as an internal additive to avoid interference with the analyte.

The tptbz ligand sustains a reversible oxidation at +0.73 V vs Fc^+^/Fc (Figure 3, top) because the four thioether sulfur atoms collectively impart appreciable π-electron density to the arene ring. The oxidation potential for this ring system, when it is implemented as a chelating ligand, is anticipated to shift to higher potential owing to the diversion of two sulfur lone pairs in σ donation to a metal cation.

For [(mnt)Pd(tptbz)] (**5**) and [(mnt)Pt(tptbz)] (**6**), the nearly ∼3.5 V potential window that is accessible in CH_2_Cl_2_ reveals a single oxidation wave that is reversible in appearances for **6** at +0.94 V vs Fc^+^/Fc (Figure 3, middle) but only quasireversible for **5** at +1.00 V (Figure S47). Upon reversal of the scanning potential, **5** reveals a minor current maximum at +0.71 V, which, owing to the absence of a comparable feature on the initial anodic pass, is attributable to decomposition product forming on the timescale of the oxidative scanning. These oxidation processes for **5** and **6** are ene-1,2-dithiolate to radical monoanion oxidations (Scheme 1, (**a**) → (**b**)), as confirmed by the DFT calculation (Figure S49). The ∼0.06 V milder potential for the oxidation in **6** vs **5** likely is due to a greater degree of the modest metal d character that is mixed into this largely ligand-based HOMO. In the cathodic direction, **5** and **6** reveal irreversible reduction processes that are of a qualitatively similar irreversible appearance but positioned at quite different potentials. The cathodic current maximum for this wave occurs at −2.17 V for **6** vs −1.40 V for **5**(Figure S48). The LUMOs for **5** and **6,** which are the expected M-d_*x*^2^–y^2^_ S-p σ* interaction, reflect this difference in reduction potentials by their energies relative to the HOMO. The HOMO–LUMO gap calculated for **6** in the gas phase exceeds that for **5** by more than 0.5 eV. The higher energy for this σ* MO in **6** is fully in accordance with the greater ligand field strength for the third row metal vs its second row counterpart. For both **5** and **6**, irreversibility upon reduction likely is due to dissociation of the tptbz ligand.

Scanning anodically, the first redox process observed for [(pdt)Pt(tptbz)] occurs at +0.14 V versus Fc^+^/Fc and is attributed to the [Ph_2_C_2_S^–^_2_]^2–^ – e^–^→ [Ph_2_C_2_S^–^S^•^]^1–^ ligand oxidation (Figure 3, bottom). In heteroleptic [(pdt)ML_2_] complexes (M = Ni^2+^, Pd^2+^, Pt^2+^; L = phosphine, isonitrile), this oxidation occurs at ∼0 V vs Fc^+^/Fc with only modest variation as the identity of L is changed.^33,35,36^ The second, quasireversible anodic process for [(pdt)Pt(tptbz)] at +0.98 V has a plausible assignment either as a second oxidation of the phenyl dithiolene ligand ([Ph_2_C_2_S^–^S^•^]^1–^ – e^–^→ [Ph_2_(C=S)_2_]^0^), which is precedented in related compounds,^1,2^ or as a first oxidation of the tptbz ligand.

**Figure 4:**
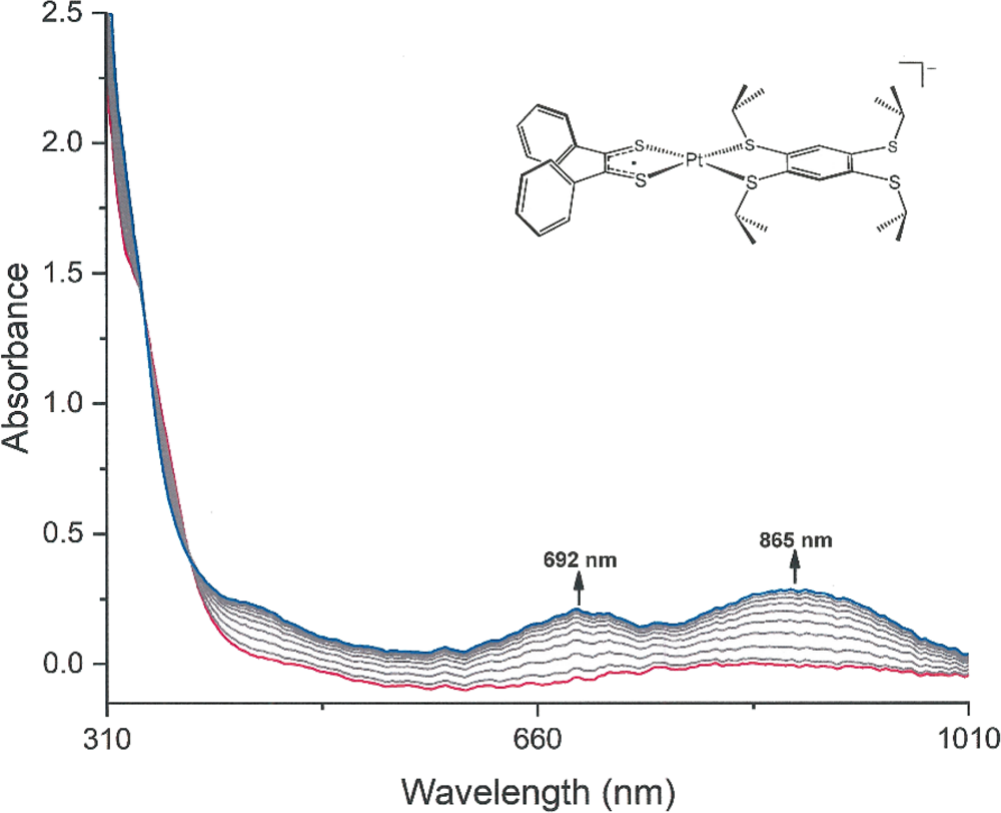
Spectroelectrochemically generated UV–vis absorption spectrum of [**8**]^+^ in CH_2_Cl_2_.

Spectroscopically, the generation of [**8**]^+^ by a controlled-potential one-electron oxidation is attended by the onset of broad, low-energy absorptions at ∼692 and ∼865 nm that likely conceal the presence of multiple unresolved excitations (Figure 4). Subsequent one-electron oxidation to [**8**]^2+^ at a higher potential induces the disappearance of these features. A TD-DFT simulation of this absorption spectrum points to the contribution of multiple transitions to these low-energy features, most of them involving the [Ph_2_C_2_S^–^S^•^]^1–^-based SOMO as the acceptor (Figure S50). The lowest-energy absorption is described most simply as an intraligand excitation from the π-systems of the Ph substituents, with a minor admixture of metal 5d character, to the C_2_S_2_ fragment of the ligand.

To assist the interpretation of the second oxidation process observed for **8**, calculations were undertaken upon [(pdt)Pt(tptbz)]^2+^ as a closed shell singlet, where the second oxidation is dithiolene based and produces the α-dithione form of the ligand, as a triplet with one spin on each ligand, and as a singlet diradical (broken symmetry) with an unpaired electron on each organic ligand but with opposite spin. Of these several scenarios, the closed shell singlet arising from successive oxidation of [Ph_2_C_2_S_2_]^2–^ to the dithione is assessed as modestly lower in energy by ∼0.5 kcal/mol than the open shell singlet diradical, and the desired triplet state is the highest energy configuration by ∼7 kcal/mol. Regardless of the net spin state, a dicationic form of [(pdt)Pt(tptbz)] that could be formulated as [(Ph_2_C_2_S^–^S^•^)Pt^2+^(tptbz^•,+^)]^2+^ is fundamentally interesting from the perspective of materials engineering and the potential for insight into how physical properties might be tailored with better control.

One suggestion arising from the electrochemistry depicted in Figure 3 is that oxidations from each of the two different organosulfur ligands might be observable if either the aromatic dithioether ligand is made more electron rich or the phenyl dithiolene ligand is rendered more electron deficient such that its second oxidation to the dithione ((**b**) → (**c**), Scheme 1) is disfavored relative to radical cation formation in the tetrathioarene ring. The known 1,2,3,4,5,6-hexakis(isopropylthio)benzene, C_6_(S^*i*^Pr)_6_, is a plausible candidate as more electron rich relative to tptbz and is prepared by a similar route. However, the oxidation potential found for C_6_(S^*i*^Pr)_6_ is nearly identical to that found for tptbz (Figure S46). That C_6_(S^*i*^Pr)_6_ is not more easily oxidized in proportion to its greater number of pendant sulfur atoms is possibly due to steric crowding of the S^*i*^Pr substituents around the ring periphery such that optimal S p-π arene-π overlap is impeded. The greater value of δ for C_6_(S^*i*^Pr)_6_ vs tptbz (Table 2) aligns with this supposition.

**Figure 5:**
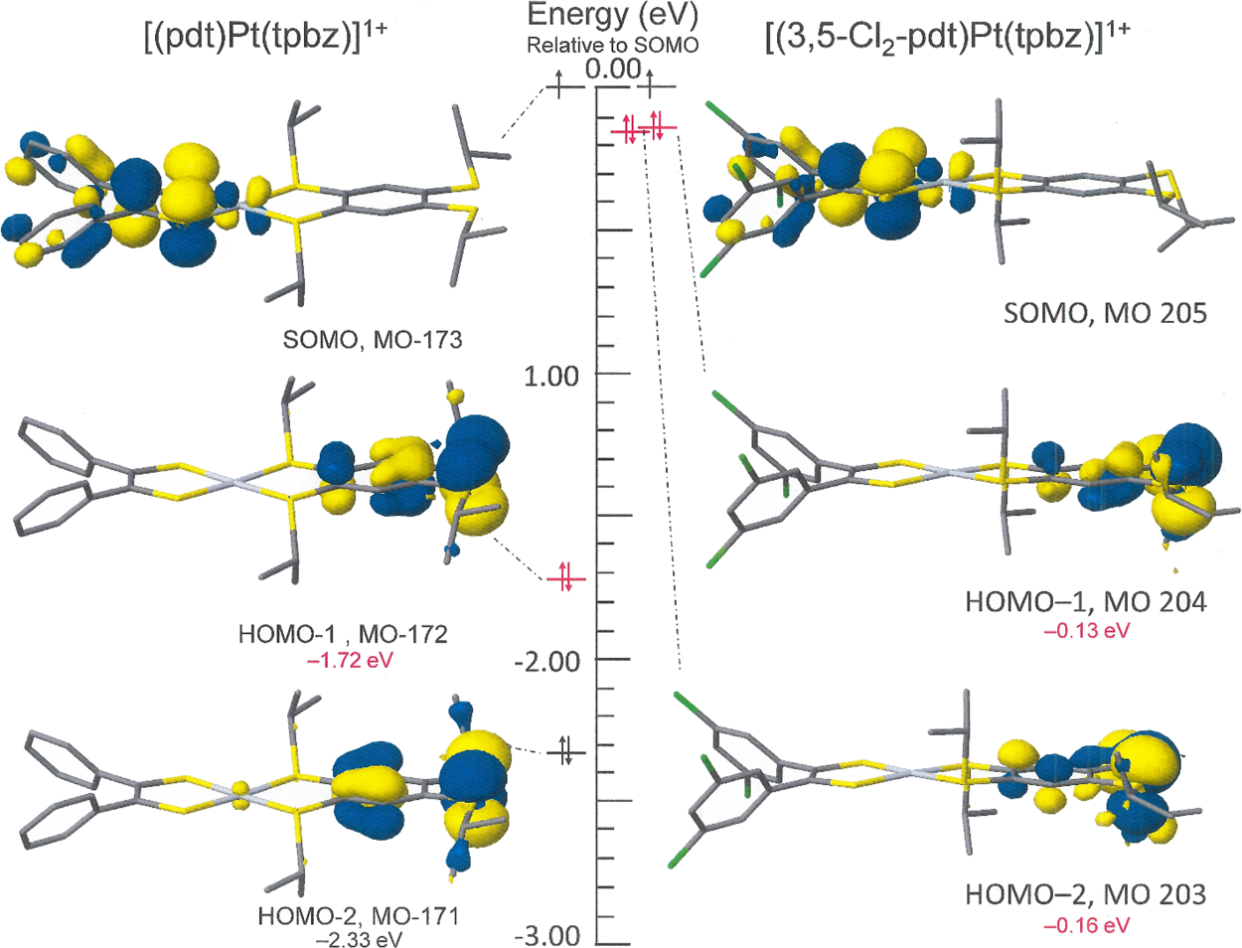
Relative energies of the highest occupied MOs for [(pdt)Pt(tptbz)]^+^ (left side) and [(3,5-Cl_2_-pdt)Pt(tptbz)]^+^ (right side). Orbital images are drawn at the 0.04 contour level.

Complexes featuring polychlorophenyl-substituted dithiolene ligands have not been reported, but they are likely accessible via the same benzoin/P_4_S_10_ route that has been implemented to prepare [Ni(S_2_C_2_(C_6_H_4_-*p*-Cl)_2_)_2_]^38,39^ and other complexes with arene-substituted dithiolene ligands. We have computationally investigated the 3,5-dichlorophenyl analogue of **8** for its effect in drawing downward in energy the dithiolene-based C_2_S_2_–HOMO and making the tptbz-based ligand competitive as the site of a second oxidation. Figure 5 illustrates the frontier MOs in the structures of gas-phase optimizations of both [(pdt)Pt(tptbz)]^+^ and [(Cl_2_-3,5-pdt)Pt(tptbz)]^+^. In [(pdt)Pt(tptbz)]^+^, the MO housing the dithiolene radical is ∼1.72 eV higher in energy than the tptbz-based HOMO–1 (Figure 5, left), but in [(Cl_2_-3,5-pdt)Pt(tptbz)]^+^, while the qualitative ordering is the same, the energy difference is substantially narrowed to ∼0.13 eV (Figure 5, right). This outcome suggests that, in [(Cl_3_-2,4,6-pdt)Pt(tptbz)]^+^, where the number and placement of chlorine substituents are such as to further lower the dithiolene-based MOs, successive one-electron oxidations would occur on the opposing dithiolene and dithioether ligands.

## Summary and Conclusions

The majority of coordination complexes featuring arene-1,2-dithioether ligation have this fragment incorporated within a larger, multidentate ligand architecture as typified by 1,2-bis(2-mercaptophenylthio)phenylene(2−),^40^ 1,2-bis(2-pyridylthio)phenylene,^40^ and various cyclic multithioether ligands.^18,19^ Simple bidentate arene dithioether ligands, such as 1,2-bis(2-methoxyethylthio)benzene, have been investigated for their potential in the selective extraction and recovery of Pd(II) from waste streams.^41^ However, perhaps because they are seen as ligands that favor the formation of inorganic polymers, as seen with [[M(C_6_(SMe)_6_)][PF_6_]]_*n*_ (M = Cu^+^, Ag^+^),^21^ 1,2,4,5-tetrathioether benzenes have received scant attention, if indeed any at all, for their possibilities in the systematic, “bottom-up” assembly of multimetal arrays or for their potential redox activity while chelated to a metal ion.

In this report, we have detailed the syntheses, structures, and properties of the monometallic, open-ended complexes [Cl_2_M(tptbz)] (M = Pd, Pt), dimetallic [Cl_2_Pd(tptbz)PdCl_2_], and the heteroleptic, open-ended dithiolene-dithioether complexes [(mnt)M(tptbz)] (M = Pd, Pt) and [(pdt)Pt(tptbz)]. Surprisingly, these complexes appear to be the first with tptbz itself and join only [(tmmb)Cu]_2_(μ-I)_2_^23^ as structurally authenticated coordination compounds with such 1,2,4,5-substituted arene tetrathioethers. While oxidation of coordinated tptbz to the radical cation is not observed in [(pdt)Pt(tptbz)], the observable redox chemistry being attributable to [Ph_2_C_2_S_2_]^2–^ alone, computational work suggests that phenyldithiolene variants whose oxidation potentials are rendered more positive may make tptbz oxidation experimentally accessible. In continuing work, we both explore the use of phenyldithiolene derivatives bearing electron-withdrawing groups for this end and target the synthesis of symmetric dimetallic [(R_2_C_2_S_2_)M(tptbz)M(S_2_C_2_R_2_)] complexes for comparison to their [(R_2_C_2_S_2_)M(tpbz)M(S_2_C_2_R_2_)] tetraphosphine analogues.

## Supporting Information Available

Procedures for crystal growth, X-ray diffraction data collection, and structure solution and refinement; description of computational procedures; tables summarizing unit cell and refinement data (Tables S1–S4); thermal ellipsoid plots with complete atom labeling (Figures S1–S22); crystal packing images (Figures S23–S25); analytical, spectroscopic, and electrochemical data for compounds reported (Figures S26–S48); MO energy level diagrams for **5** and **6** (Figure S49); calculated UV–vis spectrum for [**8**]^+^ (Figure S50); coordinates for optimized geometries (Tables S5–S18) (PDF)
