# Supplementary material for: Asymmetric by Design: Heteroleptic Coordination Compounds with Redox-Active Dithiolene and 1,2,4,5-Tetrakis(isopropylthio)benzene Ligands
Source: Inorg Chem. 2023 Dec 22;63(1):173–83. doi: 10.1021/acs.inorgchem.3c02928 (PMC10777400; doi:10.1021/acs.inorgchem.3c02928)
Supplement: Supplementary file 1 — ic3c02928_si_001.pdf [file ic3c02928_si_001.pdf]

Supporting Information for

Asymmetric by Design:
Heteroleptic Coordination Compounds with Redox-Active Dithiolene and 1,2,4,5-Tetrakis(isopropylthio)benzene Ligands

by

Che Wu,[†] Lakshmi Nishanth Kakarla,[‡] Chandru P. Chandrasekaran,[‡]
Xiaodong Zhang,[†] Joel T. Mague,[†]
Stephen Sproules,[§] and James P. Donahue[†,*]

[†]Department of Chemistry, Tulane University, 6400 Freret Street, New Orleans, LA 70118-5698, U.S.A.
[‡]Department of Chemistry and Biochemistry, Lamar University, Beaumont, TX 77710, U.S.A.
[§]WestCHEM, School of Chemistry, University of Glasgow, Glasgow G12 8QQ, United Kingdom.

Table of Contents

**Procedures for Crystal Growth, Collection and Processing of Diffraction Data, and Solving and Refining of Structures.**		S4-S6
**Computational Procedures.**		S6
**Table S1**.	Structure and refinement data for 1,2,4,5-(^{i}PrS)$_4$C$_6$H$_2$, 1,2,3,4,5,6-(^{i}Pr)$_6$C$_6$, C$_6$Cl(SCH$_2$C$_6$H$_5$)$_5$, and [(Ph$_2$C$_2$S$_2$)Sn^{n}Bu$_2$].	S7
**Table S2**.	Structure and refinement data for [Cl$_2$Pd(tptbz)], [Cl$_2$Pt(tptbz)], and [Cl$_2$Pd(tptbz)PdCl$_2$].	S8
**Table S3**.	Structure and refinement data for [(mnt)Pd(tptbz)], [(mnt)Pt(tptbz)], and [(pdt)Pt(tptbz)].	S9
**Table S4**.	Structure and refinement data for [[Cu(tptbz)][PF$_6$]·DMF]$_n$	S10
**Figure S1**.	Thermal ellipsoid plot with atom labeling for 1,2,4,5-(^{i}PrS)$_4$C$_6$H$_2$, View 1.	S11
**Figure S2**.	Thermal ellipsoid plot with atom labeling for 1,2,4,5-(^{i}PrS)$_4$C$_6$H$_2$, View 2.	S11
**Figure S3**.	Thermal ellipsoid plot with atom labeling for 1,2,3,4,5,6-(^{i}Pr)$_6$C$_6$.	S12
**Figure S4**.	Thermal ellipsoid plot with atom labeling for C$_6$Cl(SCH$_2$C$_6$H$_5$)$_5$, position 1	S13
**Figure S5**.	Thermal ellipsoid plot with atom labeling for C$_6$Cl(SCH$_2$C$_6$H$_5$)$_5$, position 2	S14
**Figure S6**.	Thermal ellipsoid plot of C$_6$Cl(SCH$_2$C$_6$H$_5$)$_5$ with interstitial $^1/_4$·CHCl$_3$	S15
**Figure S7**.	Thermal ellipsoid plot with atom labeling for [(Ph$_2$C$_2$S$_2$)Sn^{n}Bu$_2$].	S16
**Figure S8**.	Thermal ellipsoid plot with atom labeling for [Cl$_2$Pd(tptbz)].	S16
**Figure S9**.	Thermal ellipsoid plot (50%) with atom labeling for [Cl$_2$Pt(tptbz)], molecule 1 in triclinic polymorph	S17
**Figure S10**.	Thermal ellipsoid plot (50%) with atom labeling for [Cl$_2$Pt(tptbz)], molecule 2 in triclinic polymorph	S17
**Figure S11**.	Thermal ellipsoid plot (30%) with atom labeling for [Cl$_2$Pt(tptbz)] in monoclinic polymorph	S18
**Figure S12**.	Thermal ellipsoid plot with atom labeling for [Cl$_2$Pd(tptbz)Cl$_2$].	S19
**Figure S13**.	Thermal ellipsoid plot with atom labeling for interstitial DMI in [Cl$_2$Pd(tptbz)Cl$_2$]·2DMI.	S19
**Figure S14**.	Thermal ellipsoid plot with atom labeling for [((NC)$_2$C$_2$S$_2$)Pd(tptbz)].	S20
**Figure S15**.	Thermal ellipsoid plot with atom labeling for [((NC)$_2$C$_2$S$_2$)Pt(tptbz)].	S20
**Figure S16**.	Thermal ellipsoid plot with atom labeling for [(Ph$_2$C$_2$S$_2$)Pt(tptbz)], molecule 1.	S21
**Figure S17**.	Thermal ellipsoid plot with atom labeling for [(Ph$_2$C$_2$S$_2$)Pt(tptbz)], molecule 2.	S21
**Figure S18**.	Thermal ellipsoid plot with atom labeling for [(Ph$_2$C$_2$S$_2$)Pt(tptbz)], molecule 3.	S22
**Figure S19**.	Packing arrangement in asymmetric unit for [(Ph$_2$C$_2$S$_2$)Pt(tptbz)].	S22
**Figure S20**.	Coordination polymer strand 1 of [Cu(tptbz)][PF$_6$] with atom labeling.	S23
**Figure S21**.	Coordination polymer strand 2 of [Cu(tptbz)][PF$_6$] with atom labeling.	S24
**Figure S22**.	Interstitial DMF in [[Cu(tptbz)][PF$_6$]·DMF]$_n$.	S25
**Figure S23**.	Crystal packing diagram for **5**, view along the *a* axis of the cell.	S26
**Figure S24**.	Arrangement of molecules of **8** in the asymmetric unit with S···S intermolecular contacts shown.	S27
**Figure S25**.	Illustration of intermolecular C–H···arene$_{centroid}$ H-bonding interactions for **8**.	S28
**Figure S26**.	Elemental analysis request form for [Cl$_2$Pd(tptbz)]	S29
**Figure S2**7	Elemental analysis results for [Cl$_2$Pd(tptbz)]	S30
**Figure S28**.	Elemental analysis request form for [Cl$_2$Pt(tptbz)]	S31
**Figure S29**.	Elemental analysis results for [Cl$_2$Pt(tptbz)]	S32

Table of Contents, Continued

**Figure S30**.	Elemental analysis request form for [(mnt)Pd(tptbz)]	S33
**Figure S31**.	Elemental analysis request form for [(mnt)Pt(tptbz)]	S34
**Figure S32**.	Elemental analysis results for [(mnt)Pd(tptbz)] and [(mnt)Pt(tptbz)]	S35
**Figure S33**.	^{1}H NMR spectrum ($CDCl_3$) of $C_6(S^{i}Pr)_6$.	S36
**Figure S34**.	^{13}C NMR spectrum ($CDCl_3$) of $C_6(S^{i}Pr)_6$.	S37
**Figure S35**.	^{1}H NMR spectrum of [Cl_2Pd(tptbz)] in DMSO-d_6.	S38
**Figure S36**.	^{1}H NMR spectrum of [Cl_2Pt(tptbz)] in DMSO-d_6.	S39
**Figure S37**.	^{1}H NMR spectrum (DMSO-d_6) of [Cl_2Pd(tptbz)PdCl_2]	S40
**Figure S38**.	^{1}H NMR spectrum of [(mnt)Pd(tptbz)] in $CDCl_3$.	S41
**Figure S39**.	^{1}H NMR spectrum of [(mnt)Pt(tptbz)] in $CDCl_3$.	S42
**Figure S40**.	^{1}H NMR spectrum of [(pdt)Pt(tptbz)] in $CDCl_3$.	S43
**Figure S41**.	^{1}H NMR spectrum of [(pdt)$Sn^{n}Bu_2$] in $CDCl_3$.	S44
**Figure S42**.	^{1}H NMR spectrum of [[Cu(tptbz)][PF_6]]$_n$ in DMSO-d_6.	S45
**Figure S43**.	UV-vis spectrum of [(mnt)Pd(tptbz)] in CH_2Cl_2.	S46
**Figure S44**.	UV-vis spectrum of [(mnt)Pt(tptbz)] in CH_2Cl_2.	S47
**Figure S45**.	UV-vis spectrum of [(pdt)Pt(tptbz)] in CH_2Cl_2.	S48
**Figure S46**.	CV of $C_6(SiPr)_6$ in CH_2Cl_2.	S49
**Figure S47**.	CV of [(mnt)Pd(tptbz)], anodic direction, 100 mV/sec.	S50
**Figure S48**.	CV of [(mnt)Pd(tptbz)], full potential window, 100 mV/sec.	S51
**Figure S49**.	MO energy level diagram for [(mnt)M(tptbz)] (M = Pd, Pt).	S52
**Figure S50**.	Calculated UV-vis spectrum of [(pdt)Pt(tptbz)]$^{+}$.	S53
**Table S5**.	Atomic coordinates for optimized [($Ph_2C_2S_2$)Pt(tptbz)].	S54-S55
**Table S6**.	Atomic coordinates for optimized [($Ph_2C_2S_2$)Pt(tptbz)]$^{1+}$.	S56-S57
**Table S7**.	Atomic coordinates for optimized [($Ph_2C_2S_2$)Pt(tptbz)]$^{2+}$, closed singlet	S58-S59
**Table S8**.	Atomic coordinates for optimized [($Ph_2C_2S_2$)Pt(tptbz)]$^{2+}$, singlet diradical	S60-S61
**Table S9**.	Atomic coordinates for optimized [($Ph_2C_2S_2$)Pt(tptbz)]$^{2+}$, triplet	S62-S63
**Table S10**.	Atomic coordinates for optimized [(3,5-Cl_2-$Ph_2C_2S_2$)Pt(tptbz)].	S64-S65
**Table S11**.	Atomic coordinates for optimized [(3,5-Cl_2-$Ph_2C_2S_2$)Pt(tptbz)]$^{1+}$.	S66-S67
**Table S12**.	Atomic coordinates for optimized [(3,5-Cl_2-$Ph_2C_2S_2$)Pt(tptbz)]$^{2+}$, closed singlet	S68-S69
**Table S13**.	Atomic coordinates for optimized [(3,5-Cl_2-$Ph_2C_2S_2$)Pt(tptbz)]$^{2+}$, singlet diradical	S70-S71
**Table S14**.	Atomic coordinates for optimized [(3,5-Cl_2-$Ph_2C_2S_2$)Pt(tptbz)]$^{2+}$, triplet	S72-S73
**Table S15**.	Atomic coordinates for optimized [($(NC)_2C_2S_2$)Pd(tptbz)]^{0}	S74-S75
**Table S16**.	Atomic coordinates for optimized [($(NC)_2C_2S_2$)Pd(tptbz)]$^{1+}$	S76-S77
**Table S17**.	Atomic coordinates for optimized [($(NC)_2C_2S_2$)Pt(tptbz)]^{0}	S78-S79
**Table S18**.	Atomic coordinates for optimized [($(NC)_2C_2S_2$)Pt(tptbz)]$^{1+}$	S80-S81
**References**		S82

Procedures for Crystal Growth, Collection and Processing of Diffraction Data, and Solving and Refining of Structures.

Ligand and solvent abbreviations used in the following procedural description are mnt(2–) = maleonitriledithiolate(2–) = $[(NC)_2C_2S_2]^{2-}$; pdt(2–) = 1,2-diphenylethylene-1,2-dithiolate(2–) = $[Ph_2C_2S_2]^{2-}$; tptbz = 1,2,4,5-tetrakis(*iso*propylthio)benzene; DMI = 1,3-dimethyl-2-imidazolidinone. In the context of preparing hexakis(alkylthio)benzenes, as noted in the main manuscript, the reaction of benzylthiolate(1–) with C_6Cl_6 produced 1-Cl-2,3,4,5,6-pentakis(benzylthio)benzene, which was in mixture with other products and only identified structurally.

The crystalline sample of tptbz (colorless columns) used for the collection of X-ray diffraction data was obtained by the slow evaporation of a MeCN solution, while crystals of 1,2,3,4,5,6-hexakis(*iso*propylthio)benzene (yellow plates) deposited as the evaporite of a Et_2O solution. A layered diffusion of hexanes into a CH_2Cl_2 solution yielded [(pdt)Sn^{n}Bu$_2$] as colorless blocks, and similarly, layered diffusion of MeOH into $CHCl_3$ produced 1-Cl-2,3,4,5,6-pentakis(benzylthio)benzene·0.4$CHCl_3$ as colorless needles. All other samples were prepared by the small scale vial-in-a-vial vapor diffusion technique, where the solvent/diffusing vapor pair were as follows: [Cl_2Pd(tptbz)] (yellow slats): CH_2Cl_2/Et_2O; triclinic [Cl_2Pt(tptbz)] (colorless plates): CH_2Cl_2/*n*-pentane; monoclinic [Cl_2Pt(tptbz)] (light yellow plates): acetone/Et_2O; [Cl_2Pd(tptbz)Cl_2]·2DMI (yellow bars): DMI/Et_2O; [(mnt)Pd(tptbz)] (light green plates): CH_2Cl_2/*n*-pentane; [(mnt)Pt(tptbz)] (pale yellow columns): CH_2Cl_2/Et_2O; [(pdt)Pt(tptbz)] (thick yellow plates): 1,2-$ClCH_2CH_2Cl$/hexanes; [[Cu(tptbz)][PF_6]·DMF]$_n$ (colorless tablets): *N*,*N*-dimethylformamide/Et_2O.

All crystals were coated with paratone oil and mounted on the end of a nylon loop attached to the end of the goniometer. Data were collected at 150 or 160 K under a dry N_2 stream supplied under the control of an Oxford Cryostream 800 attachment. The data collection instrument was either a Bruker Smart APEX II CCD diffractometer equipped with a Mo fine-focus sealed tube providing radiation at λ = 0.71073 nm, a Bruker D8 Quest Photon 3 diffractometer that similarly operated with the Mo Kα 0.71073 nm light source, or a Bruker D8 Venture fitted with a Photon 100 CMOS detector and operating with Cu Kα radiation at λ = 1.54178 nm.

The data set for tptbz was collected as a programmed routine of 3 sets of 400 frames, each of 0.50° width in ω, collected at φ = 0.00°, 90.00°, and 180.00°, and 2 sets of 800 frames, each of

0.45° width in φ, collected at ω = –30.00° and 210.00.° The scan time was 20 sec/frame. The data sets for 1,2,3,4,5,6-hexakis(*iso*propylthio)benzene (6 sets of 220 frames and 1 set of 450 frames, 20 sec/frame), 1-Cl-2,3,4,5,6-pentakis(benzylthio)benzene·0.4$CHCl_3$ (7 sets of 368-371 frames, 30 or 60 sec/frame), [(pdt)$Sn^{n}Bu_2$] (13 x 336 frames and 2 x 720 frames, 10 sec/frame), [Cl_2Pd(tptbz)] (5 x 336 frames and 1 x 720 frames, 30 sec/frame), [Cl_2Pd(tptbz)Cl_2]·2DMI (6 sets of 176 frames and 2 sets of 360 frames, 10 sec/frame), [(mnt)Pd(tptbz)] (10 x 335-336 and 2 x 720 frames, 15 sec/frame), triclinic [Cl_2Pt(tptbz)] (5 sets of 336 frames, 90 sec/frame), monoclinic [Cl_2Pt(tptbz)] (8 x 358 frames and 2 x 720 frames, 10 sec/frame), [(mnt)Pt(tptbz)] (9 x 336 frames and 2 x 720 frames, 10 sec/frame), [(pdt)Pt(tptbz)] (7 x 358 frames, 20 sec/frame), and [[Cu(tptbz)][PF_6]·DMF]$_n$ (7 x 358 and 2 x 720 frames, 10 sec/frame) were collected with 0.5° width in ω or ϕ with scan parameters determined by *QUEEN* or by the "strategy" routine within *APEX2*/*APEX3*/*APEX4*.[1] For 1,2,3,4,5,6-hexakis(*iso*propylthio)benzene, a frame width of 0.8° rather than 0.5° was used.

Raw data were reduced to F^2 values using *SAINT*,[2] and a global refinement of unit cell parameters was performed using ~9300–9900 selected reflections from the full data sets. In the lone case of tptbz, a smaller set of 4974 reflections was used for the final unit cell refinement. For [Cl_2Pt(tptbz)], analysis of 1026 reflections having I/σ(I) > 15 and chosen from the full data set with *CELL_NOW*[3] (Sheldrick, 2008*b*) showed the crystal to belong to the triclinic system and to be twinned by a 180° rotation about *a*. A similar analysis of 3716 reflections having I/σ(I) > 25 from the full data set for [(pdt)Pt(tptbz)] with *CELL_NOW* (Sheldrick, 2008) showed the crystal to belong to the triclinic system but to be twinned by a 180° rotation about *b**. In both cases, the raw data were processed using the multi-component version of *SAINT* under control of the two-component orientation file generated by *CELL_NOW*, and an absorption correction was applied using the *TWINABS* routine.[4] All other data sets were corrected for absorption on the basis of multiple measurements of symmetry equivalent reflections or by numerical methods with the use of *SADABS*,[5] as described by Krause *et al*.[6]

All structure solutions were obtained by direct methods using *SHELXS*[7] or *SHELXT*,[8] while refinements were accomplished by full-matrix least-squares procedures using *SHELXL*.[9] The *SHELXL* program is incorporated into the both the *SHELXTL*[10] and *APEX*[1] software suites. In most instances, refinement was routine. In the case of 1-Cl-2,3,4,5,6-pentakis(benzylthio)benzene·0.4$CHCl_3$, four of the five benzyl groups, as well as the interstitial

$CHCl_3$ molecule, were each disordered over two sites. This disorder was addressed using a split atom model with floating site occupancies whose values were determined as a best fit by the refinement software. Other minor disorder in the ^{i}PrS groups of some complexes was similarly handled. For [(pdt)Sn^{n}Bu$_2$], the absolute structure was identified by refining the Flack parameter[11] to essentially zero (–0.004(4)). All H atoms were added in calculated positions with isotropic displacement parameters 1.2 - 1.5 times those of the carbon atoms to which they were attached. Using the AFIX 137 option, methyl carbons were allowed to freely rotate about the CH_3–CH bond so as to find the angular placement that best fit the electron density. All images were created using the graphics program *XP*, which is a routine contained within *SHELXTL*. All structures were checked for overlooked symmetry and other errors by the checkCIF service provided by the International Union of Crystallography.[12]

Computational Details

The density functional theory (DFT) calculations were carried out at the supercomputing facility at Tulane University using the *Gaussian-09* package.[13] Geometry optimizations of structures of neutral molecules were executed without symmetry restrictions beginning with the coordinates of the molecules from the X-ray crystallographic data. These optimized structures were used as initial input for optimizing the corresponding one-electron oxidized molecules. For the dicationic compounds, the optimized S = 1 structures served as a starting point for broken symmetry and closed shell S = 0 states. Additionally, for broken symmetry S = 0 optimizations of dications, fragmented inputs (formally $[R_2C_2S_2]^{\bullet-}Pt^{2+}[tptbz]^{\bullet+}$, R = Ph or 3,5-$Cl_2C_6H_3$) were used. The geometries were optimized at the Becke, three-parameter. Lee-Yang, Parr (B3LYP) level of theory[14,15] with typical basis sets. Frequency calculations were done to confirm the validity of the optimized structures. For platinum, a double-ζ (DZ) basis set with an effective core potential (LANL2DZ ECP) was implemented.[16] The 6-31G (d,p) basis set was chosen for carbon, whereas the triple-ζ (TZVP) was used for the heavier elements (S and Cl), and a Gaussian split valence (SV) basis set[17] was used for the hydrogen atoms. The molecular orbital (MO) plots were created using the Jmol program package.[18]

Table S1. Crystal and refinement data for 1,2,4,5-(^{i}PrS)$_4$C$_6$H$_2$, C$_6$(^{i}PrS)$_6$, C$_6$Cl(SBn)$_5$, and [(pdt)Sn^{n}Bu$_2$].

compound	1,2,4,5-(^{i}PrS)$_4$C$_6$H$_2$	C$_6$(^{i}PrS)$_6$	C$_6$Cl(SBn)$_5$	[(pdt)Sn^{n}Bu$_2$]
structure code	JTM560_0m_a	JPD1278_0m_a	JPD1288_a	JPD1137_0m_a
solvent	none	none	0.4CHCl$_3$	none
formula	$C_{18}H_{30}S_4$	$C_{24}H_{42}S_6$	$C_{41.40}H_{35.40}Cl_{2.20}S_5$	$C_{22}H_{28}S_2Sn$
FW	374.66	522.93	771.29	475.25
temperature, K	150	150	150	150
wavelength, Å	0.71073	0.71073	1.54178	0.71073
2θ range, deg.	4.074 - 57.586	4.818 – 53.106	6.620 - 136.56	4.476 - 66.450
crystal system	monoclinic	triclinic	monoclinic	tetragonal
space group	$P2_1/c$	P-1	$C2/c$	I-4
a, Å	10.1189(12)	9.1370(2)	58.285(3)	21.0089(3)
b, Å	8.3483(10)	9.6108(2)	5.0373(3)	21.0089(3)
c, Å	12.5378(15)	9.9046(2)	27.0057(12)	10.0948(2)
α, deg.	90	72.062(1)	90	90
β, deg.	98.834(2)	71.949(1)	108.735(4)	90
γ, deg.	90	63.875(1)	90	90
volume, Å^3	1046.6(2)	727.32(3)	7508.8(7)	4455.58(15)
Z	2	1	8	8
density, g/cm^3	1.189	1.194	1.365	1.417
μ, mm^{-1}	0.450	0.480	4.511	1.337
F(000)	404	282	3210	1936
crystal size	0.100 x 0.130 x 0.270	0.090 x 0.192 x 0.293	0.019 x 0.047 0.255	0.164 x 0.178 x 0.230
color, habit	colorless column	yellow plate	colorless needle	colorless block
limiting indices, h	$-13 \le h \le 13$	$-11 \le h \le 11$	$-70 \le h \le 68$	$-32 \le h \le 32$
limiting indices, k	$-11 \le k \le 10$	$-12 \le k \le 12$	$-5 \le k \le 6$	$-32 \le k \le 32$
limiting indices, l	$-16 \le l \le 16$	$-12 \le l \le 12$	$-32 \le l \le 32$	$-15 \le l \le 15$
reflections collected	19285	25064	34086	128770
independent data	2725	3018	6822	7176
restraints	0	0	33	49
parameters refined	104	152	448	239
abs. struct. param.	-	-	-	–0.004(4)
GooF^a	1.038	1.043	1.024	1.045
R1,^{b,c} wR2^{d,c}	0.0446, 0.1185	0.0427, 0.1025	0.0614, 0.1536	0.0236, 0.0649
R1,^{b,e} wR2^{d,e}	0.0598, 0.1270	0.0697, 0.1093	0.0736, 0.1639	0.0250, 0.0653
largest diff. peak, e·Å^{-3}	0.532	0.642	1.004	0.605
largest diff. hole, e·Å^{-3}	–0.262	–0.596	–0.627	–0.679

^{a}GooF = $\{\Sigma[w(F_o^2 - F_c^2)^2]/(n - p)\}^{½}$, where n= number of reflections and p is the total number of parameters refined; ^{b}R1 = $\Sigma||F_o| - |F_c||/\Sigma|F_o|$; ^{c}R indices for data cut off at I > 2σ(I); ^{d}wR2 = $\{\Sigma[w(F_o^2 - F_c^2)^2]/\Sigma w(F_o^2)^2\}^{1/2}$; $w = 1/[\sigma^2(F_o^2) + (xP)^2 + yP]$, where $P = (F_o^2 + 2F_c^2)/3$; ^{e}R indices for all data.

Table S2. Crystal and refinement data for [Cl_2Pd(tptbz)], [Cl_2Pt(tptbz)], and [Cl_2Pd(tptbz)$PdCl_2$].

compound	[Cl_2Pd(tptbz)]	[Cl_2Pt(tptbz)]	[Cl_2Pt(tptbz)]	[Cl_2Pd(tptbz)$PdCl_2$]
structure code	JPD1108_4_a	JPD1163_0m_a	JPD1121_5	JPD1261_0m_4_a
solvent	none	none	none	2DMI
formula	$C_{18}H_{30}Cl_2PdS_4$	$C_{18}H_{30}Cl_2PtS_4$	$C_{18}H_{30}Cl_2PtS_4$	$C_{28}H_{50}Cl_4N_4O_2Pd_2S_4$
fw	551.96	640.65	640.65	957.56
temperature, K	150	150	150	150
wavelength, Å	0.71073	0.71073	0.71073	0.71073
2θ range, deg.	4.734 - 54.526	4.774 - 58.666	3.998 - 53.090	5.784 - 52.984
crystal system	monoclinic	monoclinic	triclinic	triclinic
space group	$P2_1/c$	$P2_1/c$	*P*-1	*P*-1
a, Å	13.8803(12)	13.8706(8)	9.6258(4)	7.2811(9)
b, Å	9.7851(9)	9.6553(6)	13.8418(6)	10.6186(14)
c, Å	18.3062(16)	18.5310(11)	18.1282(7)	12.9352(18)
α, deg.	90	90	81.7284(12)	75.021(6)
β, deg.	98.921(3)	100.312(3)	87.3855(14)	80.911(6)
γ, deg.	90	90	84.3403(14)	88.730(5)
volume, $Å^3$	2456.3(4)	2441.7(3)	2377.38(17)	953.8(2)
Z	4	4	4	1
density, g/cm^3	1.493	1.743	1.790	1.667
μ, mm^{-1}	1.314	6.309	6.479	1.474
F(000)	1128	1256	1.256	486
crystal size	0.059 x 0.131 x 0.195	0.090 x 0.204 x 0.254	0.025 x 0.124 x 0.156	0.102 x 0.184 x 0.405
color, habit	yellow slat	light yellow plate	colorless plate	yellow bar
limiting indices, *h*	$-17 \le h \le 17$	$-18 \le h \le 18$	$-12 \le h \le 12$	$-8 \le h \le 9$
limiting indices, *k*	$-12 \le k \le 12$	$-13 \le k \le 13$	$-17 \le k \le 17$	$-12 \le k \le 13$
limiting indices, *l*	$-23 \le l \le 23$	$-25 \le l \le 25$	$-22 \le l \le 22$	$0 \le l \le 16$
reflections collected	105892	116883	60207	3351
independent data	5476	6591	60207	3351
restraints	0	0	216	6
parameters refined	234	234	467	211
GooF^a	1.124	1.018	1.006	1.141
R1,^{b,c} wR2^{d,c}	0.0822, 0.2262	0.0453, 0.1137	0.0639, 0.1223	0.0876, 0.2270
R1,^{b,e} wR2^{d,e}	0.1006, 0.2387	0.0618, 0.1269	0.1182, 0.1425	0.1110, 0.2386
largest diff. peak, e·$Å^{-3}$	2.346	3.237	3.097	1.875
largest diff. hole, e·$Å^{-3}$	–0.721	–1.201	–1.785	–1.825

^{a}GooF = $\{\Sigma[w(F_o^2 - F_c^2)^2]/(n - p)\}^{1/2}$, where *n*= number of reflections and *p* is the total number of parameters refined; ^{b}R1 = $\Sigma||F_o| - |F_c||/\Sigma|F_o|$; ^{c}R indices for data cut off at I > 2σ(I); ^{d}wR2 = $\{\Sigma[w(F_o^2 - F_c^2)^2]/\Sigma w(F_o^2)^2\}^{1/2}$; $w = 1/[\sigma^2(F_o^2) + (xP)^2 + yP]$, where $P = (F_o^2 + 2F_c^2)/3$; ^{e}R indices for all data.

Table S3. Crystal and Refinement Data for [(mnt)Pd(tptbz)], [(mnt)Pt(tptbz)], [(pdt)Pt(tptbz)],

compound	[(mnt)Pd(tptbz)]	[(mnt)Pt(tptbz)]	[(pdt)Pt(tptbz)]
structure code	JPD1122_0m_a	JPD1179_0m_a	JPD1138_5
solvent	none	none	none
formula	$C_{22}H_{30}N_2PdS_6$	$C_{22}H_{30}N_2PtS_6$	$C_{32}H_{40}PtS_6$
fw	621.24	709.93	812.09
temperature, K	150	160	150
wavelength, Å	0.71073	0.71073	0.71073
2θ range, deg.	4.562 – 63.908	4.552 – 82.502	1.840 – 61.276
crystal system	monoclinic	monoclinic	triclinic
space group	$P2_1/c$	$P2_1/c$	*P*-1
a, Å	5.7962(1)	5.7765(1)	15.9373(8)
b, Å	19.2210(5)	19.2629(4)	16.3234(8)
c, Å	24.1842(6)	24.2681(5)	22.6186(12)
α, deg.	90	90	85.242(2)
β, deg.	94.386(1)	94.555(1)	78.027(2)
γ, deg.	90	90	62.321(2)
volume, $Å^3$	2686.44(11)	2691.83(9)	5096.9(5)
Z	4	4	6
density, g/cm^3	1.536	1.752	1.587
μ, mm^{-1}	1.171	5.692	4.519
F(000)	1272	1400	2436
crystal size	0056 x 0.256 x 0.369	0.063 x0.074 x 0.307	0.074 x 0.178 x 0.297
color, habit	light green plate	pale yellow column	thick yellow plate
limiting indices, *h*	$-8 \le h \le 8$	$-10 \le h \le 10$	$-22 \le h \le 22$
limiting indices, *k*	$-28 \le k \le 28$	$-35 \le k \le 35$	$-23 \le k \le 23$
limiting indices, *l*	$-35 \le l \le 35$	$-44 \le l \le 45$	$0 \le l \le 32$
reflections collected	177124	213713	48672
independent data	9273	18001	48672
restraints	10	4	1
parameters refined	311	300	1082
GooF[a]	1.175	1.126	1.095
R1,[b,c] wR2[d,c]	0.0255, 0.0536	0.0415, 0.0784	0.0381, 0.0791
R1,[b,e] wR2[d,e]	0.0282, 0.0547	0.0559, 0.0828	0.0602, 0.0892
largest diff. peak, e·$Å^{-3}$	0.683	5.421	2.203
largest diff. hole, e·$Å^{-3}$	–0.944	–2.856	–1.634

[a]GooF = $\{\Sigma[w(F_o^2 - F_c^2)^2]/(n - p)\}^{½}$, where *n*= number of reflections and *p* is the total number of parameters refined; [b]R1 = $\Sigma||F_o| - |F_c||/\Sigma|F_o|$; [c]R indices for data cut off at I > 2σ(I); [d]wR2 = $\{\Sigma[w(F_o^2 - F_c^2)^2]/\Sigma w(F_o^2)^2\}^{1/2}$; $w = 1/[\sigma^2(F_o^2) + (xP)^2 + yP]$, where $P = (F_o^2 + 2F_c^2)/3$; [e]R indices for all data.

Table S4. Crystal and Refinement Data for $[Cu(tptbz)]_n[PF_6]_n \cdot DMF_n$.

compound	$[Cu(tptbz)]_n[PF_6]_n \cdot DMF_n$				
structure code	JPD1164_0m_a				
solvent	DMF				
formula	$C_{21}H_{33}CuF_6NOPS_4$				
fw	652.33				
temperature, K	150				
wavelength, Å	0.71073				
2θ range, deg.	2.306 - 66.588				
crystal system	monoclinic				
space group	*C*2/*m*				
a, Å	19.133(3)				
b, Å	17.253(2)				
c, Å	18.461(3)				
α, deg.	90				
β, deg.	107.016(4)				
γ, deg.	90				
volume, $Å^3$	5827.3(14)				
Z	8				
density, g/cm^3	1.487				
μ, mm^{-1}	1.145				
F(000)	2688				
crystal size	0.084 x 0.205 x 0.228				
color, habit	colorless tablet				
limiting indices, *h*	$-29 \le h \le 29$				
limiting indices, *k*	$-26 \le k \le 26$				
limiting indices, *l*	$-28 \le l \le 28$				
reflections collected	151087				
independent data	11481				
restraints	13				
parameters refined	364				
GooF[a]	1.035				
R1,[b,c] wR2[d,c]	0.0423, 0.1193				
R1,[b,e] wR2[d,e]	0.0588, 0.1336				
largest diff. peak, e·$Å^{-3}$	1.544				
largest diff. hole, e·$Å^{-3}$	–1.091				

[a]GooF = $\{\Sigma[w(F_o^2 - F_c^2)^2]/(n-p)\}^{1/2}$, where *n*= number of reflections and *p* is the total number of parameters refined; [b]R1 = $\Sigma||F_o| - |F_c||/\Sigma|F_o|$; [c]R indices for data cut off at I > 2σ(I); [d]wR2 = $\{\Sigma[w(F_o^2 - F_c^2)^2]/\Sigma w(F_o^2)^2\}^{1/2}$; $w = 1/[\sigma^2(F_o^2) + (xP)^2 + yP]$, where $P = (F_o^2 + 2F_c^2)/3$; [e]R indices for all data.

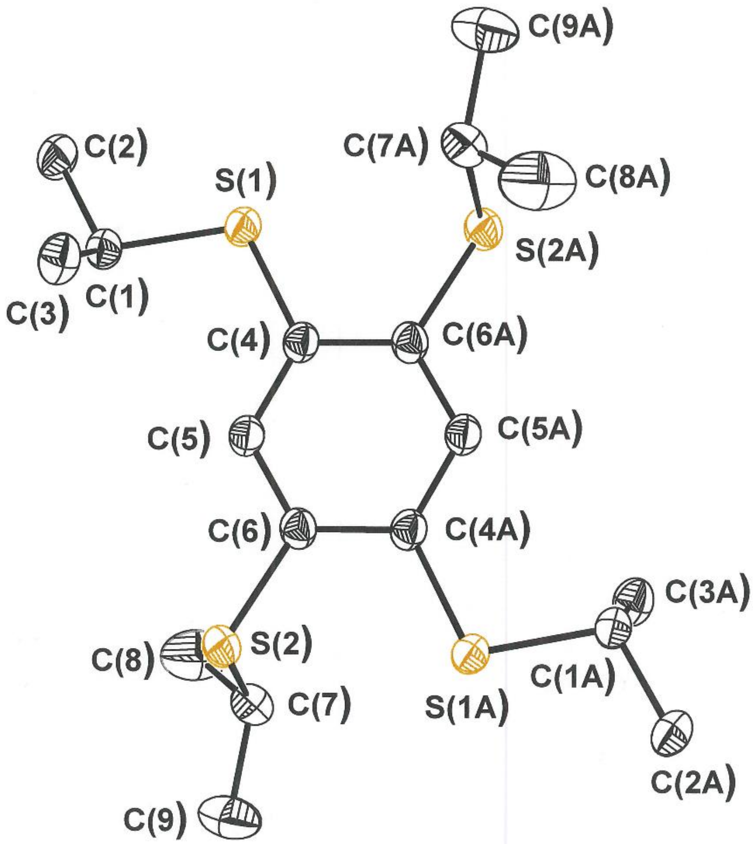

Figure S1. Thermal ellipsoid plot (50%) of 1,2,4,5-tetrakis(isopropylthio)benzene (tptbz) with atom labeling. All H atoms are omitted for clarity. The center of the arene ring is coincident with an inversion center.

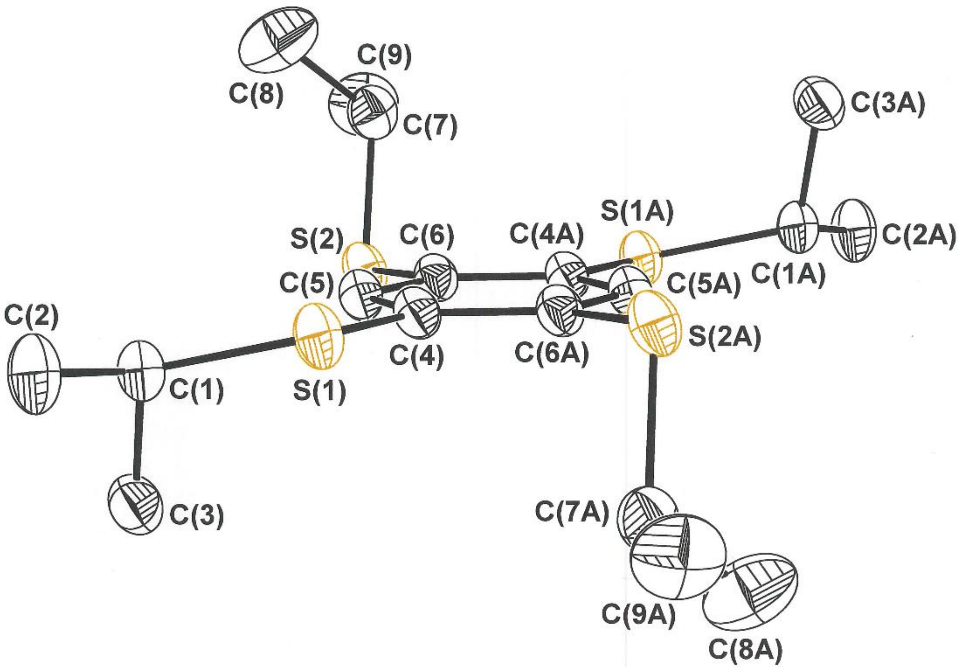

Figure S2. Thermal ellipsoid plot (50%) of 1,2,4,5-tetrakis(isopropylthio)benzene with atom labeling (tptbz). This view is orthogonal to that in **Figure S1**. All H atoms are omitted for clarity. The center of the arene ring is coincident with an inversion center.

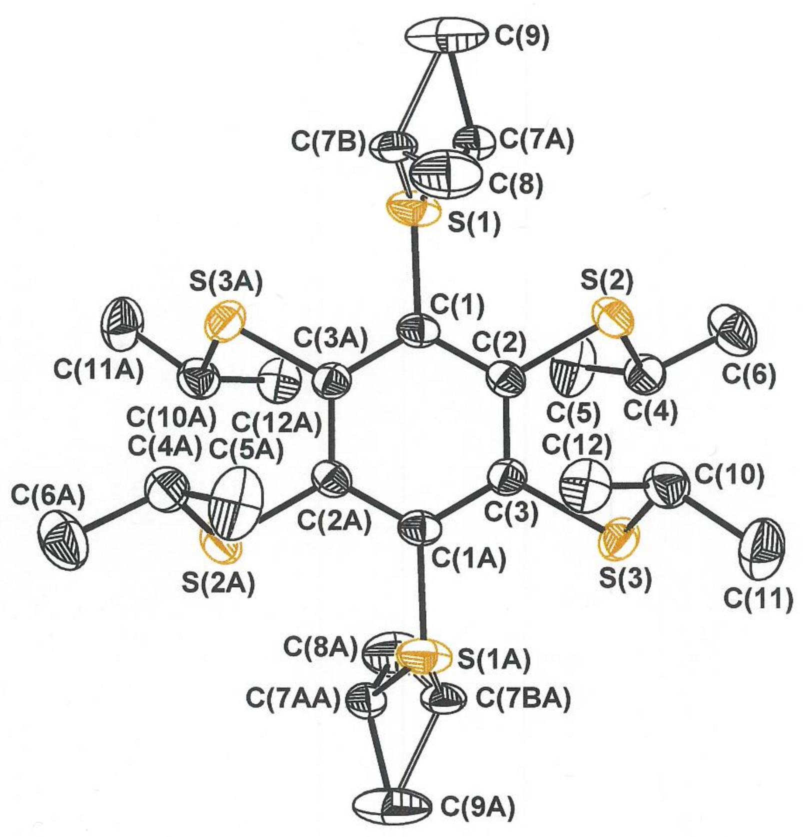

Figure S3. Thermal ellipsoid plot (50%) of 1,2,3,4,5,6-hexakis(isopropylthio)benzene with atom labeling. The center of the arene ring is coincident with an inversion center. The C(7) methine-type carbon atom is disordered over two positions and refined as a best-fit 76:24 distribution between them. All H atoms are omitted for clarity.

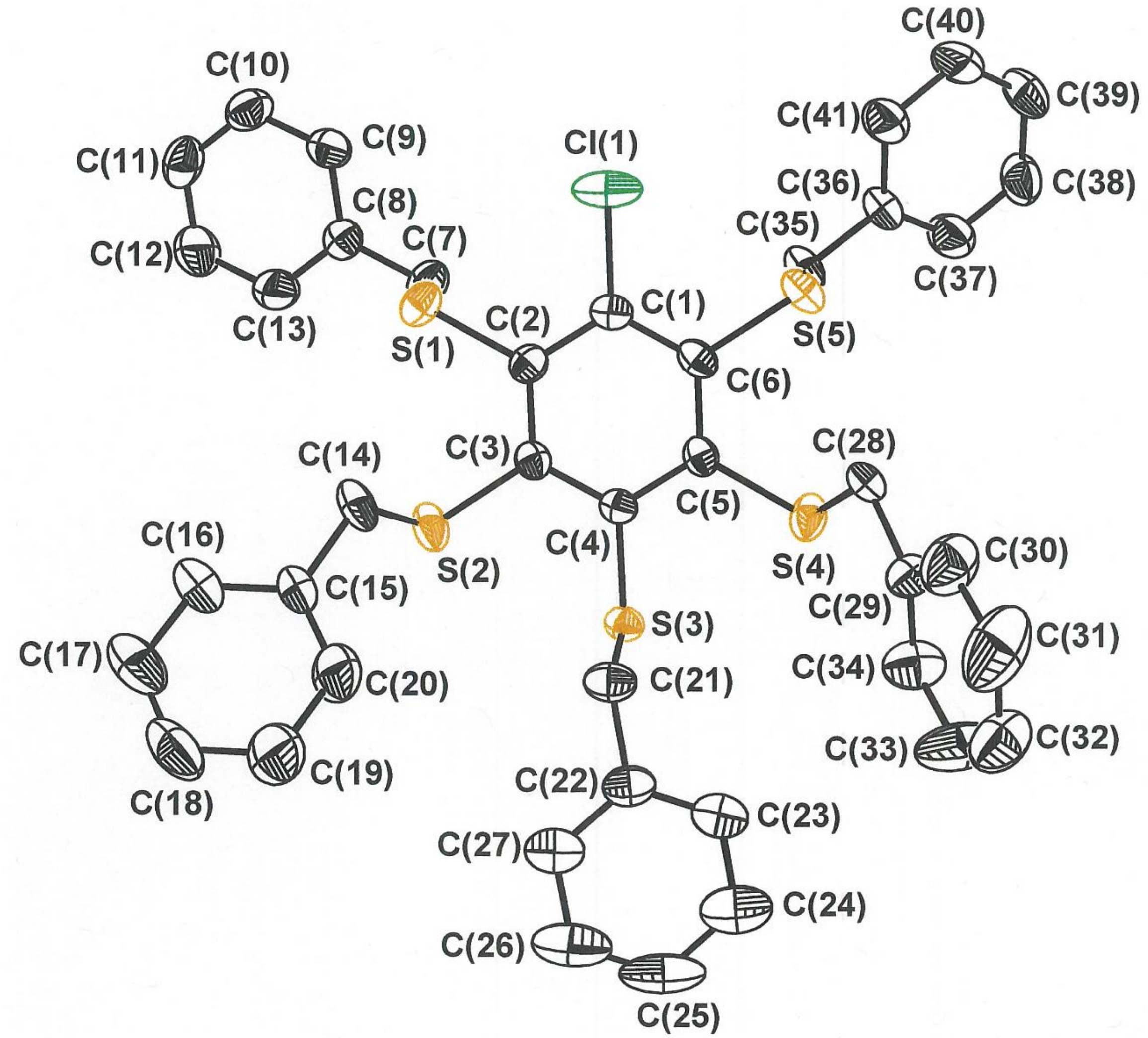

Figure S4. Thermal ellipsoid plot (50%) of [$C_6Cl(SCH_2C_6H_5)_5$] with atom labeling. All H atoms are omitted for clarity. The four benzyl groups marked by S(2), S(3), S(4) and S(5) are disordered over two positions, one of which is shown.

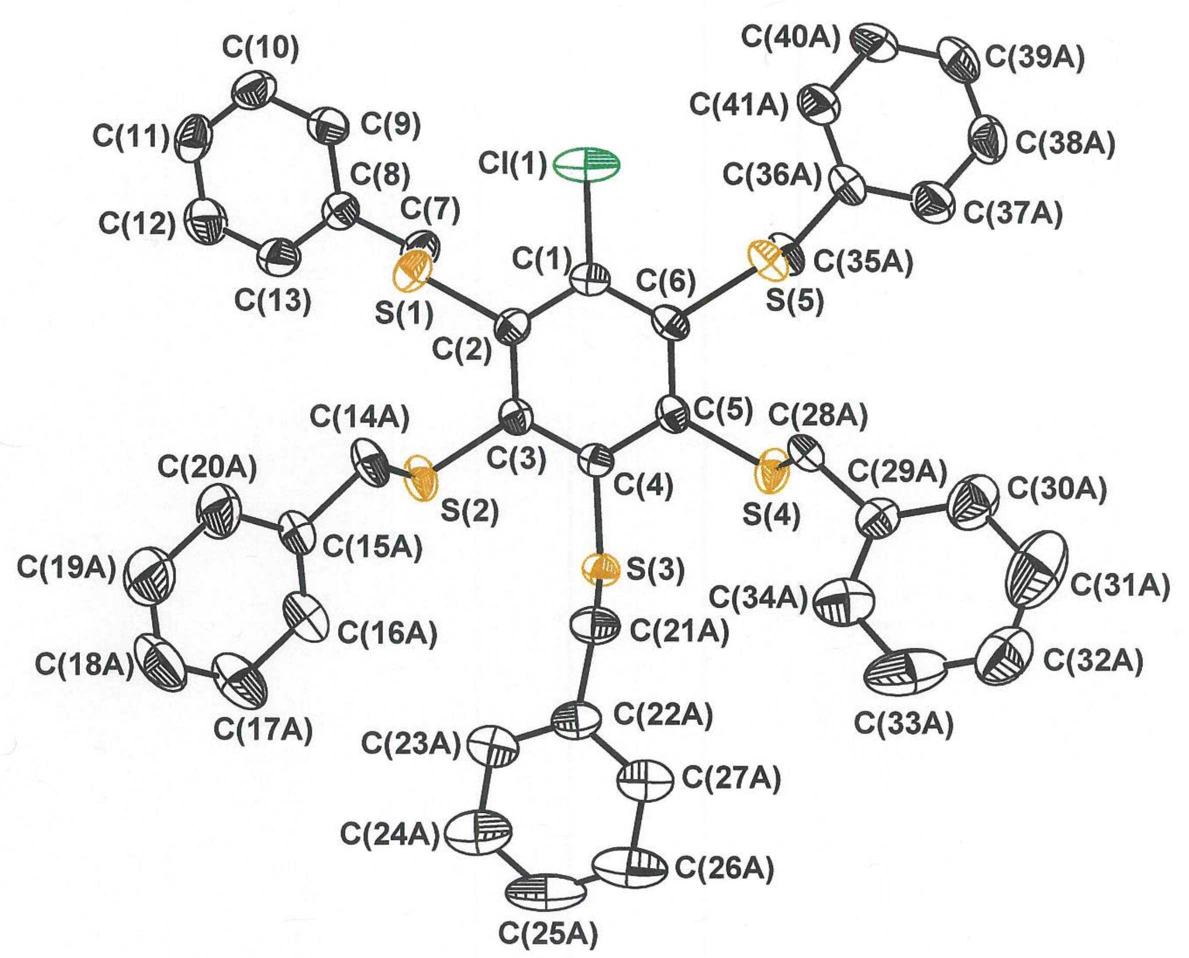

Figure S5. Thermal ellipsoid plot (50%) of [$C_6Cl(SCH_2C_6H_5)_5$] with atom labeling. All H atoms are omitted for clarity. Here, the second position of the four disordered benzyl groups marked by S(2), S(3), S(4) and S(5) are shown.

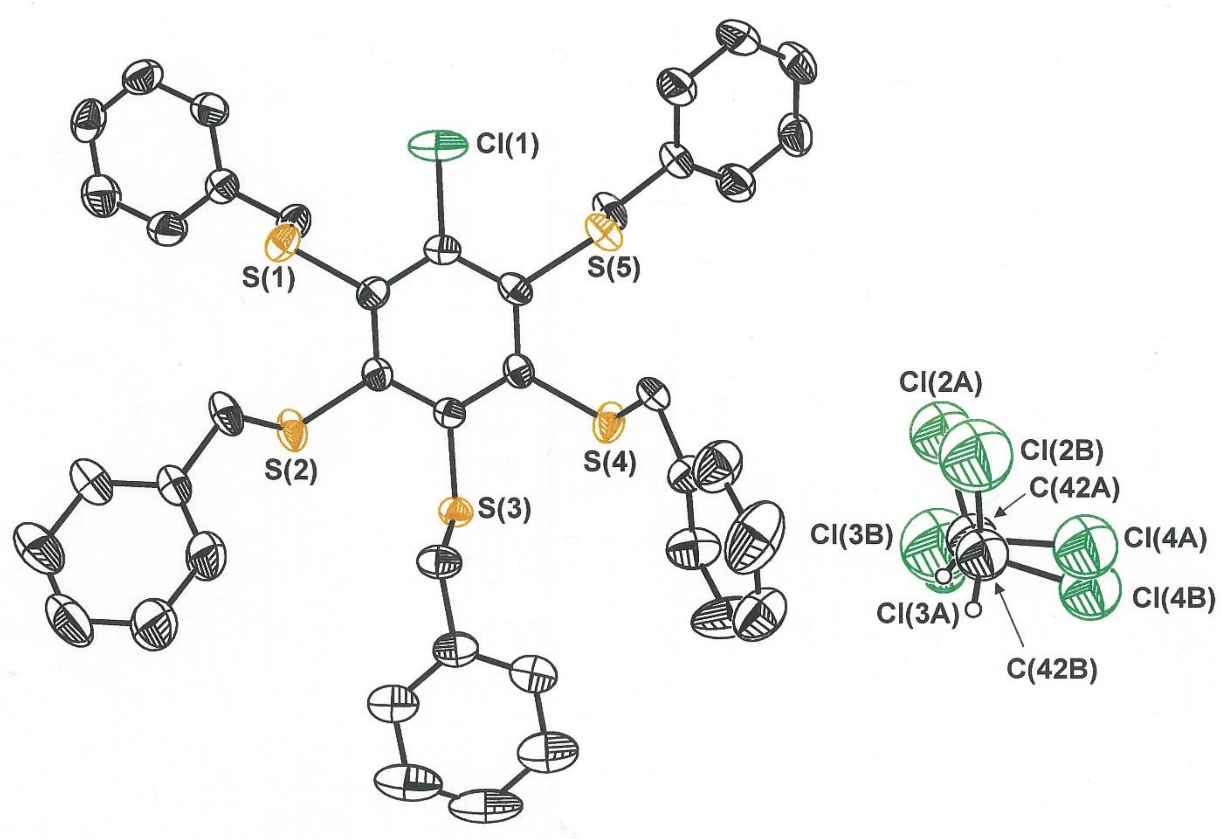

Figure S6. Thermal ellipsoid plot (50%) of [$C_6Cl(SCH_2C_6H_5)_5$] with interstitial $^1/_4CHCl_3$ molecule. All H atoms are omitted for clarity.

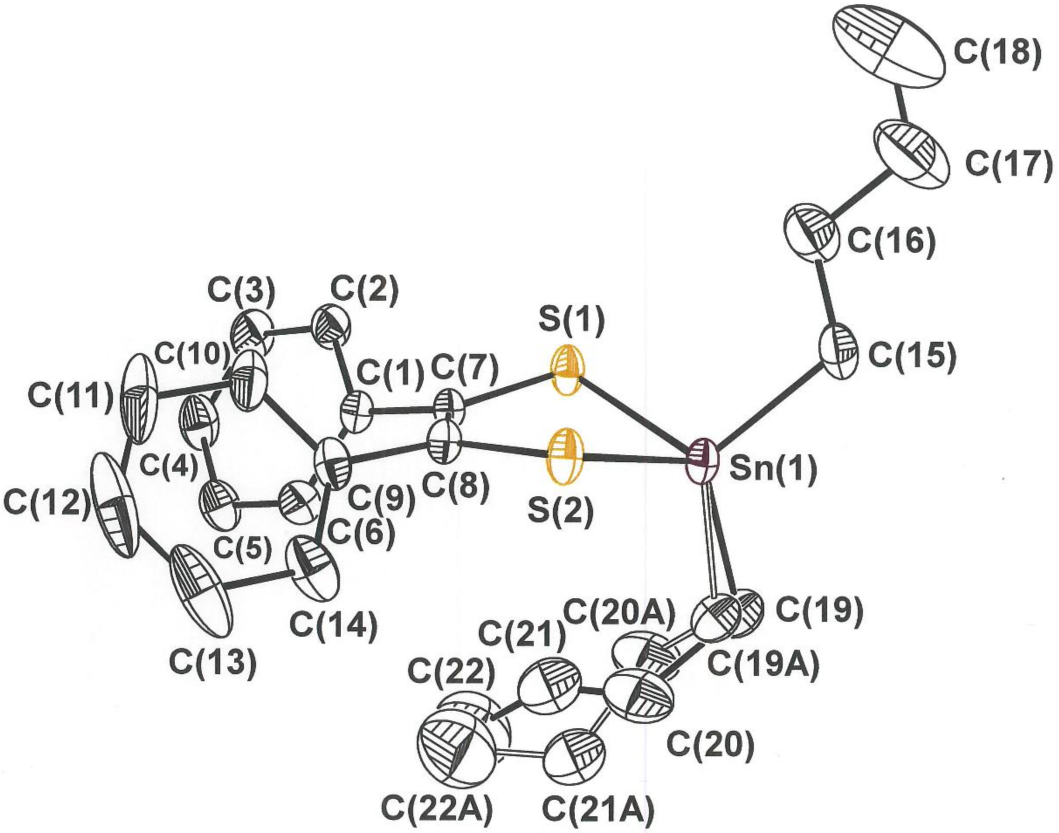

Figure S7. Thermal ellipsoid plot (50%) of [($Ph_2C_2S_2$)Sn^nBu_2] with atom labeling. All H atoms are omitted for clarity. The butyl group defined by C(19)-C(22) is disordered over two positions, the distribution between which has been modeled as a variable best-fit by the refinement software.

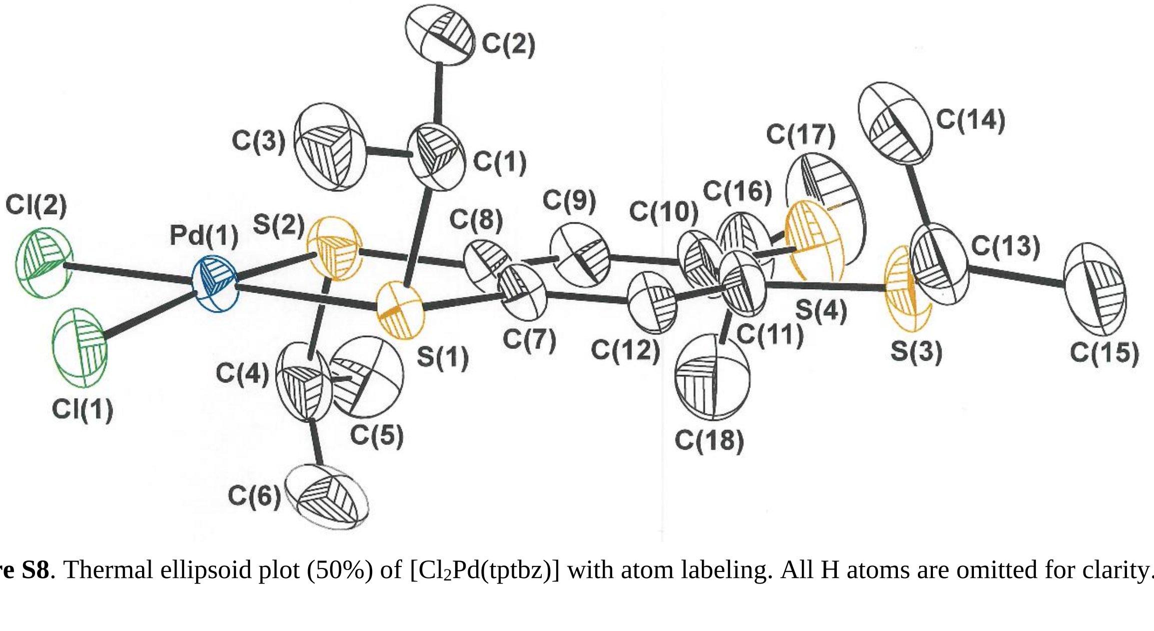

Figure S8. Thermal ellipsoid plot (50%) of [Cl_2Pd(tptbz)] with atom labeling. All H atoms are omitted for clarity.

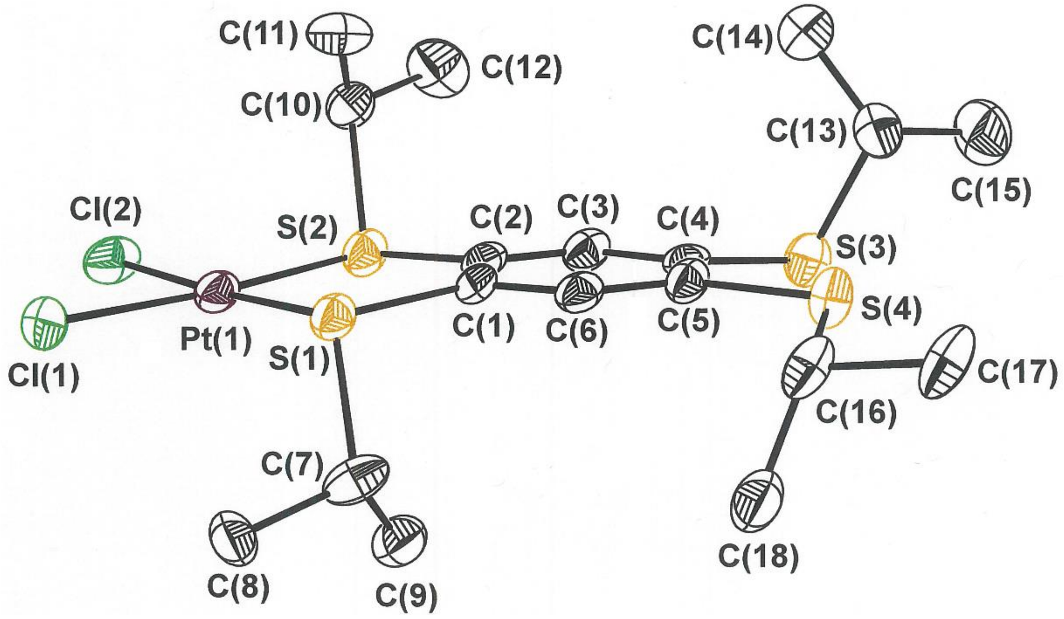

Figure S9. Thermal ellipsoid plot (50%) of molecule 1 of [Cl_2Pt(tptbz)], triclinic polymorph, with atom labeling. All H atoms are omitted for clarity.

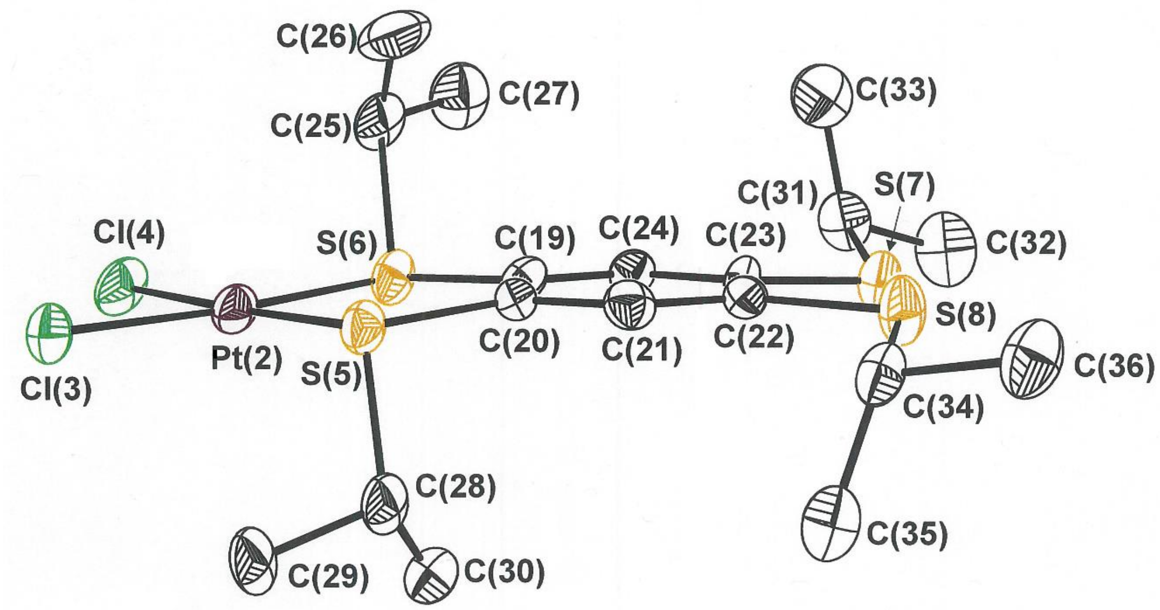

Figure S10. Thermal ellipsoid plot (50%) of molecule 2 of [Cl_2Pt(tptbz)], triclinic polymorph, with atom labeling. All H atoms are omitted for clarity.

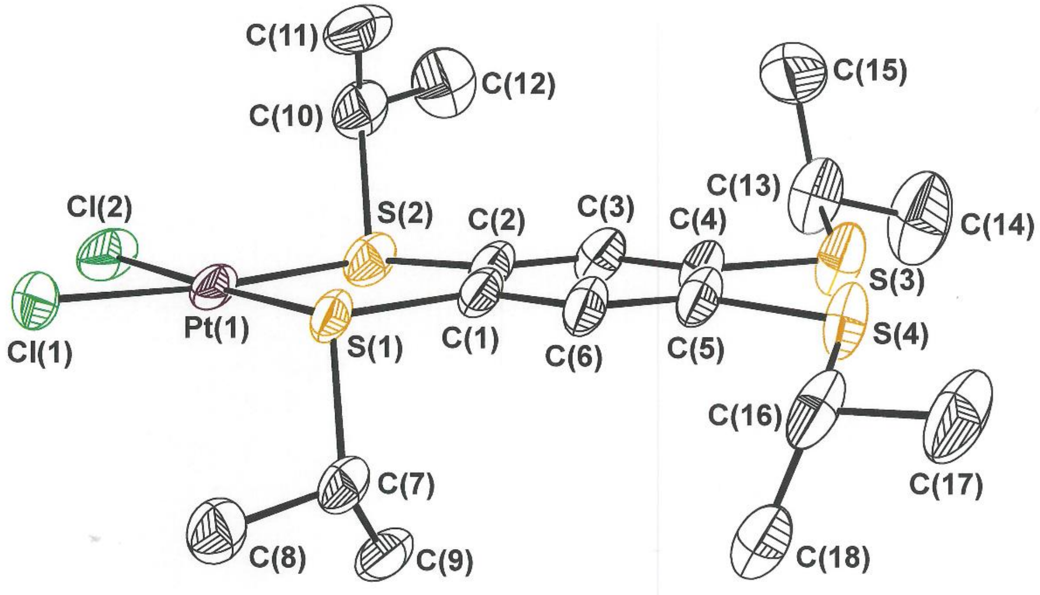

Figure S11. Thermal ellipsoid plot (30%) of [Cl_2Pt(tptbz)], monoclinic polymorph, with atom labeling. All H atoms are omitted for clarity.

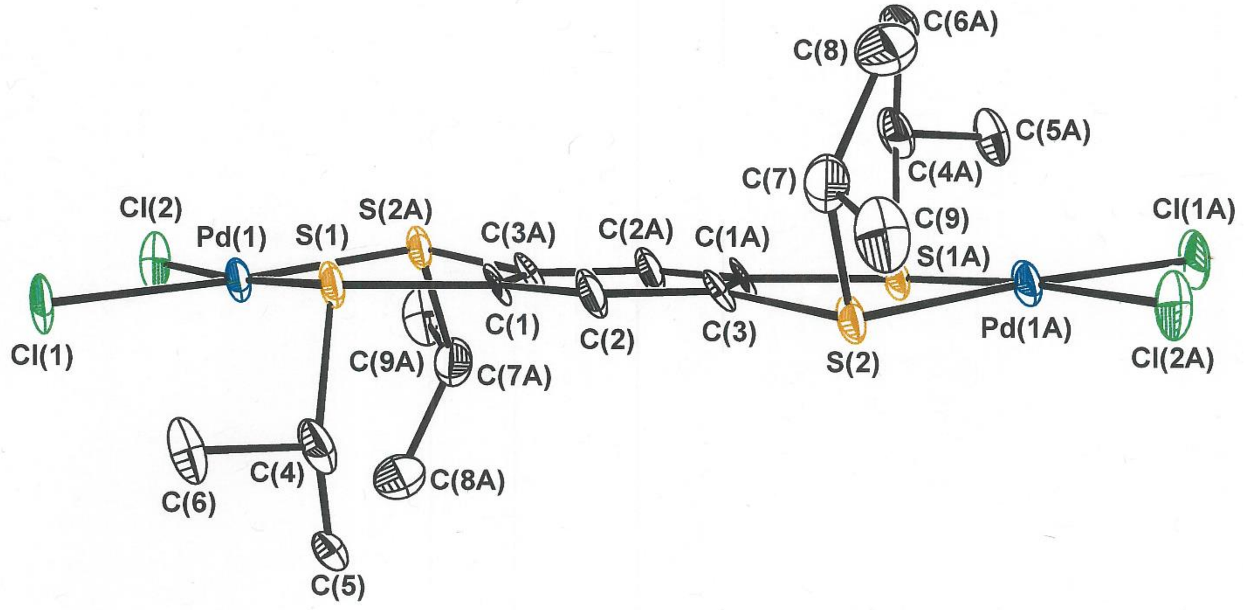

Figure S12. Thermal ellipsoid plot (50%) of [Cl_2Pd(tptbz)$PdCl_2$] with atom labeling. All H atoms are omitted for clarity.

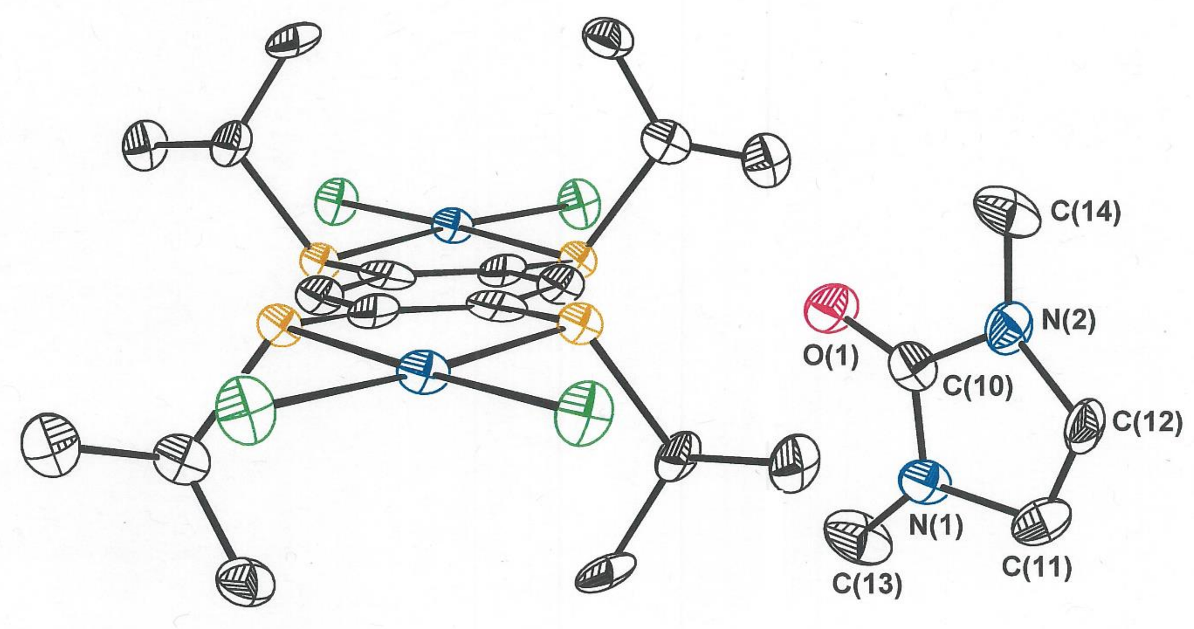

Figure S13. Thermal ellipsoid plot (50%) of [Cl_2Pd(tptbz)$PdCl_2$]·2DMI with atom labeling for the unique 1,3-dimethyl-2-imidazolidinone (DMI) molecule. The symmetry-related DMI molecule is not shown. All H atoms are omitted for clarity.

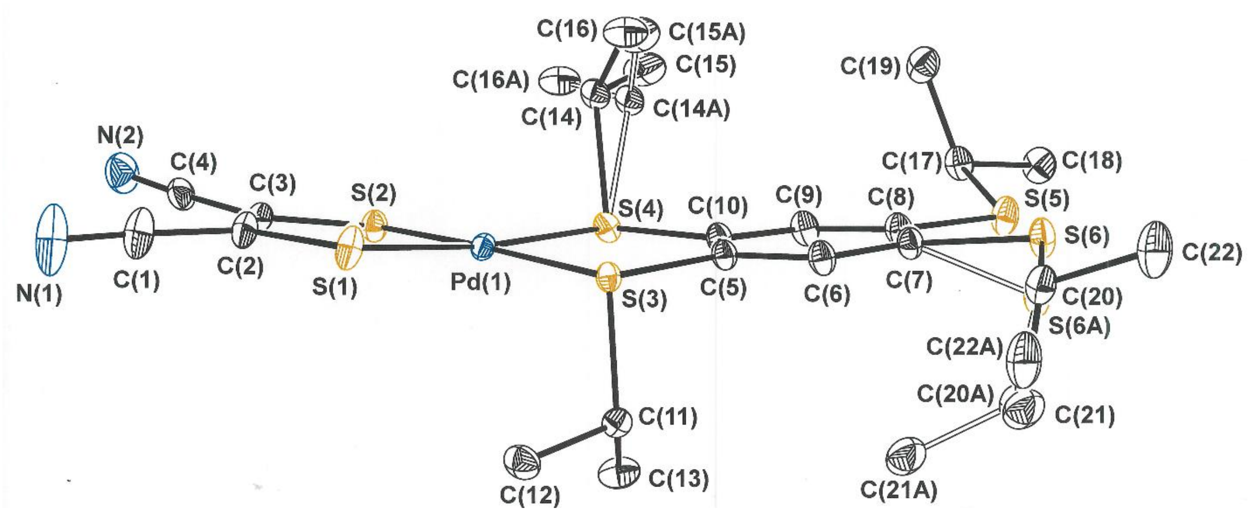

Figure S14. Thermal ellipsoid plot (50%) of [((NC)$_2$C$_2$S$_2$)Pd(tptbz)] with atom labeling. All H atoms are omitted for clarity. The isopropyl group defined by C(14)-C(160 and the isopropylthio group defined by S(6)-C(22) are both disordered over two positions, the distribution between which has been modeled as a variable best-fit by the refinement software.

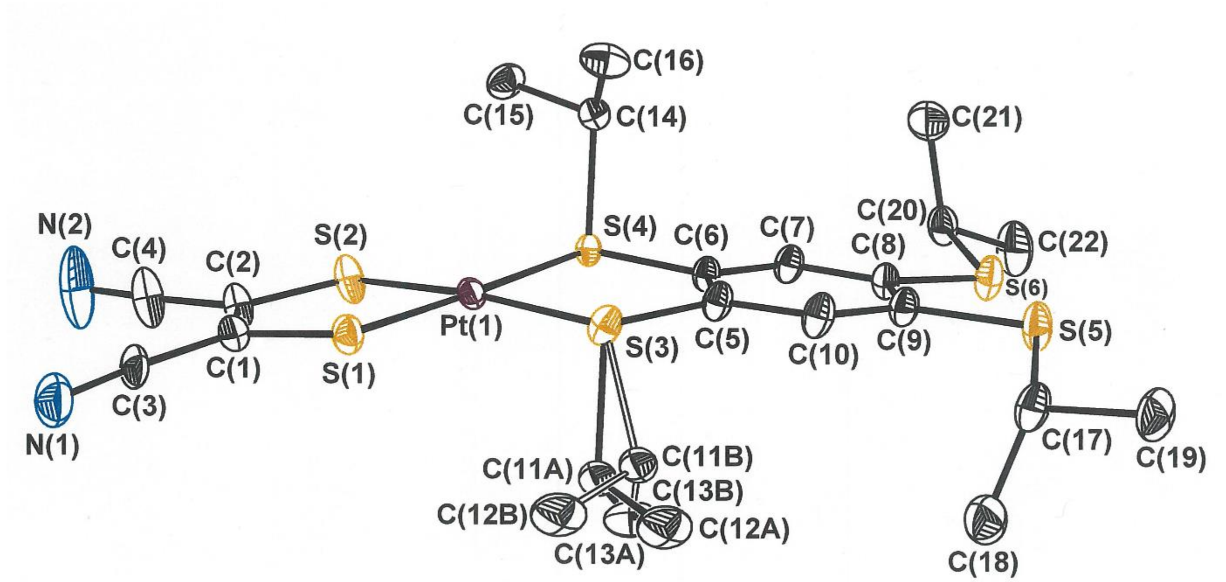

Figure S15. Thermal ellipsoid plot (50%) of [((NC)$_2$C$_2$S$_2$)Pt(tptbz)] with atom labeling. All H atoms are omitted for clarity. The C(11)-C(13) isopropyl group is disordered and refined as a distribution over two positions.

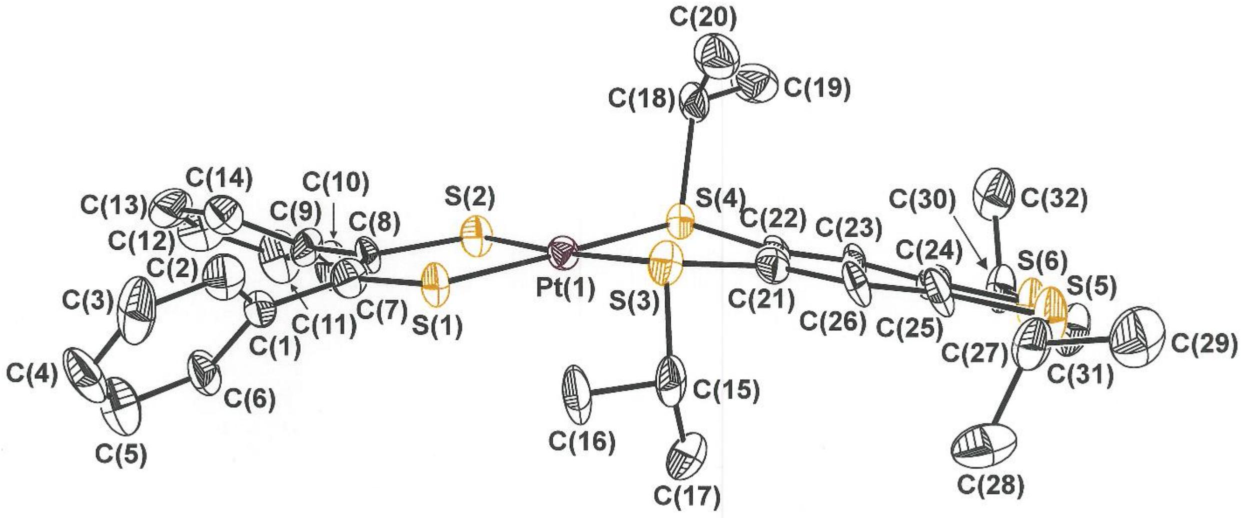

Figure S16. Thermal ellipsoid plot (50%) of [$(Ph_2C_2S_2)Pt(tptbz)$] with atom labeling, molecule 1 of 3 in the asymmetric unit. All H atoms are omitted for clarity.

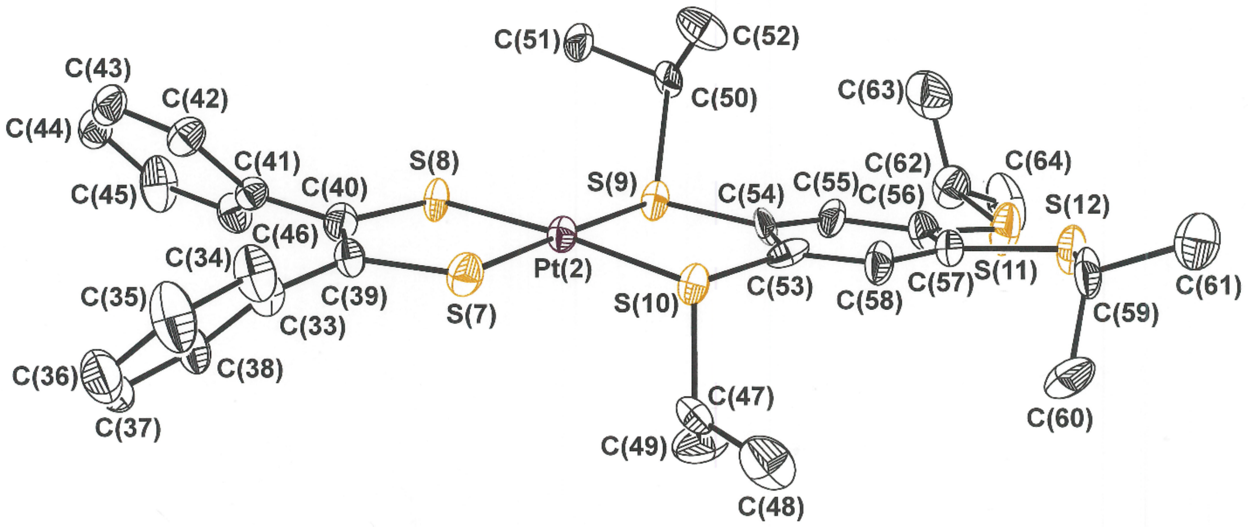

Figure S17. Thermal ellipsoid plot (50%) of [$(Ph_2C_2S_2)Pt(tptbz)$] with atom labeling, molecule 2 of 3 in the asymmetric unit. All H atoms are omitted for clarity.

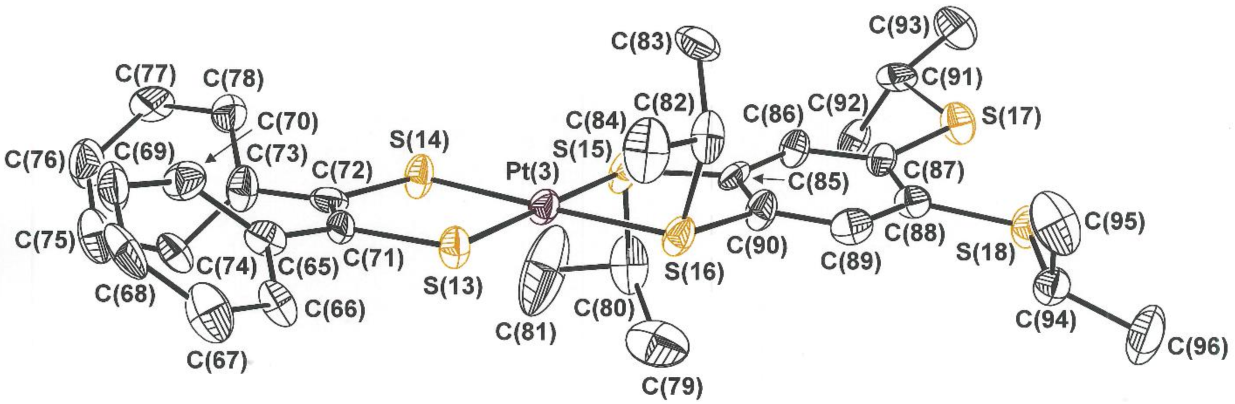

Figure S18. Thermal ellipsoid plot (50%) of [$(Ph_2C_2S_2)$Pt(tptbz)] with atom labeling, molecule 3 of 3 in the asymmetric unit. All H atoms are omitted for clarity.

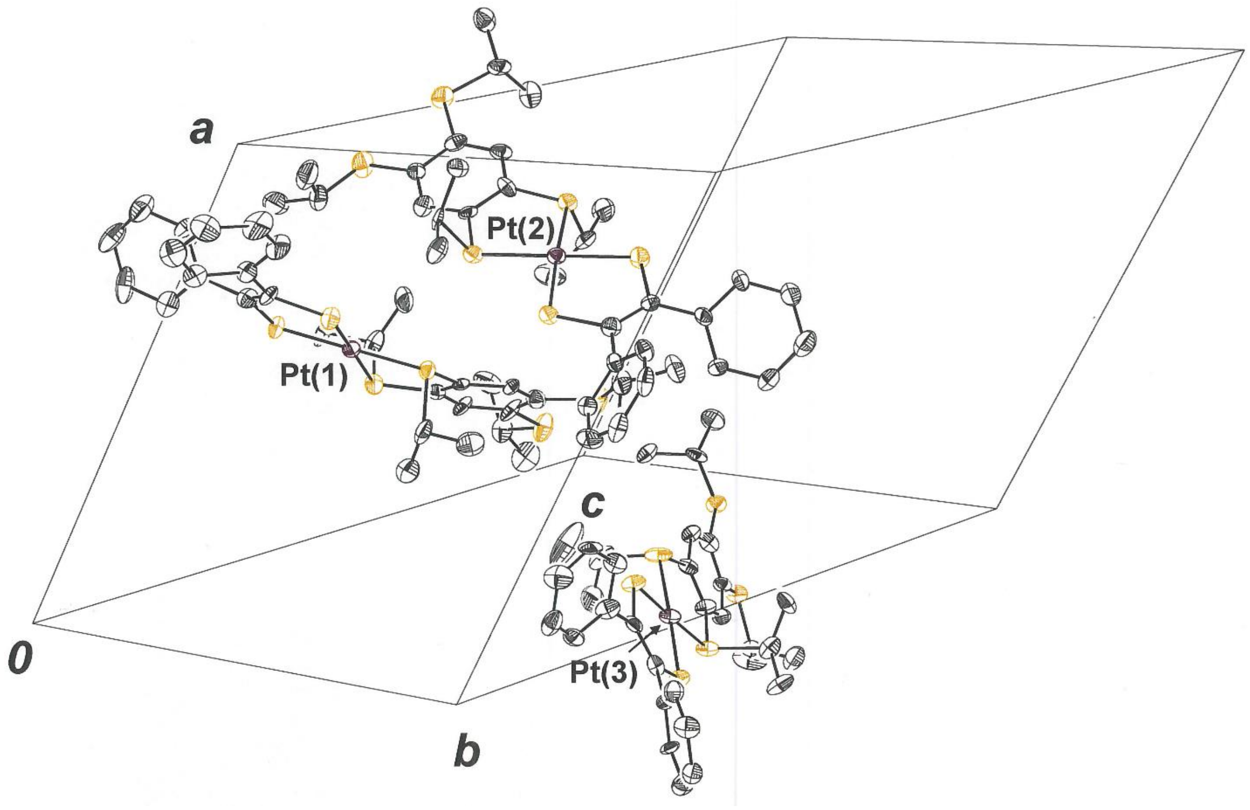

Figure S19. Packing arrangement of the 3 independent molecules of [$(Ph_2C_2S_2)$Pt(tptbz)] in the asymmetric unit of the triclinic unit cell, which provides for the somewhat unusual value of 6 for *Z*. All H atoms are omitted for clarity.

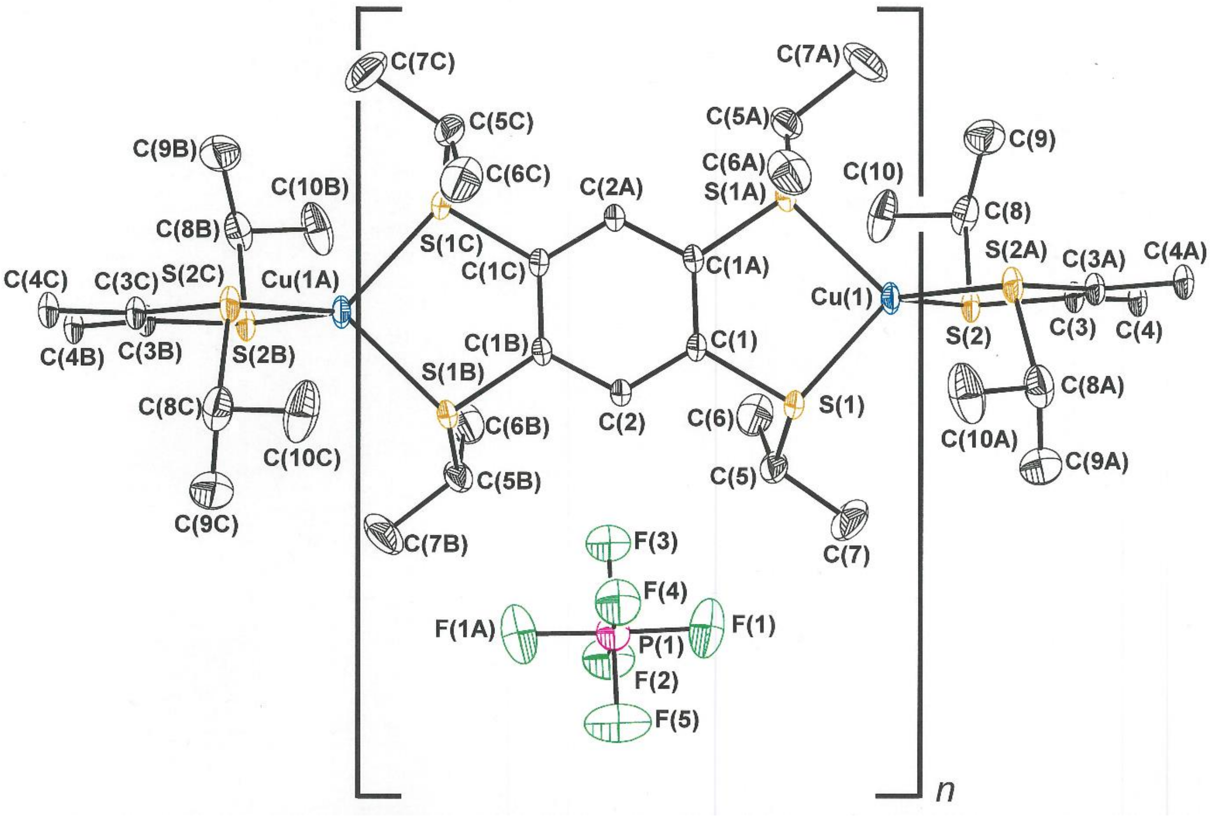

Figure S20. Atom labeling for coordination polymer strand 1 of [Cu(tptbz)][PF_6]. The ellipsoid plot is presented at the 50% level, and all H atoms are omitted for clarity.

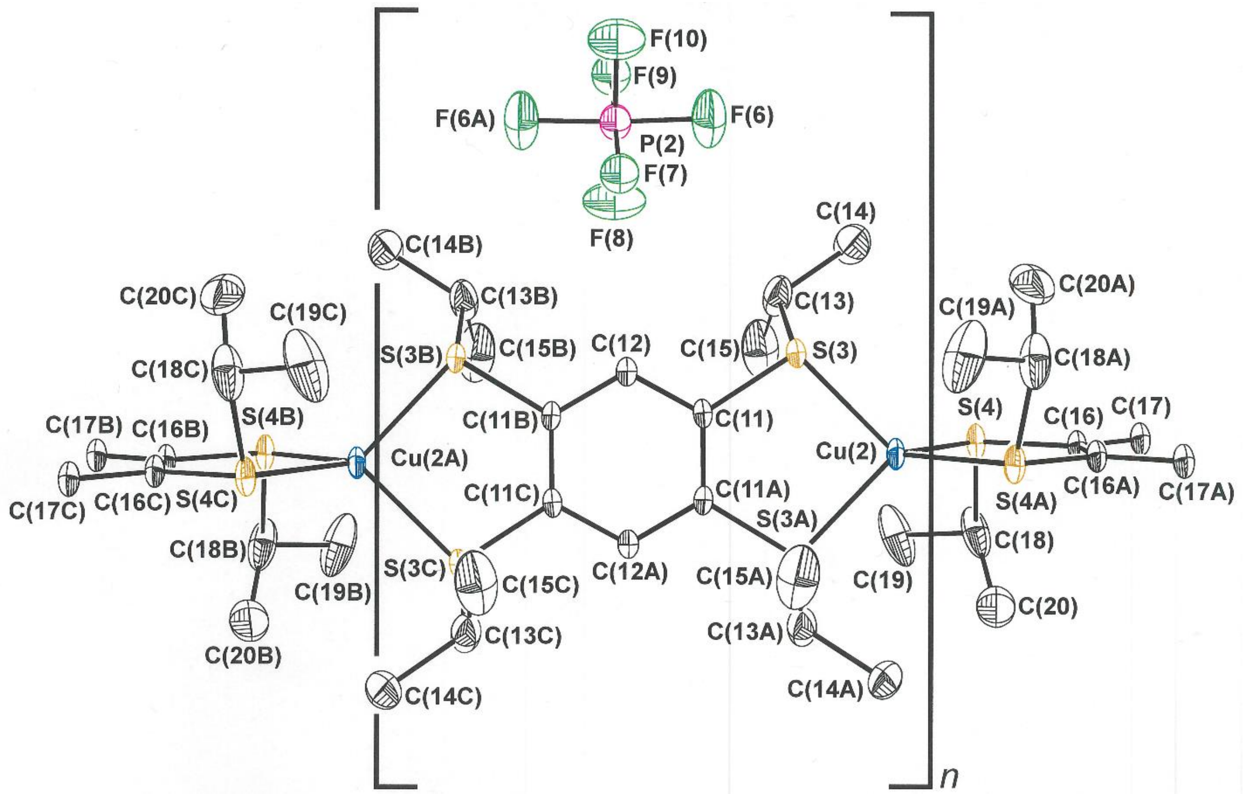

Figure S21. Atom labeling for coordination polymer strand 2 of [Cu(tptbz)][PF_6]. The ellipsoid plot is presented at the 50% level, and all H atoms are omitted for clarity.

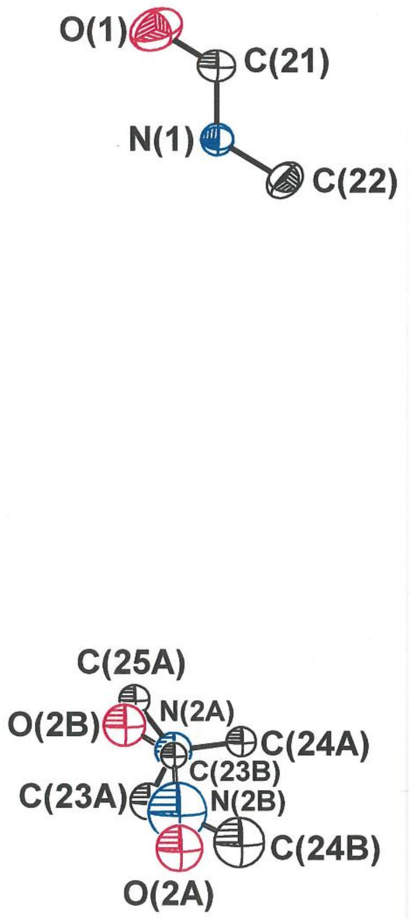

Figure S22. Atom labeling for interstitial *N*,*N*-dimethylformamide in the structure of $[[Cu(tptbz)][PF_6]\cdot DMF]_n$. The ellipsoid plot is presented at the 30% level, and all H atoms are omitted for clarity. Both molecules occur on a special position, which imposes disorder.

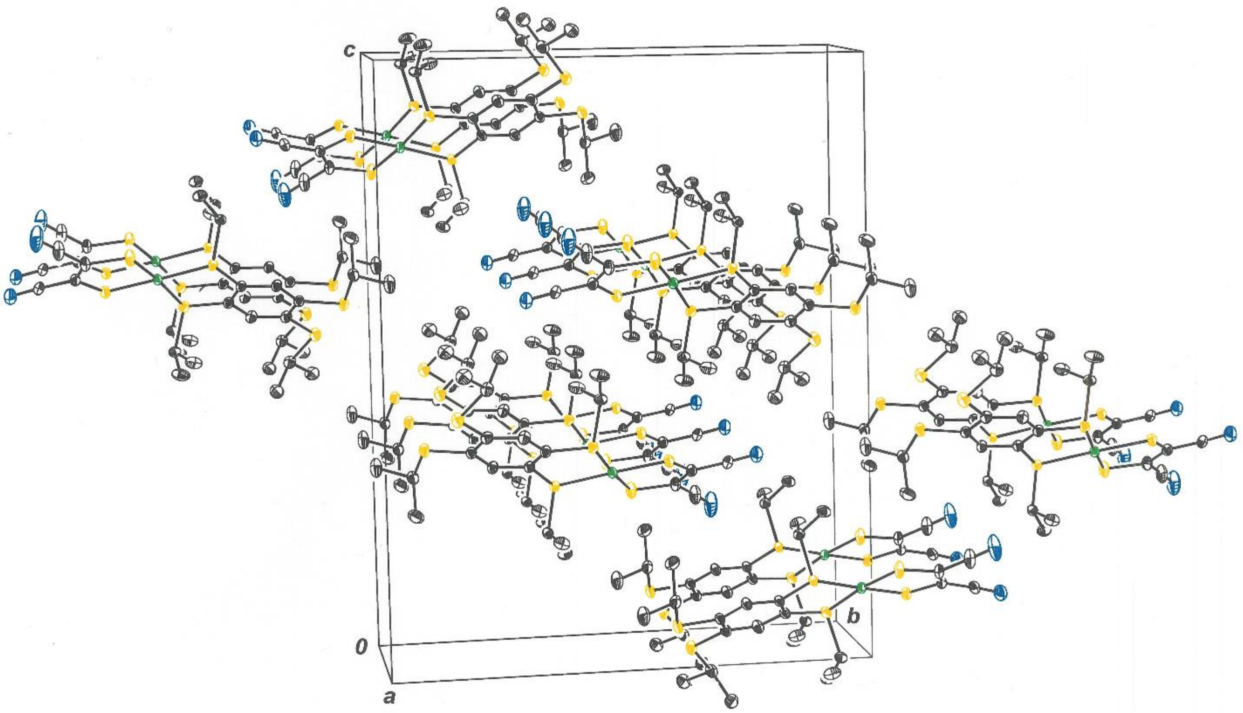

Figure S23. Crystal packing diagram for **5** showing columnar stacks along the approximate direction of the *a* axis. All H atoms are omitted for clarity. Thermal ellipsoids are depicted at the 50% probability level.

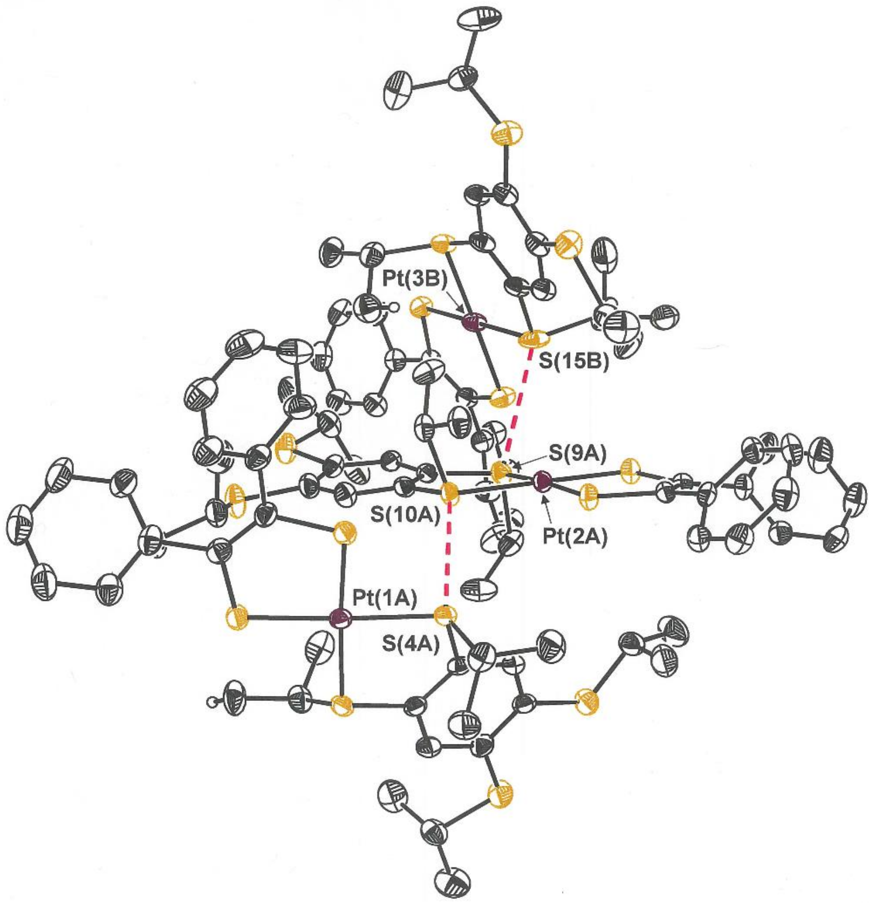

Figure S24. View of the relative arrangement of molecules of **8** in the asymmetric unit of the cell, which includes three independent molecules. Intermolecular S···S close contacts are illustrated as heavy red dashed lines. The S(4A)···S(10A) distance is 3.414 Å, and the S(9A)···S(15B) distance is 3.508 Å. These S···S contacts likely play a role in the packing arrangement of molecules in the cell.

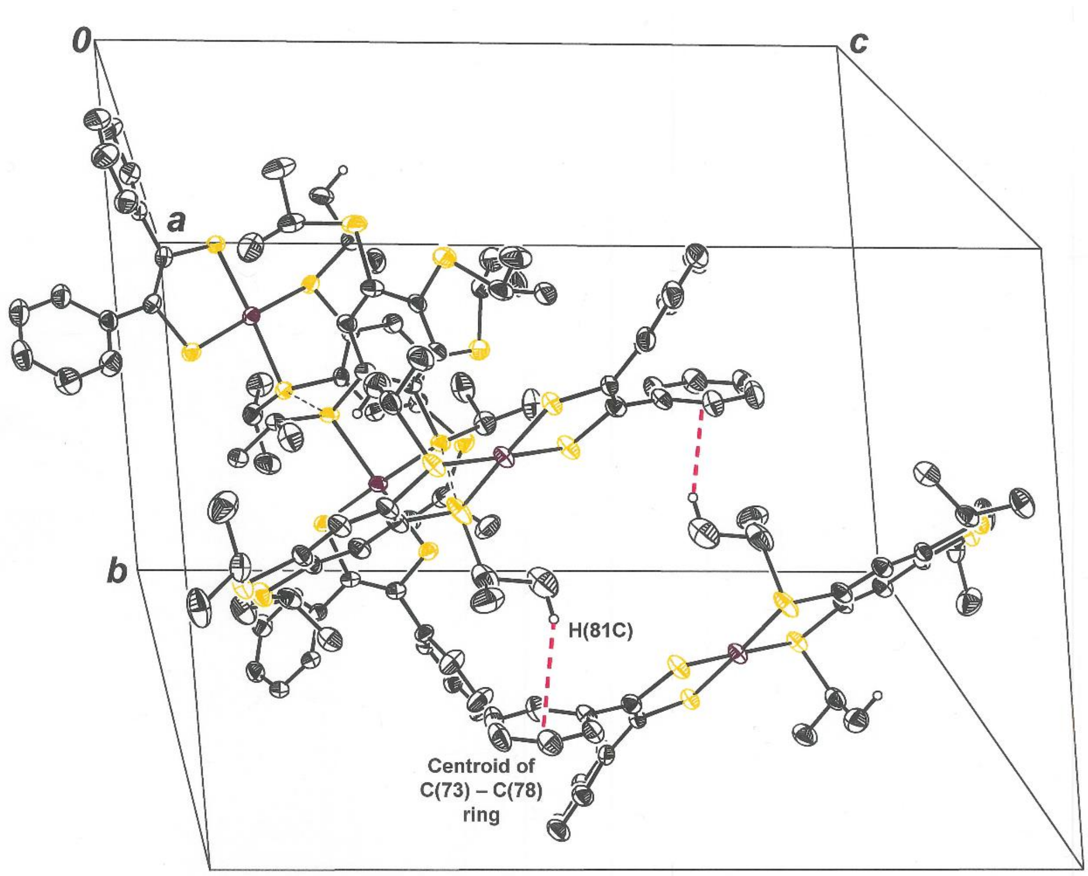

Figure S25. View of the three molecules of **8** in the asymmetric unit of the cell (top left), and one additional molecule of **8** which appears to form a pair of intermolecular C–H···arene$_{centroid}$ hydrogen bonds, shown in heavy red dashed lines, with its inversion-related partner. The H(81C)···C(73)-C(78)$_{centroid}$ distance is 2.80 Å.

GALBRAITH LABORATORIES, INC.

Analytical Request Form

ISO 17025 Certificate #2777.01

ACCREDITED

Please fill out all fields. For assistance, contact us at 1-877-449-8797 or labinfo@galbraith.com

Contact Information		Payment Information	
Name	James P. Donahue	Company	Dept. of Chemistry, Tulane University
Company	Department of Chemistry, Tulane University	Bill to Address	6400 Freret Street
Address	6400 Freret Street	Address (2)	Stern Hall, Room 2015
Address (2)	Stern Hall, Room 2015	City, State/Province	New Orleans, Louisiana
City, State/Province	New Orleans, Louisiana	Zip/Postal Code	70118
Zip/Postal Code	70118	Country	United States of America
Country	United States of America	Phone	504-862-3590
Phone	504-862-3562	PO Number	
Quote Number		Email (CC Receipt)	
Report Results to (list emails)	donahue@tulane.edu	Credit Cardholder Name	Jessica Stephenson
		Credit Card Number	
		Credit Card CVV Code	
Information is only released to the contact above. If others are permitted to receive information, list their names here.		Credit Card Expiration Date	11/22
		Credit Card Type	(●) Visa ( ) MC ( ) Amex

Turnaround Time

- (●) STANDARD - Approximately 10 business days
- ( ) RUSH - 5 business days (100% surcharge)
- ( ) PRIORITY - 2 business days (200% surcharge)

Regulatory Information

Regulatory services incur a 75% surcharge

- (●) No Regulations
- ( ) GMP 21 CFR 210/211 21 CFR 820
- ( ) FIFRA 40 CFR 160
- ( ) GLP 21 CFR 58
- ( ) TSCA 40 CFR 792
- ( ) NRC
- ( ) RCRA

Special Handling

- [ ] Dry prior to analysis (provide conditions below) ($24 per sample) ________ Hours ________°C Vacuum ( ) Yes ( ) No
- [ ] Grind ($40 per sample, subject to rush surcharge)
- [ ] Cold Storage (2 to 8°C) ($11 per sample or $43 per 5+ samples)
- [ ] Freezer Storage (-18 to -25°C) ($11 per sample or $43 per 5+ samples)
- [ ] Freezer Storage (-70°C) ($11 per sample or $43 per 5+ samples)
- [ ] Handle under Nitrogen ($37 per sample, subject to rush surcharge)
- [ ] Handle under Argon ($40 per sample, subject to rush surcharge)

Replicates

( ) Single ( ) Duplicate ( ) Triplicate ( ) Other ____

(●) Duplicate if 1st analysis disagrees with theory/range

Note: A 100% surcharge is added for each additional replicate

Data Delivery

- [ ] Raw Data ($60 per sample)

Sample Information

Sample Identification	Amount	Sample Composition
JPD201	14 mg	C18H30S4Cl2Pd

Sample Disposition After Testing

The sample remains your property at all times. Select one of these options

- (●) **Discard** [By checking this box, you certify that the material is suitable for disposal in a sanitary landfill. This is not available for hazardous samples.]
- ( ) **Return to Sender** [Non-hazardous: $8/spl or $19/batch of 3 or more. Hazardous: $90/spl or batch.]

Hazards

- ( ) SDS Required if hazardous
- (●) No SDS
- [ ] Unknown
- [ ] Biohazard ($6 per sample)
- [ ] Cytotoxic
- [ ] Inhalation
- [ ] Pyrophoric
- [ ] Reproductive

Testing Information

Test Requested	Detection Limit	Theory/Range*	Method/Procedure
C, H, and S		%C, 39.16	
		%H, 5.48	
		%S, 23.24	

*Are the stated Theory/Range values regulatory specifications? ( ) Yes ( ) No

Notes & Comments

Internal	Date Received	Internal Lab ID

Ship samples to 2323 Sycamore Dr Knoxville, TN 37921. If rush turnaround time is requested, please indicate on the outside of the shipping box by writing RUSH or PRIORITY on your shipping label. Pricing subject to change. For the most updated pricing, please contact us at labinfo@galbraith.com. A sample submitted for testing constitutes acceptance of Galbraith's [illegible]

Figure S26. Elemental analysis request form for [Cl_2Pd(tptbz)], Galbraith Labs.

Report Number: 141313 GALBRAITH LABORATORIES, INC. Report Date: 2022-08-01

Laboratory Report

Report prepared for:
James P Donahue
Tulane University
Dept of Chem
Stern Hall, Room 2015
6400 Freret St
New Orleans, LA 70118
Phone: 504-862-3562
Email: donahue@tulane.edu

Report prepared by:
Debbie S Robertson

Purchase Order:
Visa,Stephenson,7/20/22

For further assistance, contact:
Debbie S Robertson
Report Production Coordinator
PO Box 51610
Knoxville, TN 37950 -1610
(865) 546-1335
debbierobertson@galbraith.com

Sample: JPD201
Lab ID: 2022-Q-5099 **Received:** 2022-07-20

Analysis	Method	Result	Basis	Sample Amount Used	Date (Time)
C : Carbon					
	GLI Procedure ME-14	39.17 %	As Received	2.145 mg	2022-07-26
H : Hydrogen					
	GLI Procedure ME-14	5.14 %	As Received	2.145 mg	2022-07-26
S : Sulfur					
	GLI Procedure E16-3	20.84 %	As Received	1.893 mg	2022-07-28
	GLI Procedure E16-3	20.78 %	As Received	2.588 mg	2022-07-28

Signatures:

Published By:	Debbie.S.Robertson	2022-08-01T20:16:53.76-04:00
Created By:	Debbie.S.Robertson	2022-08-01T20:16:03.463-04:00

- Physical signatures are on file.
- "Published By" signature indicates authorized release of data.

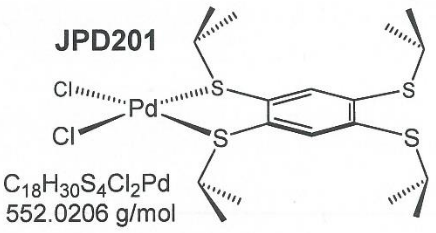

Copyright 2022 Galbraith Laboratories, Inc.
Reported results are only applicable to the item tested.
This report shall not be reproduced, except in full, without the written approval of the laboratory.

Page 1 of 1

Figure S27. Elemental analysis results for [Cl_2Pd(tptbz)] from Galbraith Labs.

Analysis Form

Address

MIKROLAB
Mikroanalytisches Laboratorium Kolbe

Mikroanalytisches Laboratorium Kolbe
c/o Fraunhofer-Insitut UMSICHT
Building G - Osterfelderstr, 3
D-46047 Oberhausen

Tel. +49 - (0)208 - 32502
Fax +49 - (0)208 - 382314

www.mikro-lab.de
info@mikro-lab.de

Order Order number: JPD206

Name: James P. Donahue

Address: Department of Chemistry, Tulane University
6400 Freret Street, Stern Hall Room 2015
New Orleans, Louisiana 70118-5698, USA

E-Mail-Address: donahue@tulane.edu

Sample name: JPD206

Elements to be determined: C, H

Other elements contained: S, Cl, Pt

Single determination [X] Double determination in case of deviation [ ] [ ] %

Double determination [ ] Absolute deviation for a double determination (Std. 1%)

Sample data

The sample is under: Argon [ ] Nitrogen [ ] Air [X]
Vacuum [ ] Other ______

	Yes	No		Yes	No
Moisture sensitive	[ ]	[X]	Explosive	[ ]	[X]
Hygroscopic	[ ]	[X]	Sublimated	[ ]	[X]
Inhomogeneous	[ ]	[X]	Volatile	[ ]	[X]

Molecular formula $C_{18}H_{30}S_4Cl_2Pt$

Expected values in % wt

C: 33.74%
H: 4.72%
N:
S: 20.02%
Cl: 11.07%
Pt: 30.45%

Molecular structure

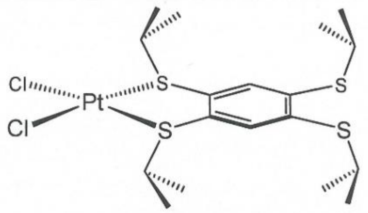

640.6862 g/mol

Special requests

	Yes	No
Handling under inert gas (Argon)	[ ]	[X]
Express treatment (max. 3 working days)	[ ]	[X]
CHN surcharge	[ ]	[ ]

Sample return: Yes [ ] No [X]

(A combustion surcharge is strongly recommended when metals, silicon, fluorine or nitrogen containing ring compounds are present to avoid minor measurements)

Drying before analysis desired Yes [ ] No [X]

Conditions for drying ______ mbar ______ °C ______ Std.

Other wishes ______

Date 9/13/2022 Signature ______

Version 01/2020

Figure S28. Elemental analysis request form for [Cl_2Pt(tptbz)], Kolbe Microanalytical Laboratory.

MIKROLAB
Mikroanalytisches Laboratorium Kolbe

Professor James P. Donahue
Department of Chemistry
Tulane University
6400 Freret St.
New Orleans, Louisiana 70118-5698, USA

Address : Osterfelder Str. 3
D-46047 Oberhausen
Phone : +49 - (0)208 - 32502
Fax : +49 - (0)208 - 382314
Email : info@mikro-lab.de
Website : www.mikro-lab.de

Date : 06.10.2022

Sample Name	% C	% H	% N										V2O5
JPD205	29,65	4,97	3,84										x
JPD206	33,71	4,77											x

Kind regards
Patrick Springer

JPD206

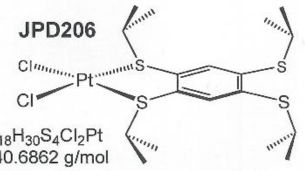

$C_{18}H_{30}S_4Cl_2Pt$
640.6862 g/mol

Figure S29. Elemental analysis results for [Cl_2Pt(tptbz)], Kolbe Microanalytical Laboratory.

Address

Mikroanalytisches Laboratorium Kolbe
c/o Fraunhofer-Insitut UMSICHT
Building G - Osterfelderstr, 3
D-46047 Oberhausen

Tel. +49 - (0)208 - 32502
Fax +49 - (0)208 - 382314

www.mikro-lab.de
info@mikro-lab.de

MIKROLAB
Mikroanalytisches Laboratorium Kolbe

Analysis Form

Order Order number: JPD213

Name: James P. Donahue

Address: Department of Chemistry, Tulane University
6400 Freret Street, Stern Hall Room 2015
New Orleans, Louisiana 70118-5698, USA

E-Mail-Address: donahue@tulane.edu

Sample name: JPD213

Elements to be determined: C, H, N

Other elements contained: S, Pd

Single determination [X] Double determination in case of deviation [ ] %

Double determination [ ] Absolute deviation for a double determination (Std. 1%)

Sample data

The sample is under: Argon [ ] Nitrogen [ ] Air [X]
Vacuum [ ] Other ____

	Yes	No		Yes	No
Moisture sensitive	[ ]	[X]	Explosive	[ ]	[X]
Hygroscopic	[ ]	[X]	Sublimated	[ ]	[X]
Inhomogeneous	[ ]	[X]	Volatile	[ ]	[X]

Molecular formula $C_{22}H_{30}N_2S_6Pd$

Expected values in % wt

C: 42.53%
H: 4.87%
N: 4.51%
S: 30.96%
Pd: 17.13%

Molecular structure

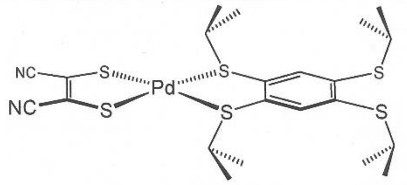

$C_{22}H_{30}N_2S_6Pd$
621.3096 g/mol

Special requests

	Yes	No		Yes	No
Handling under inert gas (Argon)	[ ]	[X]	Sample return	[ ]	[X]
Express treatment (max. 3 working days)	[ ]	[X]			
CHN surcharge	[ ]	[ ]			

(A combustion surcharge is strongly recommended when metals, silicon, fluorine or nitrogen containing ring compounds are present to avoid minor measurements)

Drying before analysis desired Yes [ ] No [X]

Conditions for drying ____ mbar ____ °C ____ Std.

Other wishes ____

Date 4/7/2023 Signature ____

Version 01/2020

Figure S30. Elemental analysis request form for [(mnt)Pd(tptbz)], Kolbe Microanalytical Laboratory.

Analysis Form

Address

MIKROLAB
Mikroanalytisches Laboratorium Kolbe

Mikroanalytisches Laboratorium Kolbe
c/o Fraunhofer-Insitut UMSICHT
Building G - Osterfelderstr, 3
D-46047 Oberhausen

Tel. +49 - (0)208 - 32502
Fax +49 - (0)208 - 382314

www.mikro-lab.de
info@mikro-lab.de

Order Order number: JPD214

Name: James P. Donahue

Address: Department of Chemistry, Tulane University
6400 Freret Street, Stern Hall Room 2015
New Orleans, Louisiana 70118-5698, USA

E-Mail-Address: donahue@tulane.edu

Sample name: JPD214

Elements to be determined: C, H, N

Other elements contained: S, Pt

Single determination [X] Double determination in case of deviation [ ] [ ] %

Double determination [ ] Absolute deviation for a double determination (Std. 1%)

Sample data

The sample is under: Argon [ ] Nitrogen [ ] Air [X] Vacuum [ ] Other ______

	Yes	No		Yes	No
Moisture sensitive	[ ]	[X]	Explosive	[ ]	[X]
Hygroscopic	[ ]	[X]	Sublimated	[ ]	[X]
Inhomogeneous	[ ]	[X]	Volatile	[ ]	[X]

Molecular formula $C_{22}H_{30}N_2S_6Pt$

Expected values in % wt

C: 37.22%
H: 4.26%
N: 3.95%
S: 27.10%
Pt: 27.48%

Molecular structure

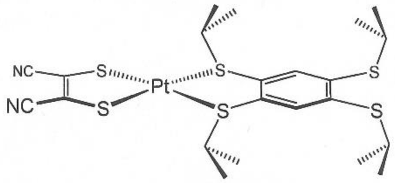

$C_{22}H_{30}N_2S_6Pt$
709.9696 g/mol

Special requests

	Yes	No		Yes	No
Handling under inert gas (Argon)	[ ]	[X]	Sample return	[ ]	[X]
Express treatment (max. 3 working days)	[ ]	[X]			
CHN surcharge	[ ]	[ ]			

(A combustion surcharge is strongly recommended when metals, silicon, fluorine or nitrogen containing ring compounds are present to avoid minor measurements)

Drying before analysis desired Yes [ ] No [X]

Conditions for drying ______ mbar ______ °C ______ Std.

Other wishes ______

Date 4/7/2023 Signature ______

Version 01/2020

Figure S31. Elemental analysis request form for [(mnt)Pt(tptbz)], Kolbe Microanalytical Laboratory.

MIKROLAB
Mikroanalytisches Laboratorium Kolbe

Professor James P. Donahue
Department of Chemistry
Tulane University
6400 Freret St.
New Orleans, Louisiana 70118-5698, USA

Address : Osterfelder Str. 3
D-46047 Oberhausen
Phone : +49 - (0)208 - 32502
Fax : +49 - (0)208 - 382314
Email : info@mikro-lab.de
Website : www.mikro-lab.de

Date : 17.04.2023

Sample Name	% C	% H	% N	% Cl	% S	% P						V2O5
JPD211	39,94	8,37	5,82									x
JPD212	60,14	2,90		25,34								x
JPD213	42,49	4,86	4,50									x
JPD214	37,21	4,27	3,94		27,06							x
JPD215	20,62	2,63			22,95	6,67						x

Kind regards
Patrick Springer

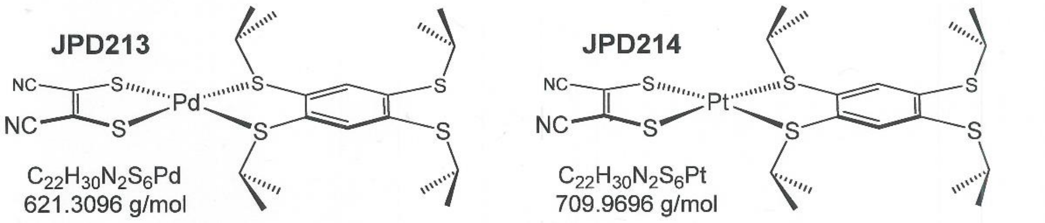

Figure S32. Elemental analysis for [(mnt)Pd(tptbz)] and [(mnt)Pd(tptbz)] from the Kolbe Microanalytical Laboratory of Oberhausen, Germany.

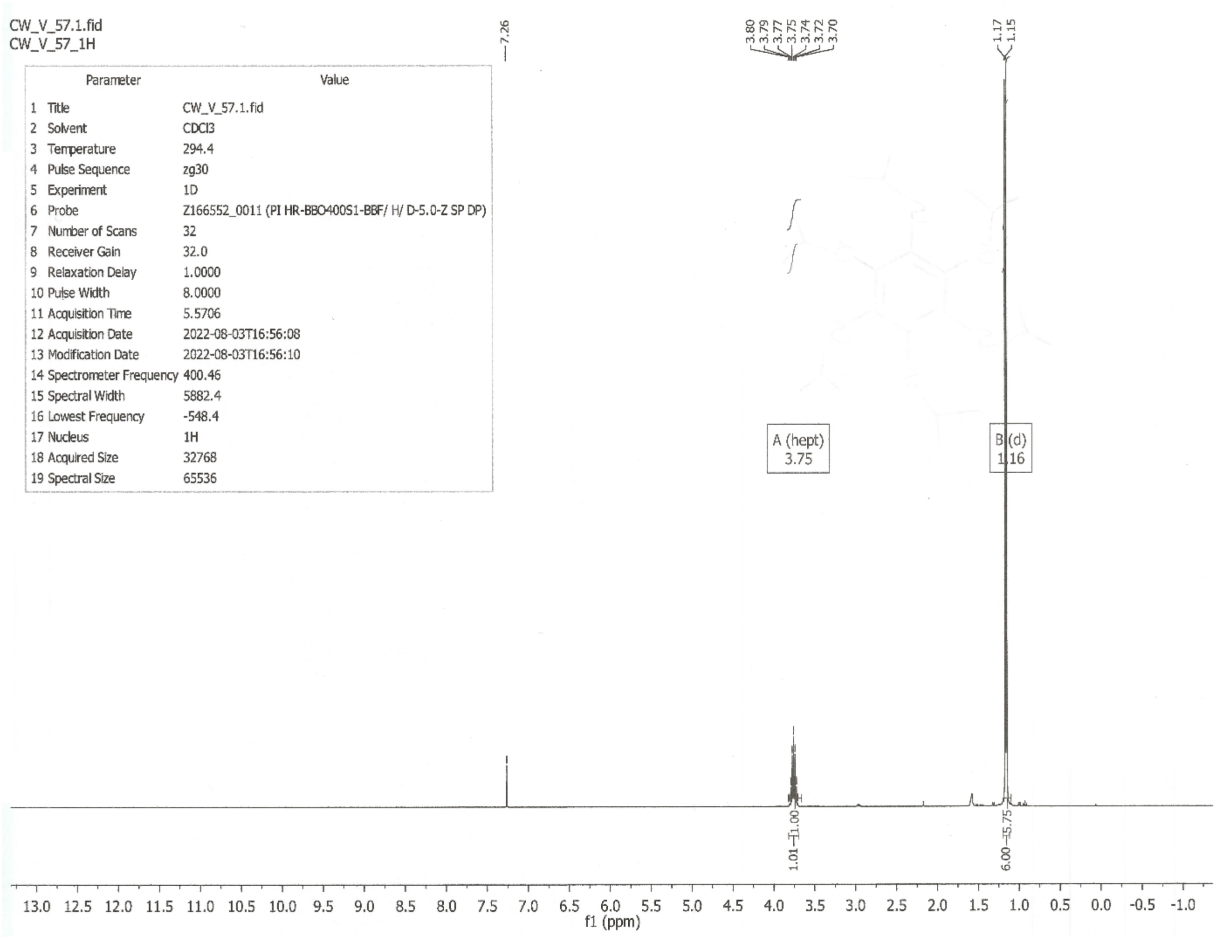

Figure S33. ^{1}H NMR spectrum ($CDCl_3$) of 1,2,3,4,5,6-hexakis$(^{i}PrS)_6C_6$.

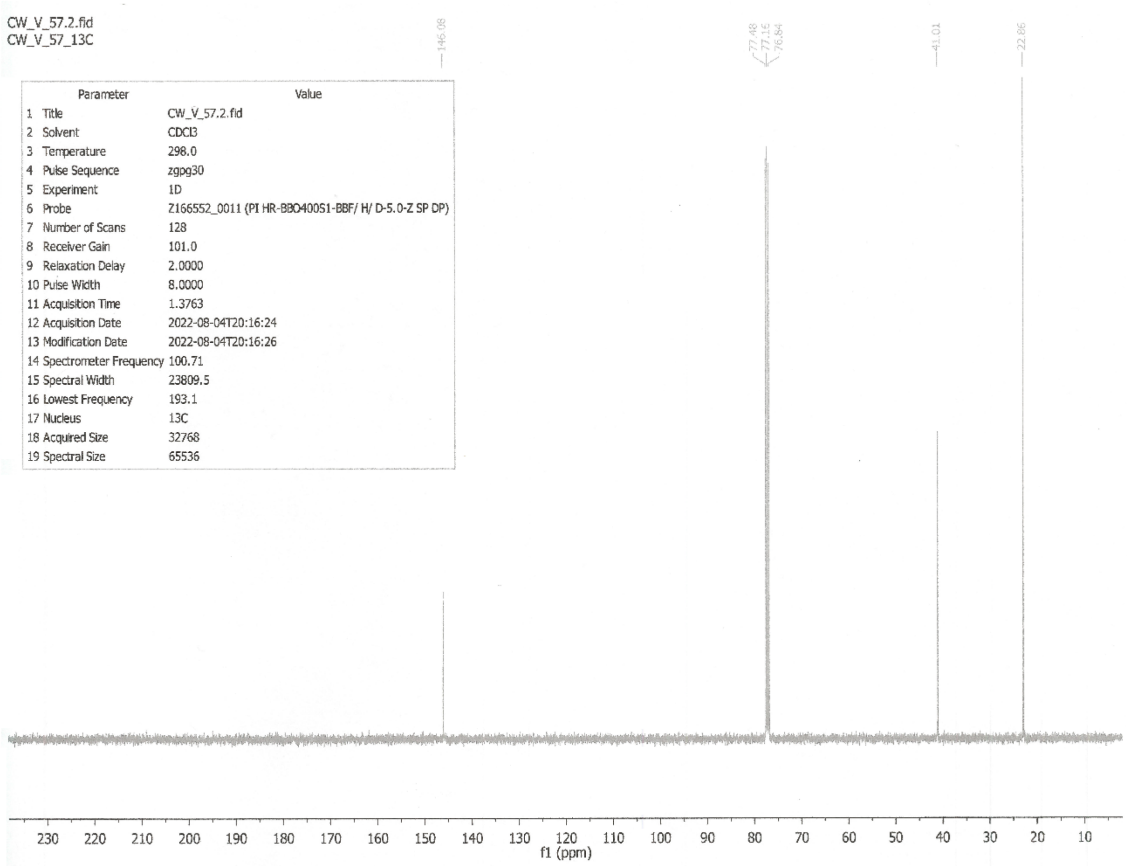

Figure S34. ^{13}C NMR spectrum ($CDCl_3$) of 1,2,3,4,5,6-hexakis(^{i}PrS)$_6$C$_6$.

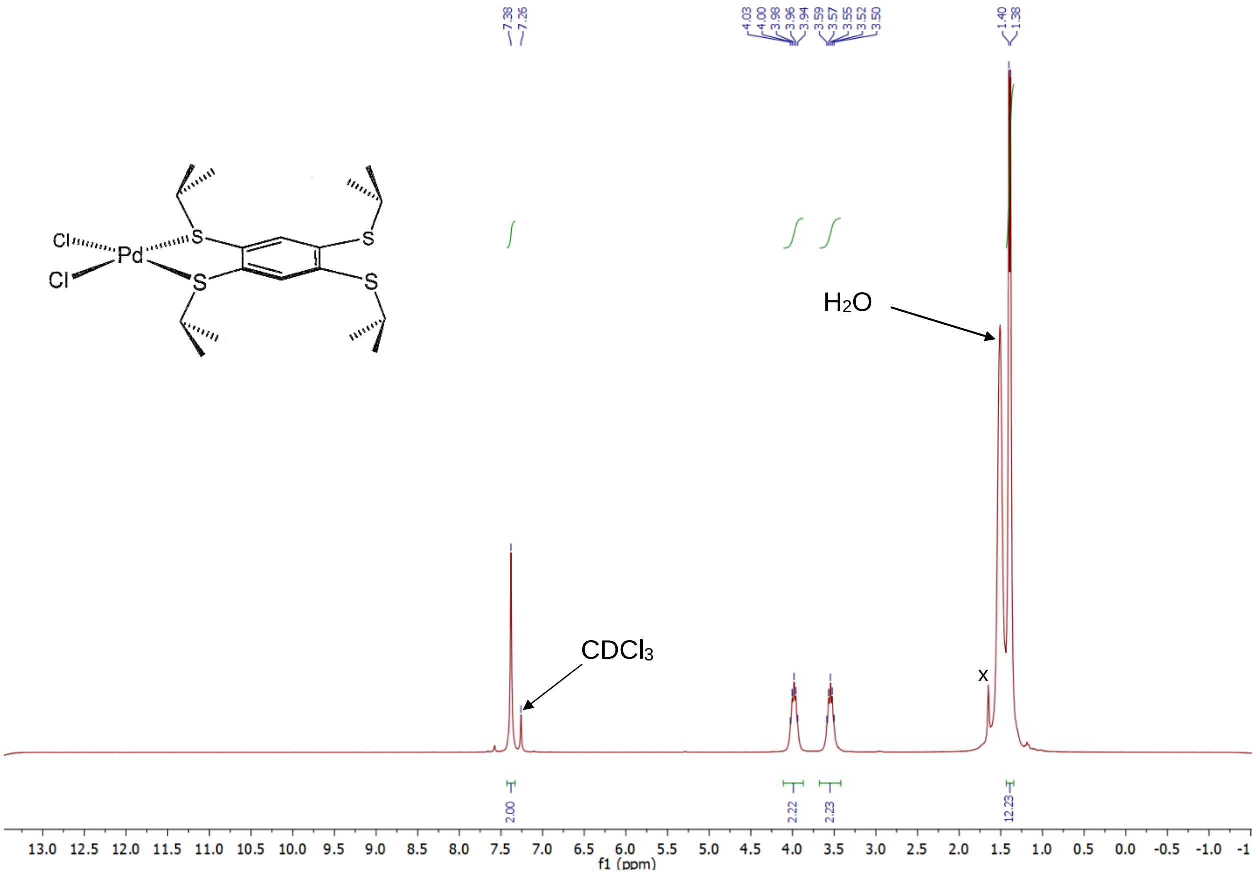

Figure S35. ^{1}H NMR spectrum of [Cl_2Pd(tptbz)] in $CDCl_3$ using 300 MHz NMR instrument.

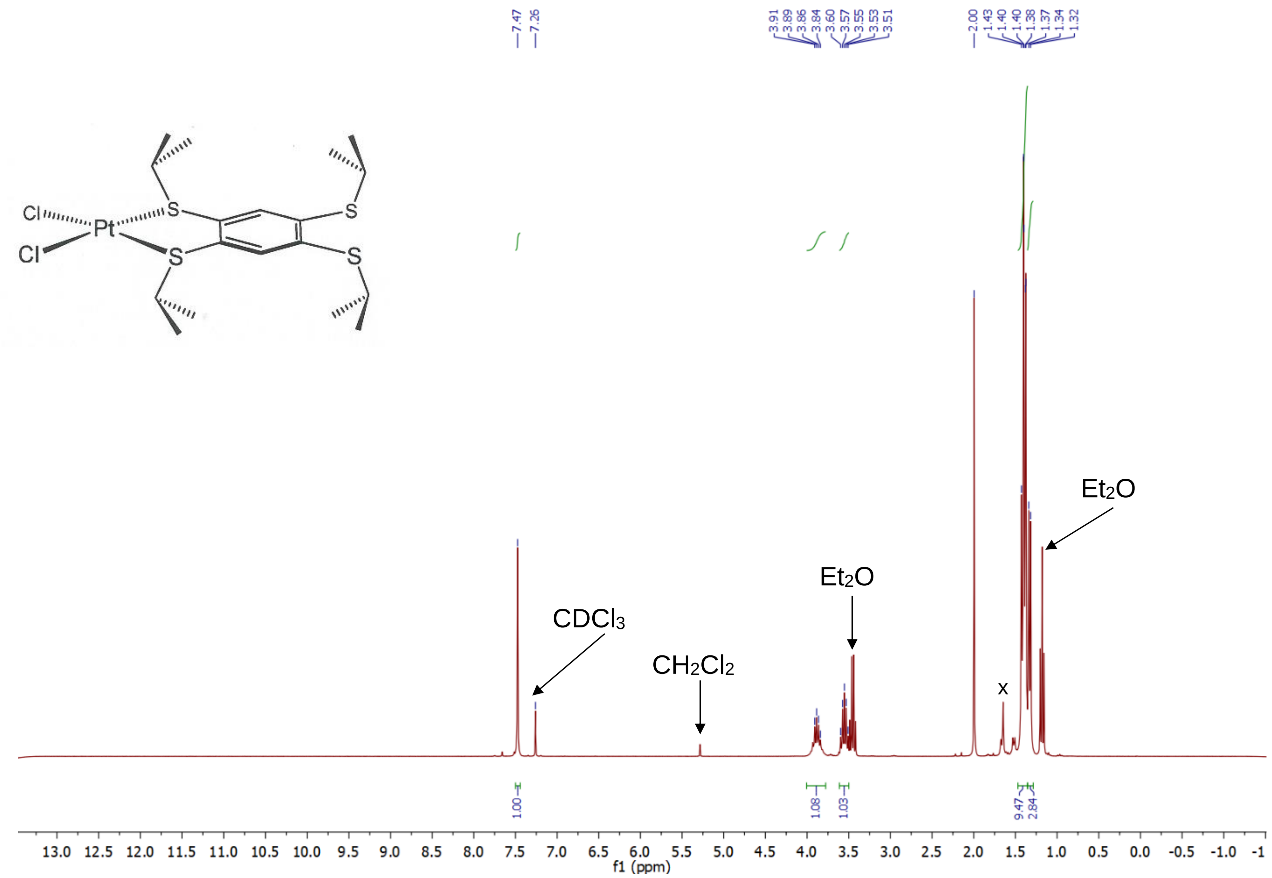

Figure S36. ^{1}H NMR spectrum of $[Cl_2Pt(tptbz)]$ in $CDCl_3$ using 300 MHz NMR instrument.

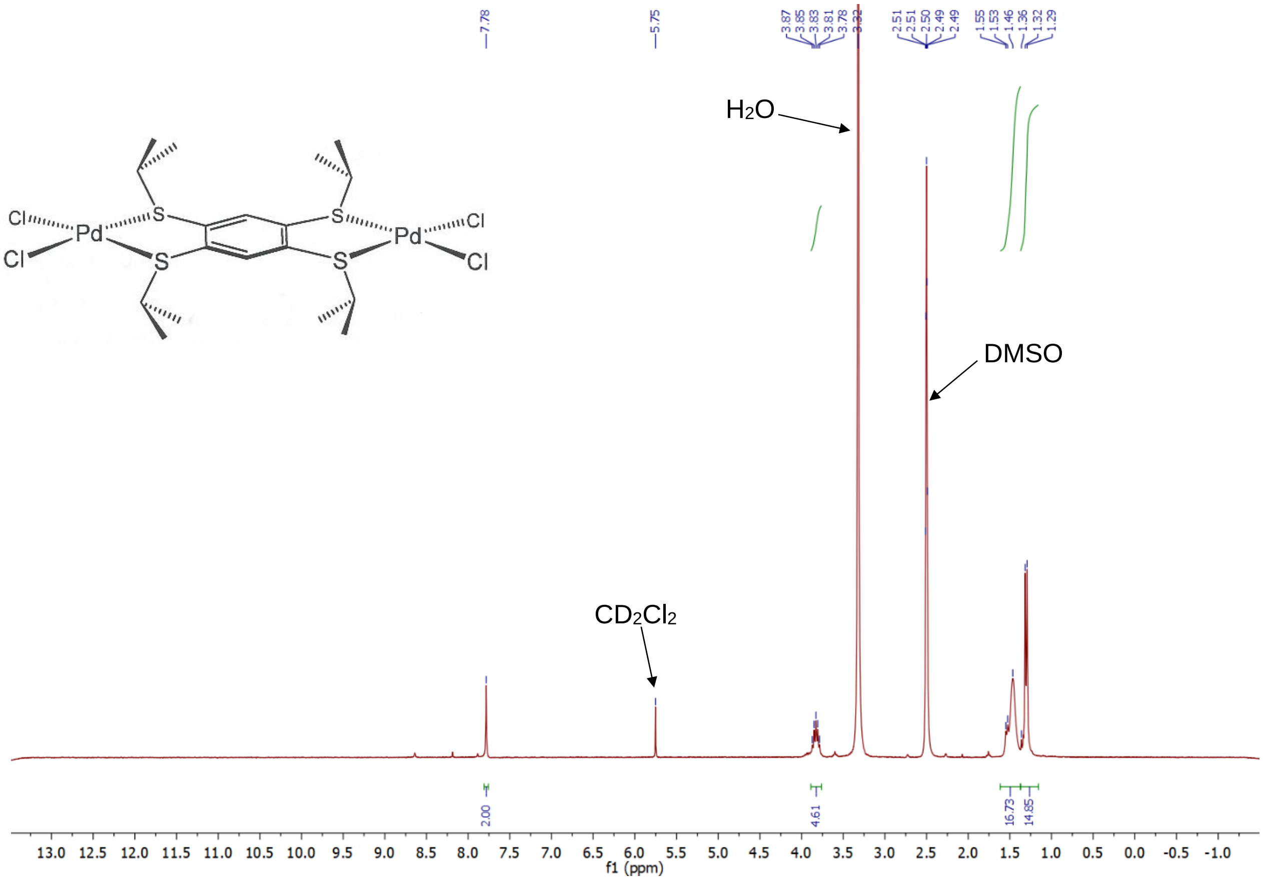

Figure S37. ^{1}H NMR spectrum of $[Cl_2Pd(tptbz)PdCl_2]$ in DMSO-d_6 using 300 MHz NMR instrument.

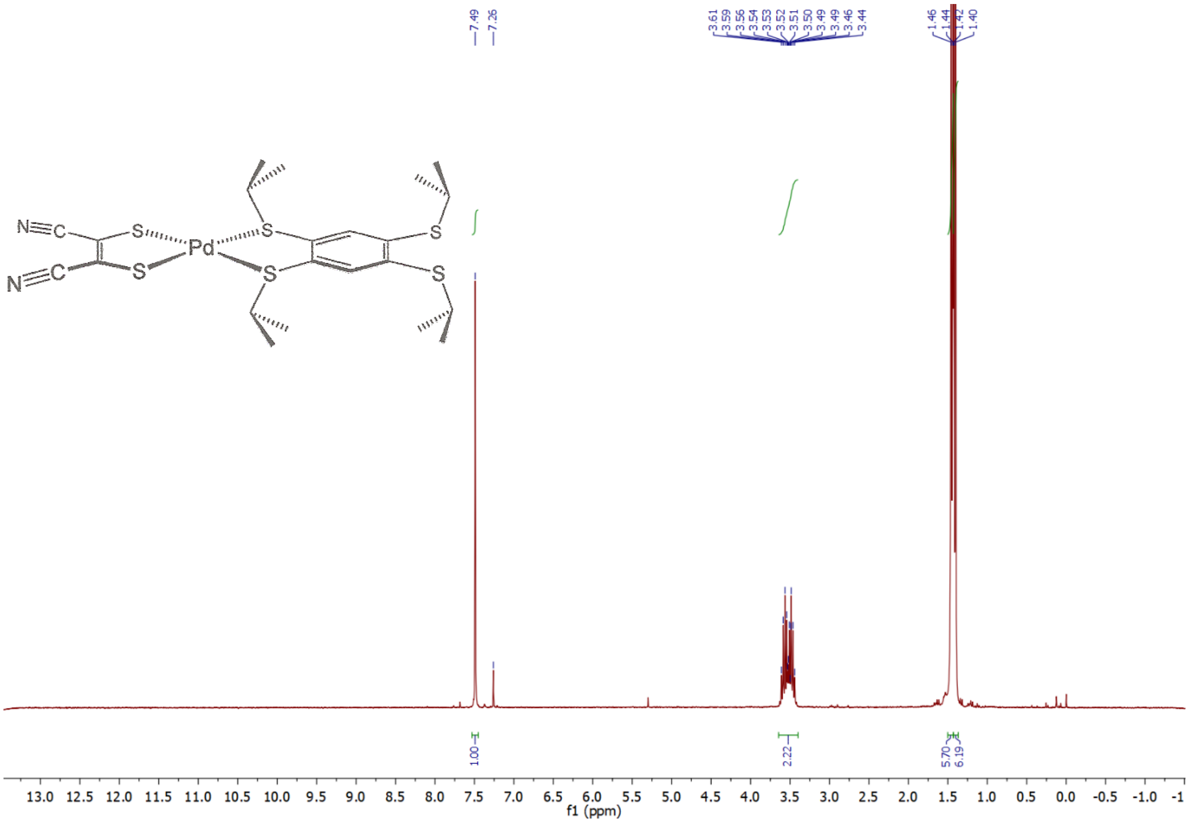

Figure S38. ^{1}H NMR spectrum of [(mnt)Pd(tptbz)] in $CDCl_3$ using 300 MHz NMR instrument.

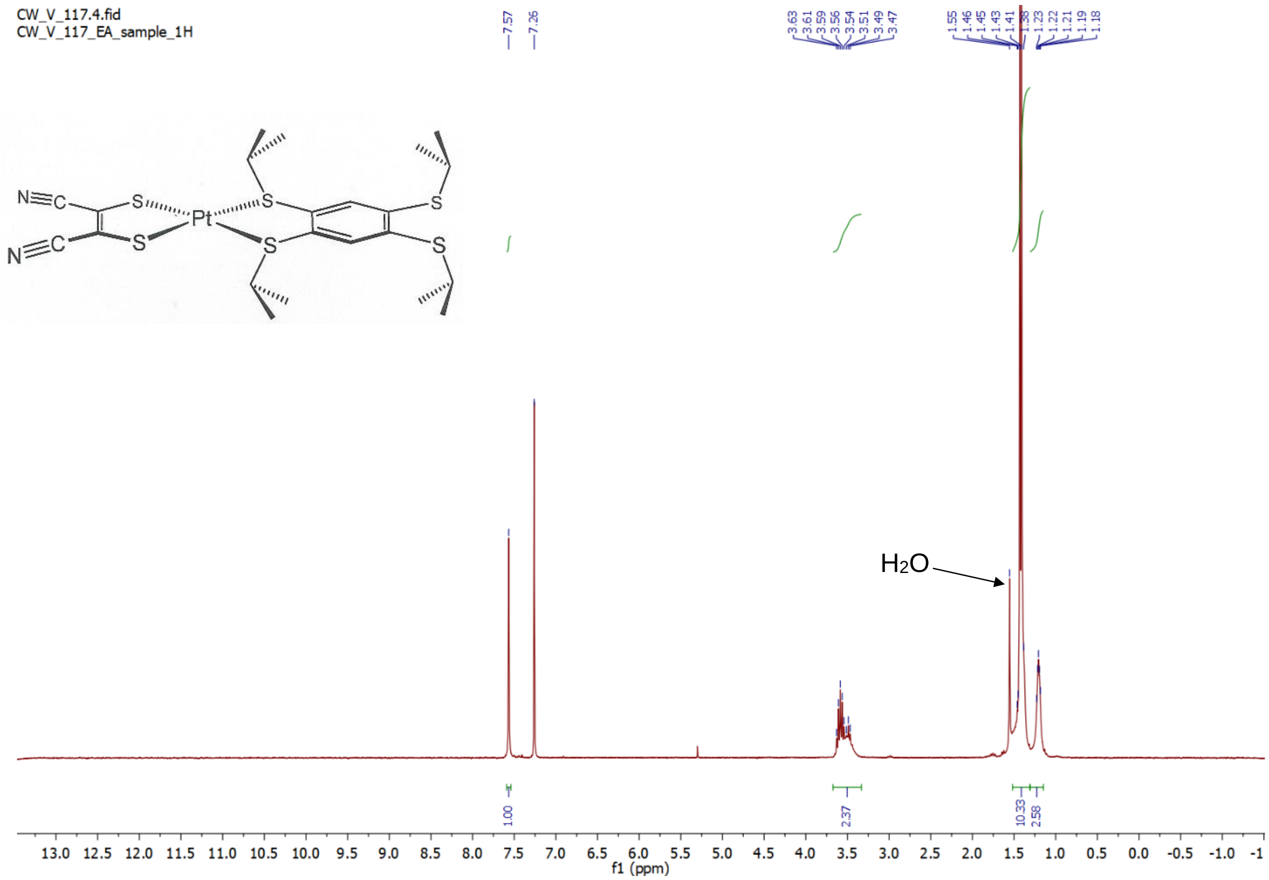

Figure S39. ^{1}H NMR spectrum of [(mnt)Pt(tptbz)] in $CDCl_3$ using 300 MHz NMR instrument.

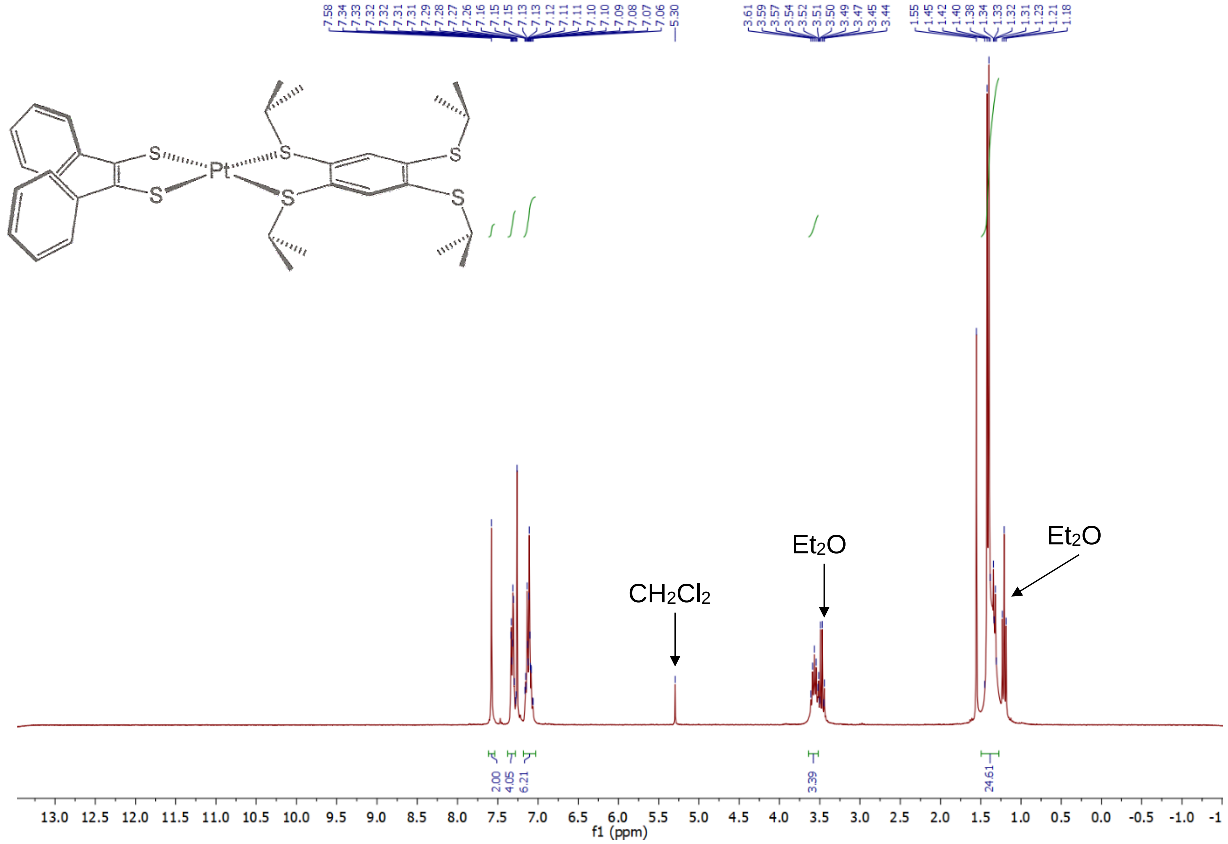

Figure S40. ^{1}H NMR spectrum of [(pdt)Pt(tptbz)] in $CDCl_3$ using 300 MHz NMR instrument.

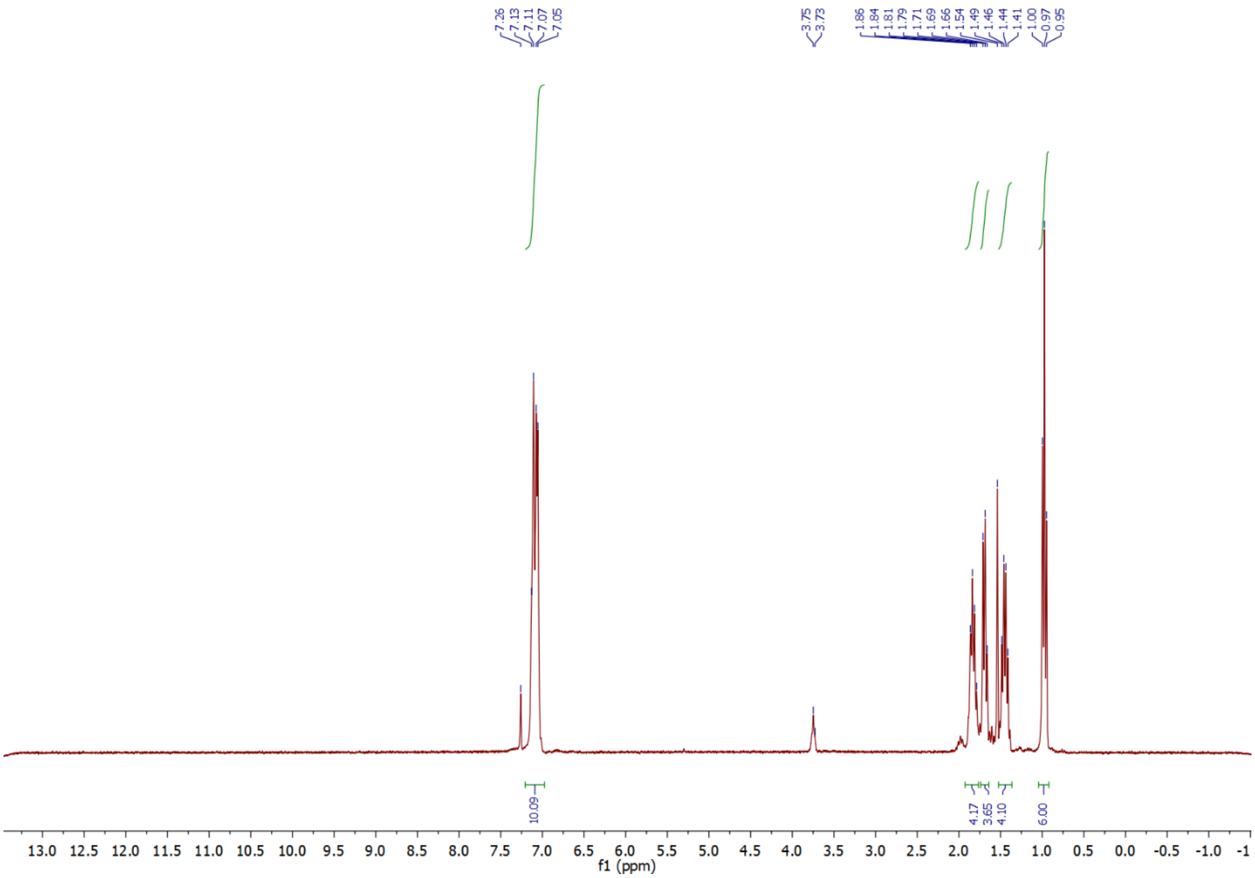

Figure S41. ^{1}H NMR spectrum of [(pdt)Sn^{n}Bu$_2$] in $CDCl_3$ using a 300 MHz NMR instrument.

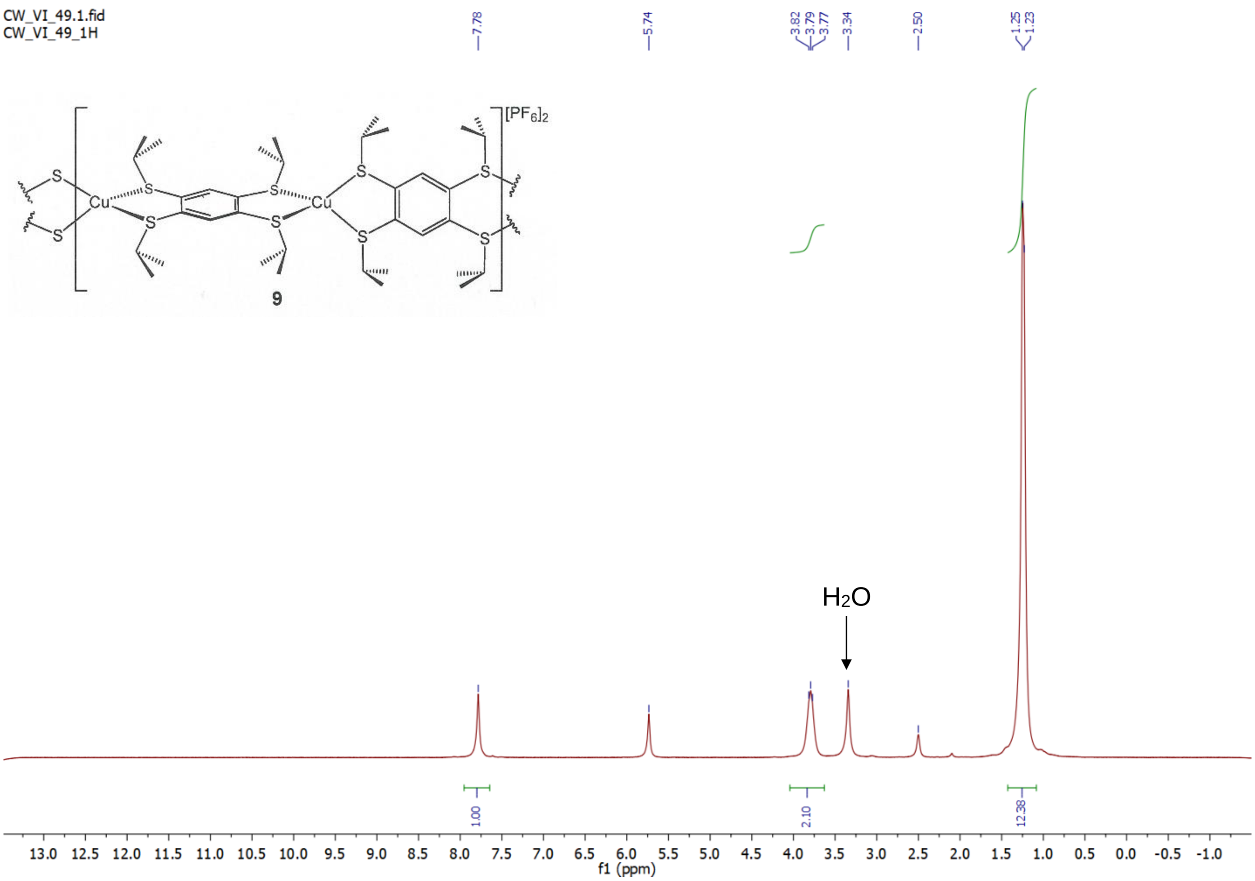

Figure S42. ^{1}H NMR spectrum of $[[Cu(tptbz)][PF_6]]_n$ in DMSO-d_6 using a 300 MHz NMR instrument.

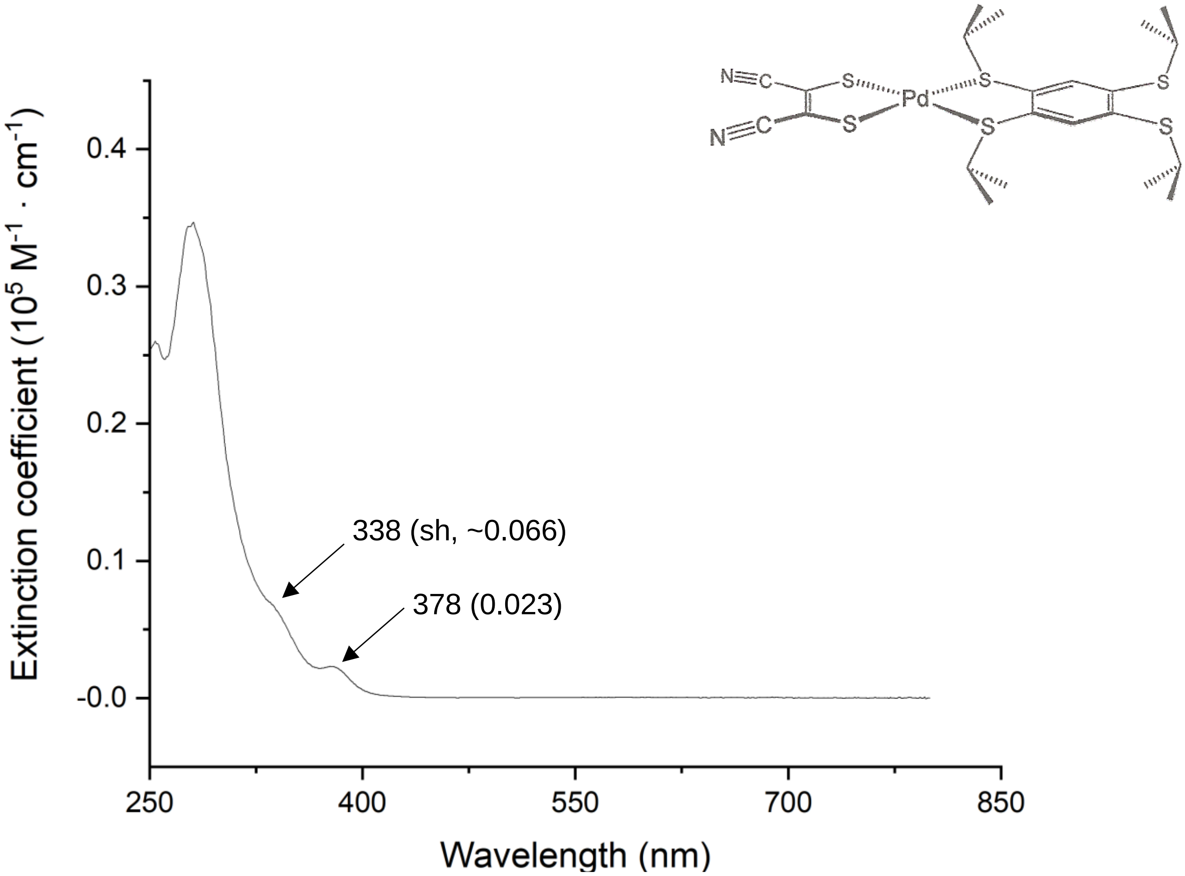

Figure S43. UV-vis spectrum of [(mnt)Pd(tptbz)] in CH_2Cl_2.

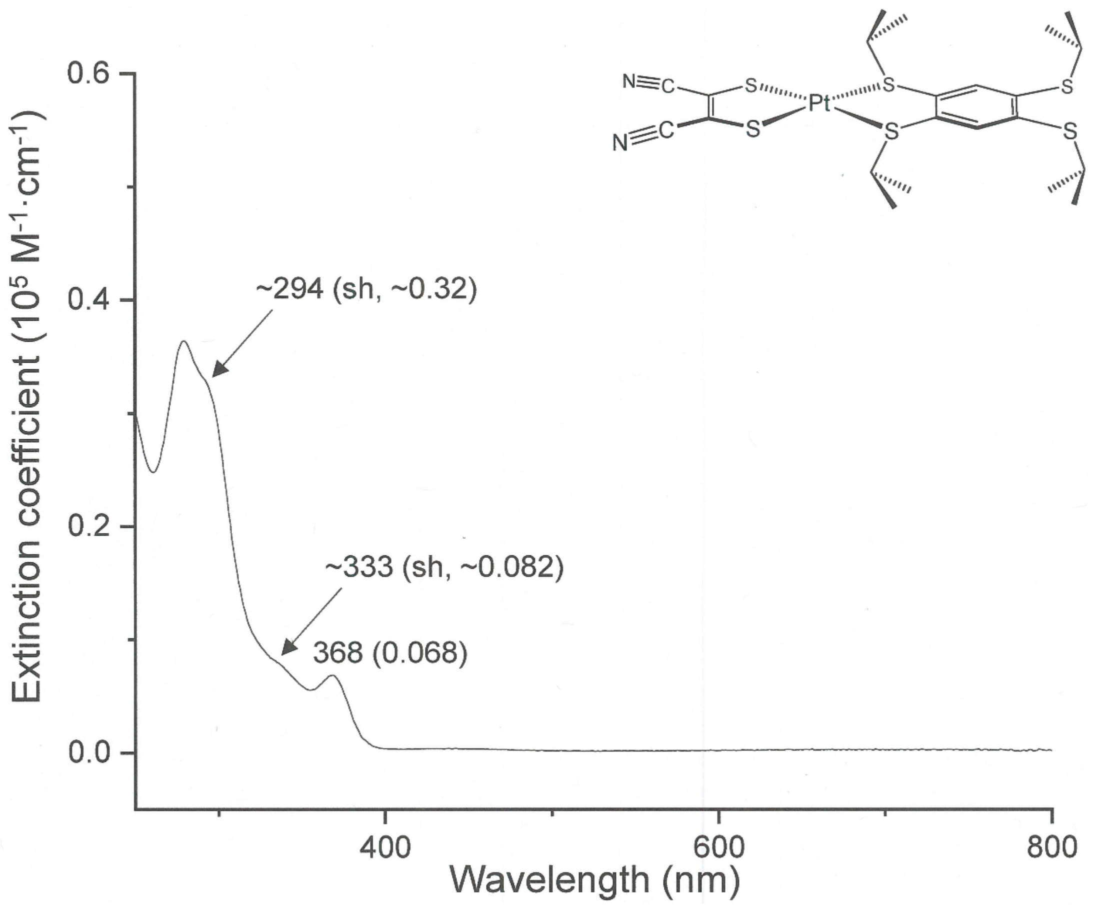

Figure S44. UV-vis spectrum of [(mnt)Pt(tptbz)] in CH_2Cl_2.

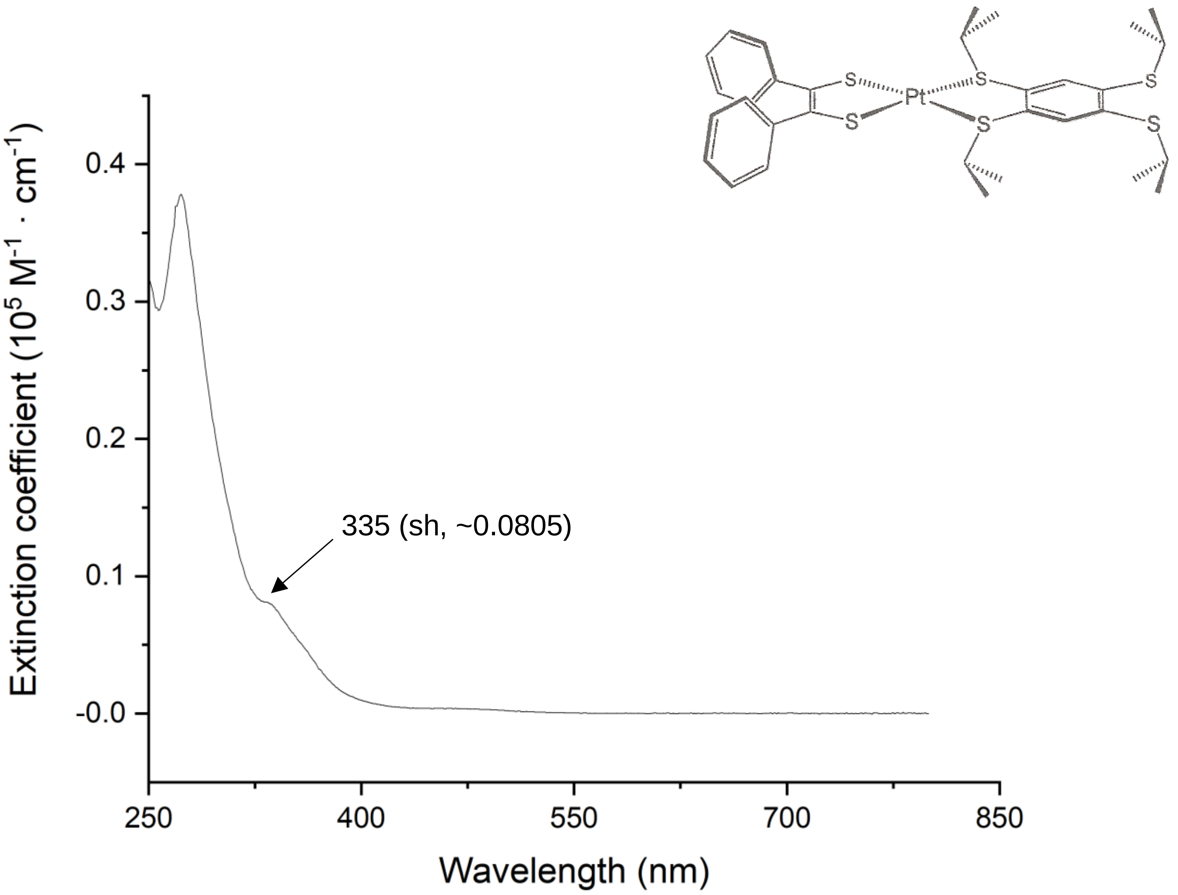

Figure S45. UV-vis spectrum of [(pdt)Pt(tptbz)] in CH_2Cl_2.

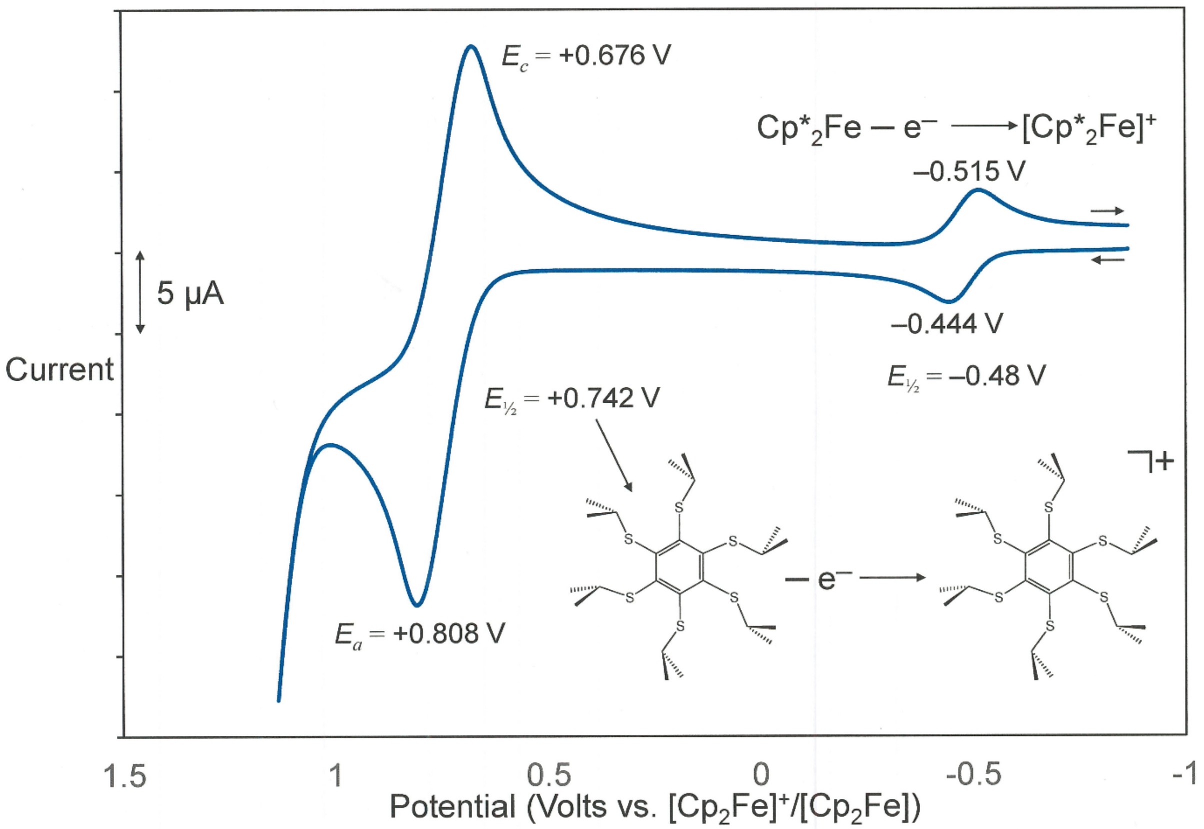

Figure S46. Cyclic voltammogram of $C_6(S^iP)_6$ in CH_2Cl_2 with $[^nBu_4N][PF_6]$ as supporting electrolyte.

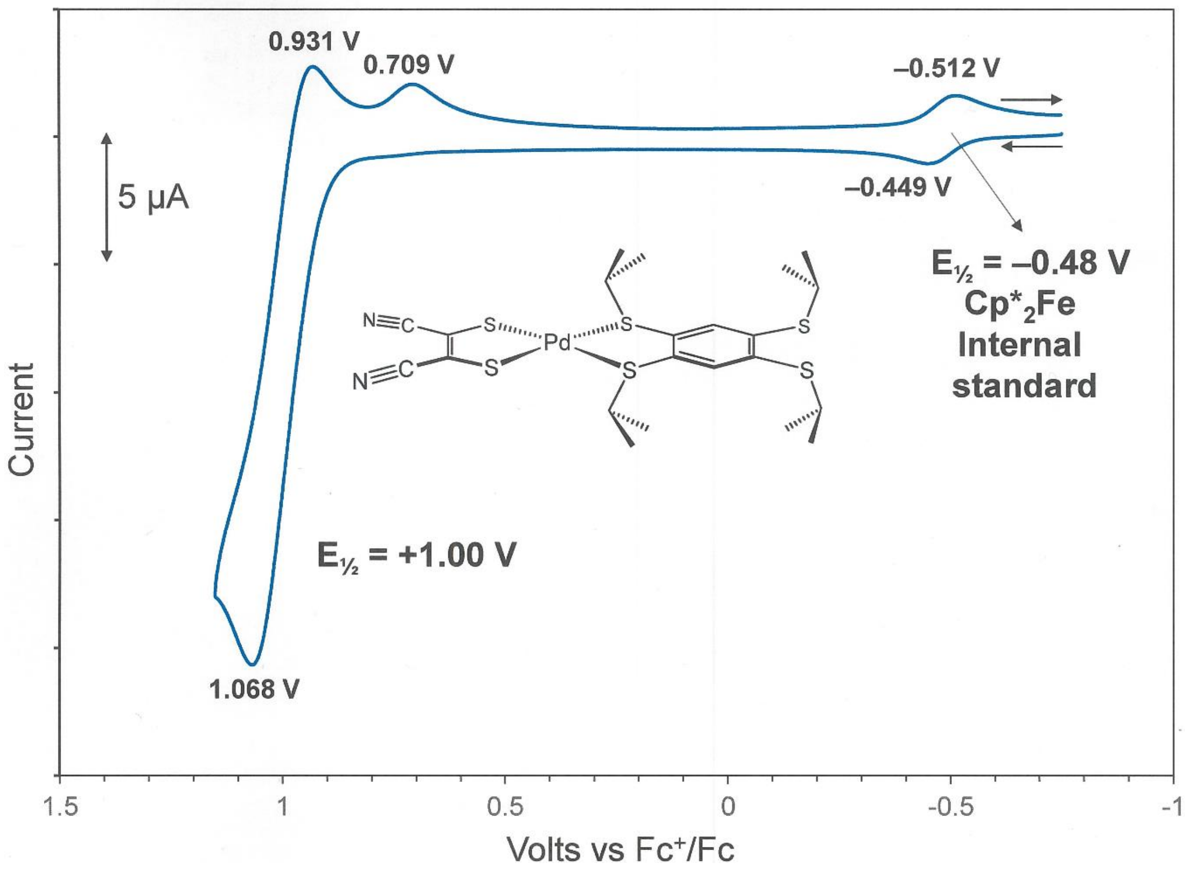

Figure S47. Cyclic voltammogram of [(mnt)Pd(tptbz)], **5**, in CH_2Cl_2 with [^{n}Bu_4N][PF_6] as supporting electrolyte and decamethylferrocene as internal standard, 100 mV/sec.

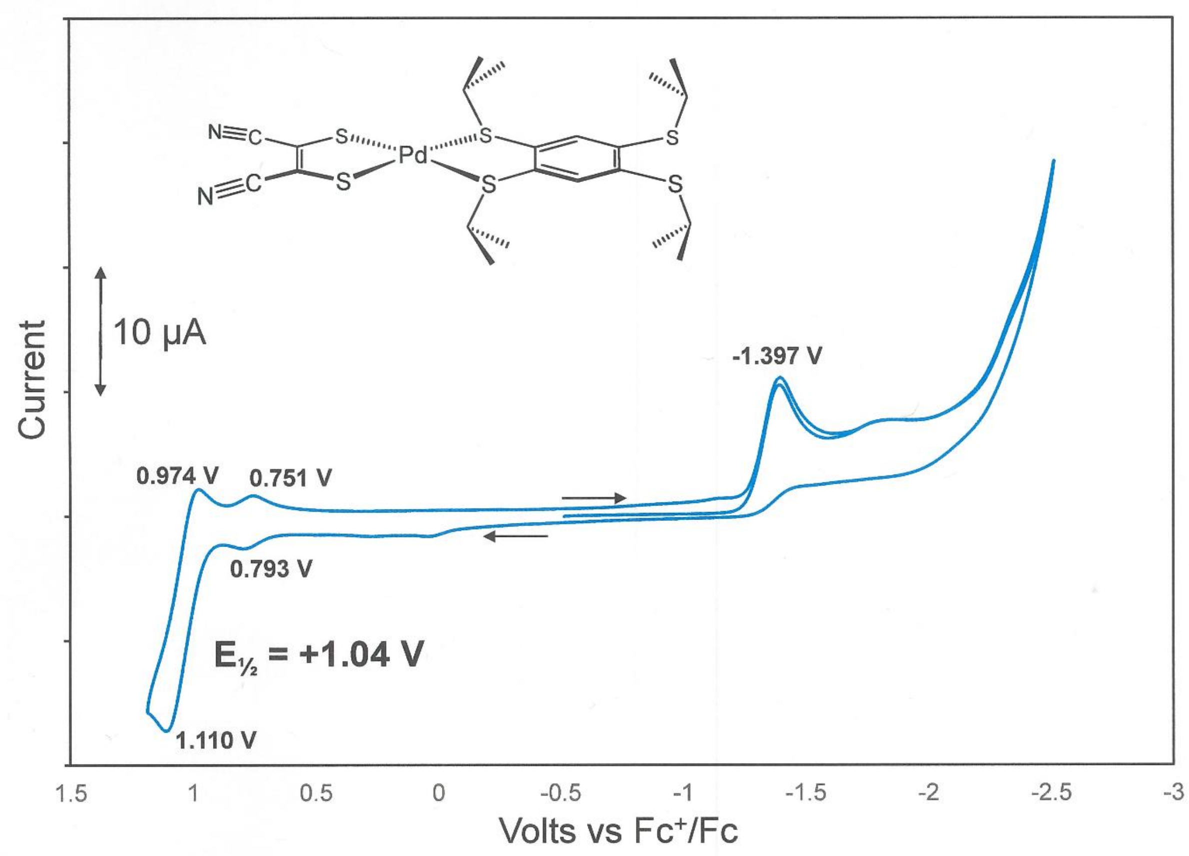

Figure S48. Full window cyclic voltammogram of [(mnt)Pd(tptbz)], **5**, in CH_2Cl_2 with [$^nBu_4N$][PF_6] as supporting electrolyte, 100 mV/sec.

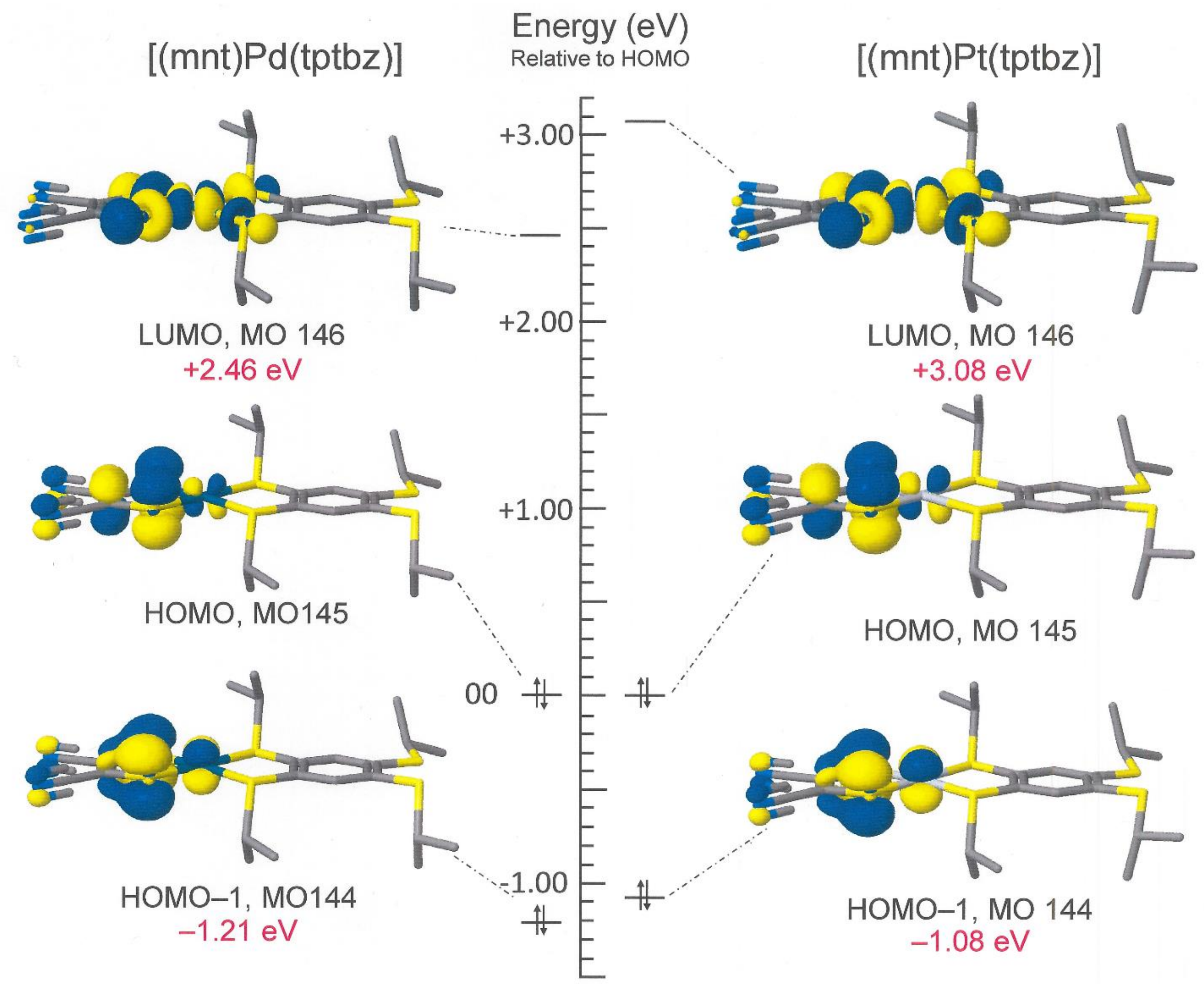

Figure S49. MO energy level diagrams calculated for [(mnt)Pd(tptbz)] and [(mnt)Pt(tptbz)] in the gas phase. Orbital images are presented at the 0.05 contour level.

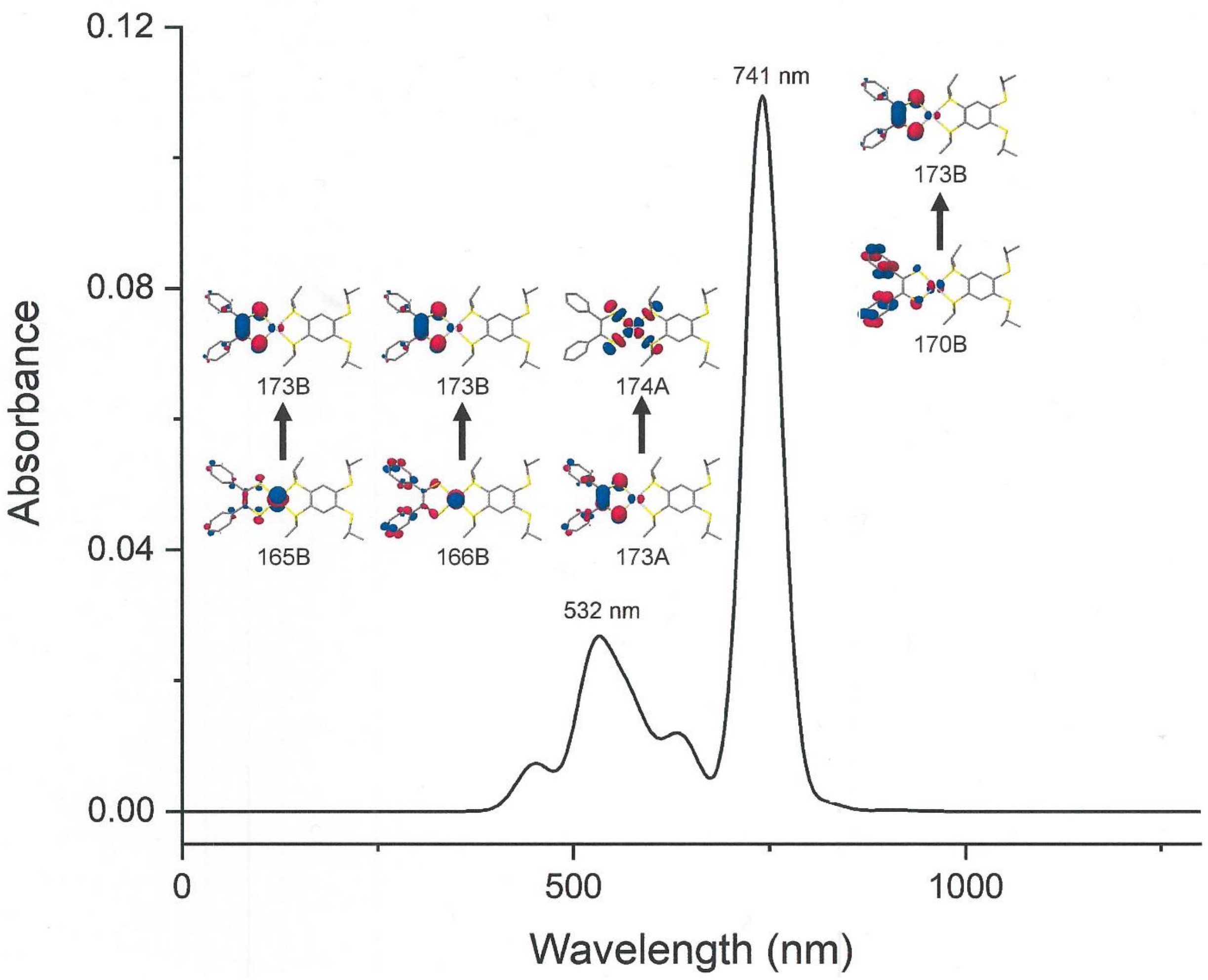

Figure S50. Time-dependent DFT calculated UV-vis spectrum of [**8**]$^+$.

Table S5. Final Atomic Coordinates for Optimized $[(Ph_2C_2S_2)Pt(tptbz)]^0$.

Energy: -2354346.0396237

Atom	Coordinates (Angstroms) X	Y	Z
Pt	0.91055	0.03008	-0.17587
S	2.56667	1.53143	0.44729
S	2.59175	-1.51496	-0.57454
S	-0.87112	1.61413	0.18102
S	-0.78837	-1.61599	-0.66743
S	-6.41260	1.35021	0.20649
S	-6.29082	-1.65343	-0.81848
C	5.29918	1.44286	0.54806
C	5.41133	2.18196	1.73918
H	4.61106	2.12423	2.47798
C	6.53330	2.97189	1.98781
H	6.60225	3.53215	2.92296
C	7.56509	3.04429	1.05122
H	8.44347	3.66326	1.24600
C	7.46333	2.32127	-0.13905
H	8.26131	2.37652	-0.88283
C	6.34208	1.53356	-0.39072
H	6.26291	0.98070	-1.32665
C	4.09100	0.62256	0.26815
C	4.10048	-0.67196	-0.13364
C	5.30447	-1.54042	-0.21071
C	5.58247	-2.29094	-1.36708
H	4.91879	-2.20616	-2.22845
C	6.69758	-3.12615	-1.42682
H	6.89764	-3.69452	-2.33789
C	7.55675	-3.23326	-0.33258
H	8.42984	-3.88754	-0.37961
C	7.28860	-2.49916	0.82460
H	7.94985	-2.58099	1.69011
C	6.17350	-1.66632	0.88761
H	5.96297	-1.10499	1.79780
C	-0.97921	2.74799	-1.33594
H	-1.98881	3.18001	-1.24363
C	0.06911	3.84255	-1.15803
H	-0.07998	4.40320	-0.22132
H	-0.01223	4.55195	-1.99941
H	1.08900	3.42454	-1.15652
C	-0.84727	1.98688	-2.64709
H	0.14622	1.51778	-2.72961
H	-0.97636	2.69343	-3.48636

Table S5, Continued. Final Atomic Coordinates for Optimized $[(Ph_2C_2S_2)Pt(tptbz)]^0$.

Energy: -2354346.0396237

Atom	Coordinates (Angstroms) X	Y	Z
H	-1.61407	1.20250	-2.74630
C	-0.69320	-2.78312	0.82435
H	0.37010	-3.06009	0.74456
C	-1.56601	-4.01693	0.61425
H	-1.38289	-4.48884	-0.36377
H	-2.63937	-3.77841	0.69735
H	-1.33255	-4.75782	1.39854
C	-0.93846	-2.07590	2.14976
H	-0.76408	-2.79027	2.97335
H	-1.97748	-1.71464	2.23204
H	-0.25107	-1.22568	2.27783
C	-2.36899	0.63025	-0.05236
C	-2.33291	-0.72228	-0.41959
C	-3.54603	-1.37923	-0.66082
H	-3.53312	-2.40419	-1.02808
C	-4.77906	-0.75181	-0.47048
C	-4.81613	0.60437	-0.05923
C	-3.59781	1.27090	0.13515
H	-3.58640	2.30914	0.45670
C	-6.05755	3.13356	0.59091
H	-5.23739	3.15701	1.32539
C	-5.68433	3.93357	-0.65797
H	-5.45814	4.97915	-0.38223
H	-6.51874	3.94058	-1.37820
H	-4.80277	3.51919	-1.17131
C	-7.32012	3.68023	1.26498
H	-7.54854	3.14183	2.19788
H	-8.19587	3.60516	0.59793
H	-7.17458	4.74640	1.50766
C	-6.28022	-2.96175	0.51643
H	-5.31863	-3.49176	0.42068
C	-7.41636	-3.93070	0.18739
H	-7.43464	-4.74389	0.93291
H	-7.29202	-4.38211	-0.80978
H	-8.39571	-3.42346	0.21896
C	-6.40162	-2.36360	1.91445
H	-6.35074	-3.16839	2.67019
H	-7.36001	-1.83305	2.03619
H	-5.58801	-1.65159	2.12402

Table S6. Final Atomic Coordinates for Optimized [$(Ph_2C_2S_2)Pt(tptbz)]^{1+}$.

Energy: -2354221.4536051

Atom	Coordinates (Angstroms) X	Y	Z
Pt	-0.87940	-0.04614	-0.09630
S	-2.60743	-1.56899	0.29579
S	-2.55668	1.54868	-0.40718
S	0.85925	-1.66779	0.26230
S	0.85044	1.59177	-0.43310
S	6.38339	-1.48351	0.49651
S	6.39832	1.43132	-0.47254
C	-5.30670	-1.45276	0.32209
C	-5.44461	-2.34966	1.39946
H	-4.65897	-2.41149	2.15301
C	-6.58933	-3.13247	1.52357
H	-6.68983	-3.81028	2.37243
C	-7.60410	-3.04849	0.56828
H	-8.49830	-3.66622	0.66452
C	-7.47218	-2.17161	-0.51156
H	-8.25737	-2.11055	-1.26652
C	-6.33907	-1.37393	-0.63390
H	-6.23875	-0.70188	-1.48519
C	-4.06955	-0.65767	0.16275
C	-4.04810	0.71898	-0.13286
C	-5.25163	1.57629	-0.18665
C	-5.43457	2.48492	-1.24755
H	-4.71382	2.51088	-2.06531
C	-6.54447	3.32501	-1.27340
H	-6.68199	4.01134	-2.11013
C	-7.47795	3.28716	-0.23571
H	-8.34471	3.94955	-0.25536
C	-7.29987	2.39890	0.82805
H	-8.02066	2.37321	1.64660
C	-6.20244	1.54440	0.85314
H	-6.06489	0.86357	1.69212
C	0.98985	-2.72706	-1.30943
H	1.98924	-3.17362	-1.18631
C	-0.07071	-3.82078	-1.23333
H	-0.00911	-4.39260	-0.29399
H	0.09075	-4.52265	-2.06865
H	-1.08786	-3.41034	-1.33284
C	0.93711	-1.89973	-2.58508
H	-0.04999	-1.42593	-2.71142
H	1.11517	-2.56523	-3.44762

Table S6, Continued. Final Atomic Coordinates for Optimized $[(Ph_2C_2S_2)Pt(tptbz)]^{1+}$.

Energy: -2354221.4536051

Atom	X	Y	Z
H	1.71099	-1.11704	-2.60148
C	0.69252	2.68377	1.11824
H	-0.37322	2.94975	1.03964
C	1.54169	3.94182	0.96532
H	1.34517	4.45852	0.01319
H	2.61819	3.72199	1.03820
H	1.29124	4.63587	1.78560
C	0.94481	1.91634	2.40815
H	0.76224	2.59141	3.26172
H	1.98717	1.56515	2.47543
H	0.26987	1.05190	2.50514
C	2.37107	-0.69532	0.13662
C	2.37377	0.66543	-0.18369
C	3.59756	1.32359	-0.36205
H	3.58711	2.36353	-0.67804
C	4.81908	0.66682	-0.18225
C	4.81362	-0.70816	0.19963
C	3.58437	-1.35935	0.34534
H	3.55755	-2.40504	0.64301
C	6.03157	-3.30624	0.61864
H	5.20258	-3.43228	1.33298
C	5.67907	-3.91979	-0.73596
H	5.45946	-4.99488	-0.61189
H	6.52093	-3.82068	-1.43984
H	4.79891	-3.44286	-1.19579
C	7.29013	-3.93150	1.22879
H	7.50743	-3.51971	2.22640
H	8.17114	-3.77082	0.58508
H	7.14191	-5.01939	1.32966
C	6.09592	3.26453	-0.37460
H	5.25719	3.49834	-1.04894
C	7.35900	3.92580	-0.93441
H	7.23905	5.02140	-0.90204
H	7.54538	3.63090	-1.97867
H	8.24741	3.66711	-0.33436
C	5.78471	3.71538	1.05134
H	5.56671	4.79789	1.06182
H	6.64460	3.53117	1.71534
H	4.91524	3.18719	1.47324

Table S7. Final Atomic Coordinates for Optimized [$(Ph_2C_2S_2)Pt(tptbz)]^{2+}$, Closed Shell Singlet.

Energy: -2354011.7767440

Atom	Coordinates (Angstroms) X	Y	Z
Pt	0.88026	-0.03393	0.06141
S	2.63292	-1.57684	-0.16065
S	2.57266	1.57767	0.24268
S	-0.85004	-1.65129	-0.29061
S	-0.84835	1.59650	0.35558
S	-6.36760	-1.47656	-0.54495
S	-6.39099	1.39010	0.51118
C	5.31368	-1.46712	-0.16226
C	5.42260	-2.55893	-1.06120
H	4.62737	-2.74385	-1.78345
C	6.55985	-3.35140	-1.06669
H	6.64913	-4.16602	-1.78572
C	7.59583	-3.10133	-0.15548
H	8.48436	-3.73418	-0.15674
C	7.49495	-2.04251	0.75340
H	8.29177	-1.86368	1.47578
C	6.37882	-1.21775	0.74519
H	6.29521	-0.41964	1.47981
C	4.08081	-0.69016	-0.09937
C	4.05472	0.76137	0.08956
C	5.25263	1.59213	0.08668
C	5.36036	2.69165	0.97672
H	4.59713	2.84433	1.73988
C	6.46021	3.53309	0.92109
H	6.55124	4.35370	1.63306
C	7.45725	3.32479	-0.04282
H	8.31596	3.99594	-0.08922
C	7.35573	2.25884	-0.94322
H	8.12086	2.11207	-1.70595
C	6.27908	1.38583	-0.87491
H	6.19236	0.58187	-1.60265
C	-0.98276	-2.71502	1.29227
H	-1.98417	-3.15020	1.14826
C	0.06759	-3.81742	1.22417
H	0.03891	-4.36497	0.26917
H	-0.14062	-4.54005	2.03083
H	1.08482	-3.42793	1.38699
C	-0.94601	-1.88312	2.56439
H	0.04259	-1.41812	2.71223
H	-1.14353	-2.54752	3.42320

Table S7, Continued. Final Atomic Coordinates for Optimized $[(Ph_2C_2S_2)Pt(tptbz)]^{2+}$, Closed Shell Singlet.

Energy: -2354011.7767440

Atom	Coordinates (Angstroms) X	Y	Z
H	-1.71653	-1.09750	2.56890
C	-0.70445	2.66176	-1.23148
H	0.35652	2.94869	-1.16352
C	-1.57519	3.90490	-1.08730
H	-1.37514	4.44674	-0.15030
H	-2.64747	3.66348	-1.14458
H	-1.34575	4.58390	-1.92599
C	-0.95998	1.85840	-2.49761
H	-0.80041	2.51847	-3.36714
H	-1.99768	1.49199	-2.54434
H	-0.27319	1.00235	-2.58922
C	-2.36784	-0.69115	-0.18045
C	-2.37409	0.66966	0.14728
C	-3.59309	1.32494	0.34894
H	-3.58346	2.36376	0.66788
C	-4.81690	0.66064	0.18295
C	-4.80796	-0.71269	-0.23485
C	-3.57479	-1.35980	-0.39432
H	-3.54895	-2.40292	-0.70108
C	-6.02823	-3.29219	-0.79785
H	-5.19531	-3.36568	-1.51513
C	-5.69020	-3.99981	0.51394
H	-5.48640	-5.06596	0.31383
H	-6.53379	-3.94058	1.21969
H	-4.80513	-3.57001	1.01054
C	-7.29038	-3.85581	-1.45906
H	-7.49776	-3.37090	-2.42513
H	-8.17313	-3.73751	-0.80912
H	-7.14866	-4.93399	-1.63883
C	-6.10313	3.22093	0.70744
H	-5.24217	3.34285	1.38346
C	-7.35262	3.76175	1.41006
H	-7.24228	4.84978	1.54798
H	-7.49700	3.30250	2.39983
H	-8.25983	3.59108	0.80714
C	-5.84753	3.89835	-0.63825
H	-5.64572	4.97168	-0.47859
H	-6.72817	3.81047	-1.29414
H	-4.98766	3.46211	-1.17129

Table S8. Final Atomic Coordinates for Optimized $[(Ph_2C_2S_2)Pt(tptbz)]^{2+}$, Singlet Diradical.

Energy: -2354012.3186549

Atom	Coordinates (Angstroms) X	Y	Z
Pt	0.88363	-0.03578	0.06601
S	2.63173	-1.57533	-0.18042
S	2.57160	1.57373	0.26574
S	-0.84938	-1.65566	-0.28665
S	-0.84741	1.59632	0.36793
S	-6.36225	-1.47208	-0.57201
S	-6.38503	1.39032	0.52873
C	5.31509	-1.46532	-0.18533
C	5.42360	-2.53784	-1.10564
H	4.62742	-2.70925	-1.83020
C	6.56114	-3.33075	-1.12744
H	6.64909	-4.13157	-1.86204
C	7.59787	-3.09870	-0.21327
H	8.48663	-3.73102	-0.22782
C	7.49707	-2.05797	0.71608
H	8.29498	-1.89196	1.44038
C	6.37961	-1.23459	0.72585
H	6.29680	-0.44916	1.47428
C	4.08305	-0.68426	-0.10937
C	4.05718	0.75518	0.09617
C	5.25377	1.59153	0.10143
C	5.36547	2.67196	1.01247
H	4.60495	2.81016	1.78110
C	6.46530	3.51521	0.96872
H	6.55841	4.32208	1.69601
C	7.45848	3.32612	-0.00221
H	8.31724	3.99779	-0.03874
C	7.35268	2.27807	-0.92270
H	8.11531	2.14505	-1.69050
C	6.27492	1.40508	-0.86801
H	6.18567	0.61366	-1.60925
C	-0.98394	-2.72501	1.29220
H	-1.98656	-3.15864	1.15199
C	0.06522	-3.82809	1.21439
H	0.02536	-4.37708	0.26067
H	-0.13427	-4.54881	2.02497
H	1.08417	-3.43816	1.36412
C	-0.93745	-1.89783	2.56712
H	0.05198	-1.43292	2.70839
H	-1.12770	-2.56559	3.42500

Table S8, Continued. Final Atomic Coordinates for Optimized $[(Ph_2C_2S_2)Pt(tptbz)]^{2+}$, Singlet Diradical.

Energy: -2354012.3186549

Atom	Coordinates (Angstroms) X	Y	Z
H	-1.70787	-1.11200	2.58121
C	-0.70504	2.67382	-1.21002
H	0.35478	2.96254	-1.13287
C	-1.57980	3.91369	-1.06362
H	-1.38939	4.44826	-0.12046
H	-2.65125	3.67103	-1.13332
H	-1.34434	4.60024	-1.89444
C	-0.94821	1.87876	-2.48387
H	-0.78211	2.54506	-3.34739
H	-1.98492	1.51085	-2.54289
H	-0.25860	1.02507	-2.57503
C	-2.36824	-0.69502	-0.17222
C	-2.37418	0.67024	0.15024
C	-3.59151	1.32519	0.34918
H	-3.58209	2.36346	0.66986
C	-4.81732	0.66085	0.18363
C	-4.80861	-0.71236	-0.23664
C	-3.57372	-1.36170	-0.38883
H	-3.54810	-2.40453	-0.69658
C	-6.03665	-3.29905	-0.76273
H	-5.19590	-3.40072	-1.46714
C	-5.71903	-3.96451	0.57580
H	-5.52578	-5.03862	0.41182
H	-6.56836	-3.87347	1.27117
H	-4.83284	-3.52886	1.06496
C	-7.29692	-3.87017	-1.42087
H	-7.49058	-3.41192	-2.40272
H	-8.18513	-3.72790	-0.78345
H	-7.16059	-4.95377	-1.56901
C	-6.11030	3.23060	0.65538
H	-5.24489	3.38187	1.31947
C	-7.35944	3.78519	1.34757
H	-7.25401	4.87764	1.44894
H	-7.49479	3.35884	2.35324
H	-8.26907	3.58999	0.75600
C	-5.86855	3.85706	-0.71723
H	-5.67779	4.93748	-0.59798
H	-6.75126	3.73597	-1.36496
H	-5.00535	3.41094	-1.23635

Table S9. Final Atomic Coordinates for Optimized $[(Ph_2C_2S_2)Pt(tptbz)]^{2+}$, Triplet.

Energy: -2354005.1080628

Atom	Coordinates (Angstroms) X	Y	Z
Pt	0.89693	-0.04371	0.07865
S	2.62394	-1.56479	-0.29900
S	2.56784	1.55322	0.38347
S	-0.85242	-1.67338	-0.24082
S	-0.84721	1.59485	0.37104
S	-6.34281	-1.45383	-0.64684
S	-6.36498	1.39344	0.54542
C	5.32416	-1.45460	-0.26509
C	5.46637	-2.41251	-1.29189
H	4.68716	-2.51567	-2.04736
C	6.61342	-3.19367	-1.36836
H	6.72379	-3.91466	-2.17906
C	7.62758	-3.05247	-0.41239
H	8.52264	-3.67301	-0.47280
C	7.49191	-2.11886	0.61903
H	8.27348	-2.01802	1.37281
C	6.35730	-1.32001	0.69376
H	6.24650	-0.61155	1.51322
C	4.09163	-0.65597	-0.15036
C	4.06713	0.72474	0.12306
C	5.26330	1.58307	0.15772
C	5.42603	2.54846	1.17413
H	4.69447	2.61494	1.97974
C	6.53664	3.38449	1.17618
H	6.66477	4.11131	1.97903
C	7.49160	3.29061	0.15601
H	8.35792	3.95343	0.15866
C	7.33390	2.34961	-0.86527
H	8.06847	2.28488	-1.66867
C	6.23655	1.49664	-0.86664
H	6.10682	0.78225	-1.67817
C	-0.98922	-2.77001	1.32198
H	-1.99098	-3.20960	1.19413
C	0.07082	-3.85958	1.20789
H	-0.00581	-4.41882	0.26257
H	-0.08148	-4.57221	2.03581
H	1.08880	-3.45073	1.30254
C	-0.91635	-1.95493	2.60351
H	0.06880	-1.47453	2.71606
H	-1.06722	-2.63525	3.45951

Table S9, Continued. Final Atomic Coordinates for Optimized $[(Ph_2C_2S_2)Pt(tptbz)]^{2+}$, Triplet.

Energy: -2354005.1080628

Atom	Coordinates (Angstroms) X	Y	Z
H	-1.69802	-1.18074	2.65164
C	-0.70532	2.71705	-1.17620
H	0.35130	3.00412	-1.05766
C	-1.59010	3.94849	-1.03110
H	-1.44334	4.45574	-0.06533
H	-2.65746	3.70752	-1.15911
H	-1.32088	4.66068	-1.82947
C	-0.89930	1.94595	-2.47287
H	-0.69924	2.62879	-3.31616
H	-1.93355	1.58013	-2.58124
H	-0.20414	1.09608	-2.55132
C	-2.37474	-0.71812	-0.12256
C	-2.37792	0.67777	0.13478
C	-3.58821	1.34189	0.29473
H	-3.57812	2.38854	0.58578
C	-4.82162	0.67200	0.15692
C	-4.81455	-0.71932	-0.22776
C	-3.57604	-1.38487	-0.32311
H	-3.55286	-2.43644	-0.59925
C	-6.09251	-3.30969	-0.65601
H	-5.21542	-3.49629	-1.29515
C	-5.87720	-3.85118	0.75647
H	-5.73478	-4.94360	0.69842
H	-6.75374	-3.65713	1.39451
H	-4.99046	-3.41797	1.24569
C	-7.33899	-3.87948	-1.33954
H	-7.46038	-3.49629	-2.36429
H	-8.25365	-3.65966	-0.76507
H	-7.23402	-4.97496	-1.39662
C	-6.14530	3.25260	0.52130
H	-5.25975	3.46685	1.13931
C	-7.38822	3.81603	1.21612
H	-7.30309	4.91442	1.24379
H	-7.48188	3.45611	2.25211
H	-8.30999	3.56541	0.66608
C	-5.96422	3.76627	-0.90635
H	-5.82223	4.85981	-0.87416
H	-6.85508	3.55772	-1.51964
H	-5.08889	3.32287	-1.40639

Table S10. Final Atomic Coordinates for Optimized [(3,5-Cl_2-$Ph_2C_2S_2$)Pt(tptbz)]^0.

Energy: -3508023.6705175

Atom	Coordinates (Angstroms) X	Y	Z
C	-3.04368	-0.64907	-0.17558
C	-3.06585	0.64660	0.22622
S	-1.56878	1.51976	0.63007
Pt	0.12469	-0.01961	0.24993
S	-1.51888	-1.55342	-0.33345
S	1.91349	-1.58927	-0.10871
C	3.40440	-0.58720	0.08826
C	3.35993	0.76460	0.45246
S	1.81047	1.64328	0.72125
C	4.63537	-1.21748	-0.12123
C	5.84940	-0.54086	0.06114
C	5.80677	0.82352	0.44970
C	4.57076	1.44139	0.65100
C	-4.23513	-1.43015	-0.60089
C	-4.29593	1.44901	0.45685
S	7.29733	1.76409	0.78289
S	7.44839	-1.28513	-0.17315
C	1.68117	2.81363	-0.76726
C	2.52805	4.06529	-0.55617
C	1.93350	2.11485	-2.09576
C	2.05416	-2.69662	1.42566
C	1.93708	-1.91626	2.72682
C	1.01599	-3.80569	1.28266
C	7.10878	-3.05779	-0.61901
C	7.86966	2.24934	-0.93355
C	6.90391	3.21357	-1.61433
C	9.27180	2.83600	-0.76815
C	8.37572	-3.57343	-1.30877
C	6.73964	-3.90001	0.60320
C	-4.42687	2.72896	-0.10854
C	-5.57211	3.47831	0.14140
C	-6.60699	3.00463	0.94472
C	-6.45577	1.73662	1.50368
C	-5.32412	0.95952	1.27994
C	-4.48021	-2.70504	-0.06279
C	-5.58269	-3.43485	-0.49596
C	-6.46333	-2.94617	-1.45844
C	-6.19951	-1.68346	-1.98668
C	-5.10574	-0.92574	-1.58163
Cl	-5.88455	-5.02919	0.19620

Table S10, Continued. Final Atomic Coordinates for Optimized [(3,5-Cl_2-$Ph_2C_2S_2$)Pt(tptbz)]^0.

Energy: -3508023.6705175

Atom	Coordinates (Angstroms) X	Y	Z
Cl	-7.28207	-1.04004	-3.22048
Cl	-5.72803	5.07862	-0.58448
Cl	-7.73710	1.11151	2.54061
H	4.62837	-2.25945	-0.43056
H	4.56096	2.47783	0.98297
H	0.61291	3.06795	-0.68043
H	2.26894	4.80445	-1.33391
H	2.34139	4.52725	0.42582
H	3.60580	3.85484	-0.64996
H	1.74166	2.82876	-2.91568
H	1.26248	1.25175	-2.22500
H	2.97879	1.77425	-2.18460
H	3.06660	-3.11921	1.32236
H	2.08778	-2.60875	3.57397
H	2.69781	-1.12348	2.80151
H	0.94048	-1.45602	2.82092
H	1.12623	-4.50515	2.12888
H	1.14935	-4.37436	0.34855
H	-0.00857	-3.40052	1.30272
H	6.28919	-3.05999	-1.35425
H	7.93482	1.31455	-1.51065
H	7.26708	3.45013	-2.63091
H	5.89708	2.77782	-1.71415
H	6.81967	4.15831	-1.05113
H	9.66890	3.12582	-1.75602
H	9.96593	2.10807	-0.31954
H	9.25753	3.73927	-0.13402
H	8.23835	-4.63284	-1.58342
H	8.59988	-3.00549	-2.22494
H	9.25076	-3.51227	-0.63940
H	6.51934	-4.93696	0.29284
H	5.85607	-3.50756	1.13011
H	7.57385	-3.92632	1.32311
H	-3.64072	3.12783	-0.74572
H	-7.49600	3.60155	1.13165
H	-5.23215	-0.01758	1.74752
H	-3.81652	-3.11515	0.69513
H	-7.32057	-3.52792	-1.78753
H	-4.92067	0.04773	-2.02878

Table S11. Final Atomic Coordinates for Optimized $[(3,5\text{-}Cl_2\text{-}Ph_2C_2S_2)Pt(tptbz)]^{1+}$.

Energy: -3507888.0096219

Atom	Coordinates (Angstroms) X	Y	Z
C	-3.03176	0.66177	0.19630
C	-3.05334	-0.67811	-0.22857
S	-1.59598	-1.53128	-0.57937
Pt	0.12850	-0.00493	-0.18341
S	-1.55067	1.52902	0.36573
S	1.91016	1.56973	0.16571
C	3.40204	0.57396	-0.04692
C	3.35236	-0.77746	-0.40513
S	1.80534	-1.67129	-0.60267
C	4.63150	1.21271	0.12846
C	5.84466	0.53733	-0.08791
C	5.79556	-0.83240	-0.47098
C	4.55671	-1.45525	-0.63504
C	-4.24239	1.42050	0.59537
C	-4.29672	-1.44580	-0.48205
S	7.27105	-1.77228	-0.85924
S	7.43958	1.28416	0.08507
C	1.70337	-2.78683	0.93828
C	2.60069	-4.00831	0.76368
C	1.94091	-2.02531	2.23433
C	2.00971	2.68273	-1.37220
C	1.90181	1.90241	-2.67360
C	0.97198	3.79016	-1.22182
C	7.12394	3.06718	0.51918
C	7.97103	-2.18478	0.83279
C	7.05708	-3.11754	1.62001
C	9.35531	-2.78261	0.57982
C	8.42195	3.57990	1.15152
C	6.71426	3.89160	-0.70207
C	-4.44021	-2.74392	0.03698
C	-5.59498	-3.46969	-0.24525
C	-6.60935	-2.94563	-1.04576
C	-6.44519	-1.65949	-1.56323
C	-5.31032	-0.90190	-1.29029
C	-4.45996	2.71470	0.09288
C	-5.57773	3.43241	0.51201
C	-6.48192	2.90355	1.43235
C	-6.24430	1.62116	1.93022
C	-5.14464	0.87183	1.52334
Cl	-5.85567	5.03653	-0.13294

Table S11, Continued. Final Atomic Coordinates for Optimized [(3,5-Cl_2-$Ph_2C_2S_2$)Pt(tptbz)]$^{1+}$.

Energy: -3507888.0096219

Atom	Coordinates (Angstroms) X	Y	Z
Cl	-7.35756	0.94174	3.09815
Cl	-5.77882	-5.07834	0.42156
Cl	-7.69854	-0.98551	-2.58283
H	4.63412	2.25691	0.43133
H	4.54185	-2.49381	-0.96054
H	0.64729	-3.09063	0.86321
H	2.37243	-4.72577	1.57011
H	2.42924	-4.51579	-0.19809
H	3.66759	-3.74825	0.84728
H	1.79477	-2.71680	3.08169
H	1.23658	-1.18698	2.35059
H	2.97095	-1.63777	2.29365
H	3.02016	3.10742	-1.26368
H	2.07302	2.59475	-3.51606
H	2.65479	1.10247	-2.74090
H	0.89984	1.45827	-2.79001
H	1.12187	4.52057	-2.03445
H	1.07039	4.32480	-0.26402
H	-0.05432	3.40023	-1.30841
H	6.33409	3.08335	1.28664
H	8.08012	-1.22773	1.36395
H	7.49537	-3.31335	2.61487
H	6.06053	-2.67406	1.77851
H	6.93232	-4.08474	1.10482
H	9.82705	-3.03048	1.54560
H	10.01278	-2.07636	0.04937
H	9.29157	-3.71211	-0.01129
H	8.30179	4.64325	1.41729
H	8.67769	3.02308	2.06602
H	9.26873	3.50469	0.44865
H	6.51789	4.93511	-0.39941
H	5.80638	3.50072	-1.18866
H	7.51899	3.89885	-1.45458
H	-3.67025	-3.17594	0.67253
H	-7.50528	-3.52326	-1.26130
H	-5.20807	0.09135	-1.71994
H	-3.77786	3.14986	-0.63429
H	-7.34998	3.47435	1.75397
H	-4.98180	-0.11875	1.94039

Table S12. Final Atomic Coordinates for Optimized [(3,5-Cl_2-$Ph_2C_2S_2$)Pt(tptbz)]$^{2+}$, Closed Shell Singlet.

Energy: -3507668.6374759

Atom	Coordinates (Angstroms) X	Y	Z
C	-3.04116	0.69015	0.20635
C	-3.07458	-0.71150	-0.20404
S	-1.63121	-1.56845	-0.45924
Pt	0.12011	-0.03723	-0.16006
S	-1.56718	1.52591	0.30497
S	1.89339	1.55966	0.00443
C	3.39242	0.56055	-0.09218
C	3.34452	-0.82062	-0.31644
S	1.79441	-1.71906	-0.42279
C	4.61617	1.21789	0.01998
C	5.83344	0.52512	-0.13495
C	5.78596	-0.88150	-0.37877
C	4.54588	-1.51915	-0.48116
C	-4.23630	1.42411	0.62560
C	-4.31709	-1.42630	-0.49654
S	7.24065	-1.86757	-0.70185
S	7.41574	1.29295	-0.07834
C	1.69277	-2.72227	1.20935
C	2.60966	-3.93782	1.12389
C	1.91867	-1.85328	2.43735
C	1.96889	2.51123	-1.64980
C	1.93153	1.59039	-2.85903
C	0.88151	3.57915	-1.63216
C	7.11654	3.10436	0.25361
C	8.09524	-2.02144	0.97012
C	7.23215	-2.78388	1.96914
C	9.43056	-2.70721	0.68037
C	8.42775	3.64161	0.83672
C	6.69664	3.85099	-1.01348
C	-4.44558	-2.79193	-0.15690
C	-5.59564	-3.48249	-0.51834
C	-6.61912	-2.85474	-1.24391
C	-6.47825	-1.50863	-1.59486
C	-5.35336	-0.78207	-1.21741
C	-4.38223	2.78964	0.29425
C	-5.47853	3.49709	0.77208
C	-6.42752	2.88637	1.60500
C	-6.26749	1.53996	1.94606
C	-5.19823	0.79689	1.45558

Table S12, Continued. Final Atomic Coordinates for Optimized [(3,5-Cl_2-$Ph_2C_2S_2$)Pt(tptbz)]$^{2+}$, Closed Shell Singlet.

Energy: -3507668.6374759

Atom	Coordinates (Angstroms) X	Y	Z
Cl	-5.68776	5.16407	0.33000
Cl	-7.42851	0.77972	2.99509
Cl	-5.77986	-5.14861	-0.06322
Cl	-7.73339	-0.72834	-2.51218
H	4.62102	2.28731	0.21690
H	4.53310	-2.58543	-0.70051
H	0.64199	-3.04661	1.14888
H	2.37810	-4.59791	1.97697
H	2.45500	-4.51610	0.20023
H	3.67130	-3.65827	1.20489
H	1.79561	-2.48156	3.33593
H	1.19515	-1.02516	2.49804
H	2.93965	-1.43991	2.45920
H	2.95755	2.98813	-1.55827
H	2.08255	2.20040	-3.76619
H	2.73033	0.83417	-2.83247
H	0.95679	1.08266	-2.94731
H	1.03931	4.24424	-2.49746
H	0.91691	4.19946	-0.72291
H	-0.12351	3.13898	-1.73332
H	6.33615	3.16461	1.02858
H	8.27809	-0.99850	1.32719
H	7.76419	-2.84902	2.93450
H	6.27131	-2.27573	2.15421
H	7.02897	-3.81087	1.62275
H	9.98924	-2.81524	1.62490
H	10.04957	-2.11989	-0.01530
H	9.28614	-3.71575	0.25783
H	8.31480	4.71895	1.04079
H	8.69258	3.13928	1.77944
H	9.26363	3.52231	0.12744
H	6.52051	4.91360	-0.77289
H	5.77521	3.44406	-1.46093
H	7.48854	3.80156	-1.77753
H	-3.67420	-3.29651	0.42087
H	-7.51286	-3.40846	-1.52617
H	-5.25911	0.25318	-1.53221
H	-3.66994	3.28153	-0.36459
H	-7.27922	3.45304	1.97712
H	-5.08484	-0.23852	1.76367

Table S13. Final Atomic Coordinates for Optimized $[(3,5\text{-}Cl_2\text{-}Ph_2C_2S_2)Pt(tptbz)]^{2+}$, Singlet Diradical.

Energy: -3507669.1106306

Atom	Coordinates (Angstroms) X	Y	Z
C	-3.04462	0.68650	0.20159
C	-3.07840	-0.70655	-0.20376
S	-1.63276	-1.56330	-0.48023
Pt	0.11544	-0.03499	-0.17279
S	-1.56649	1.52443	0.30893
S	1.89361	1.55999	0.01335
C	3.39100	0.55896	-0.10008
C	3.34233	-0.82163	-0.34085
S	1.79006	-1.71776	-0.46317
C	4.61585	1.21163	0.01865
C	5.83252	0.51909	-0.14463
C	5.78413	-0.88689	-0.39479
C	4.54127	-1.51990	-0.51211
C	-4.23885	1.43025	0.61362
C	-4.32065	-1.43296	-0.47931
S	7.23056	-1.88332	-0.70863
S	7.41172	1.29201	-0.11703
C	1.68901	-2.74821	1.15118
C	2.60097	-3.96585	1.04795
C	1.91319	-1.90108	2.39467
C	1.97279	2.53644	-1.62688
C	1.91838	1.63448	-2.84963
C	0.89422	3.61273	-1.58585
C	7.12978	3.08013	0.34173
C	8.12536	-1.99973	0.94808
C	7.28415	-2.75629	1.97031
C	9.45966	-2.67847	0.63899
C	8.44588	3.56573	0.95792
C	6.71402	3.91208	-0.87286
C	-4.44239	-2.79107	-0.11166
C	-5.59323	-3.49219	-0.45139
C	-6.62420	-2.88294	-1.18009
C	-6.48906	-1.54350	-1.55773
C	-5.36282	-0.80682	-1.20509
C	-4.38602	2.78791	0.25505
C	-5.48252	3.50425	0.72030
C	-6.43072	2.91061	1.56471
C	-6.26858	1.57125	1.93143
C	-5.19824	0.81972	1.45675

Table S13, Continued. Final Atomic Coordinates for Optimized $[(3,5\text{-}Cl_2\text{-}Ph_2C_2S_2)Pt(tptbz)]^{2+}$, Singlet Diradical.

Energy: -3507669.1106306

Atom	Coordinates (Angstroms) X	Y	Z
Cl	-5.69092	5.16381	0.24566
Cl	-7.42947	0.83023	2.99581
Cl	-5.76664	-5.15144	0.03732
Cl	-7.75458	-0.78407	-2.48004
H	4.62311	2.27955	0.22322
H	4.52518	-2.58418	-0.74067
H	0.63716	-3.06738	1.08292
H	2.36384	-4.63997	1.88842
H	2.44722	-4.52721	0.11378
H	3.66370	-3.69277	1.13806
H	1.78102	-2.54342	3.28195
H	1.19402	-1.07008	2.46475
H	2.93659	-1.49428	2.42969
H	2.96583	3.00399	-1.53496
H	2.06556	2.25715	-3.74882
H	2.71182	0.87203	-2.84206
H	0.93954	1.13478	-2.93660
H	1.04896	4.28633	-2.44513
H	0.94376	4.22176	-0.66972
H	-0.11517	3.18185	-1.68114
H	6.34919	3.08905	1.11832
H	8.30387	-0.96994	1.28504
H	7.83592	-2.80586	2.92544
H	6.32384	-2.25212	2.16726
H	7.08227	-3.78895	1.64092
H	10.03594	-2.77144	1.57445
H	10.06139	-2.09424	-0.07413
H	9.31558	-3.69307	0.23124
H	8.34040	4.62785	1.23331
H	8.70709	3.00021	1.86511
H	9.28052	3.48861	0.24142
H	6.55114	4.95780	-0.56030
H	5.78627	3.54623	-1.34167
H	7.50274	3.90577	-1.64173
H	-3.66475	-3.28193	0.46947
H	-7.51842	-3.44432	-1.44466
H	-5.27398	0.22302	-1.53900
H	-3.67418	3.26708	-0.41355
H	-7.28258	3.48362	1.92624
H	-5.08401	-0.21002	1.78333

Table S14. Final Atomic Coordinates for Optimized $[(3,5\text{-}Cl_2\text{-}Ph_2C_2S_2)Pt(tptbz)]^{2+}$, Triplet.

Energy: -3507663.4396581

Atom	Coordinates (Angstroms) X	Y	Z
C	-3.04947	0.66648	0.19536
C	-3.08112	-0.67942	-0.21201
S	-1.62825	-1.53949	-0.56955
Pt	0.10613	-0.01697	-0.21348
S	-1.55954	1.52433	0.34432
S	1.90025	1.56440	0.07872
C	3.39168	0.56232	-0.10518
C	3.34107	-0.80813	-0.43841
S	1.78364	-1.68949	-0.63570
C	4.61884	1.19717	0.05494
C	5.83143	0.50846	-0.15500
C	5.78148	-0.88555	-0.46861
C	4.53287	-1.50083	-0.65023
C	-4.25277	1.43725	0.58733
C	-4.32944	-1.44508	-0.43704
S	7.22173	-1.87603	-0.76759
S	7.39937	1.29194	-0.19779
C	1.68204	-2.83985	0.89308
C	2.57867	-4.05815	0.70664
C	1.89800	-2.09349	2.20090
C	1.99756	2.64762	-1.49433
C	1.89047	1.83210	-2.77296
C	0.94841	3.74536	-1.35903
C	7.21477	2.93565	0.68540
C	8.15376	-1.96030	0.88167
C	7.52540	-3.04919	1.74638
C	9.61930	-2.21578	0.53831
C	8.55064	3.19493	1.38866
C	6.84406	4.04200	-0.30462
C	-4.45545	-2.75383	0.05150
C	-5.62551	-3.47705	-0.20602
C	-6.67340	-2.93199	-0.95237
C	-6.53143	-1.63413	-1.44225
C	-5.37873	-0.88265	-1.19220
C	-4.44335	2.74343	0.11349
C	-5.56426	3.47059	0.52820
C	-6.49863	2.93200	1.41722
C	-6.29212	1.63661	1.88982
C	-5.18736	0.88124	1.48512
Cl	-5.80161	5.08096	-0.08221

Table S14, Continued. Final Atomic Coordinates for Optimized [(3,5-Cl_2-$Ph_2C_2S_2$)Pt(tptbz)]$^{2+}$, Triplet.

Energy: -3507663.4396581

Atom	X	Y	Z
Cl	-7.42740	0.94062	3.01148
Cl	-5.77791	-5.09038	0.42307
Cl	-7.80994	-0.93006	-2.39041
H	4.63471	2.25399	0.31203
H	4.50767	-2.54964	-0.94098
H	0.62661	-3.13812	0.79214
H	2.32409	-4.79319	1.48891
H	2.42912	-4.54276	-0.27021
H	3.64467	-3.80948	0.83029
H	1.73586	-2.79777	3.03464
H	1.19201	-1.25704	2.31879
H	2.92823	-1.71078	2.28680
H	3.00438	3.08277	-1.39323
H	2.02970	2.51016	-3.63258
H	2.66422	1.05160	-2.83837
H	0.89874	1.36049	-2.86527
H	1.08973	4.45858	-2.18824
H	1.04592	4.30281	-0.41446
H	-0.07370	3.34283	-1.43584
H	6.42580	2.78962	1.43827
H	8.03799	-0.97493	1.35085
H	8.06986	-3.09398	2.70636
H	6.46711	-2.83963	1.96951
H	7.59923	-4.04045	1.27063
H	10.18953	-2.31247	1.47713
H	10.05467	-1.39059	-0.04535
H	9.74825	-3.15439	-0.02621
H	8.49036	4.16533	1.90844
H	8.78077	2.42095	2.13630
H	9.38419	3.25404	0.66938
H	6.75067	4.99417	0.24476
H	5.89004	3.84929	-0.82022
H	7.62518	4.16817	-1.07105
H	-3.66655	-3.20509	0.64930
H	-7.57643	-3.50660	-1.14683
H	-5.29070	0.11534	-1.61361
H	-3.74544	3.18903	-0.59199
H	-7.36455	3.51010	1.73253
H	-5.04319	-0.11526	1.89452

Table S15. Final Atomic Coordinates for Optimized $[((NC)_2C_2S_2)Pd(tptbz)]^0$.

Atom	Coordinates (Angstroms) X	Y	Z
Pd	2.209330	0.003583	0.086298
S	3.861897	-1.414004	-0.722196
S	3.870129	1.529051	0.629034
S	0.416717	-1.572265	-0.397198
S	0.485013	1.589546	0.742535
S	-5.003217	1.514243	1.065175
S	-5.117579	-1.386416	-0.211608
N	7.498819	-1.726961	-1.248561
N	7.499604	2.160460	0.421488
C	6.547376	-1.156186	-0.892606
C	5.354148	-0.500967	-0.472033
C	5.356549	0.748826	0.076822
C	6.548821	1.506855	0.258331
C	-1.077858	-0.629388	-0.023449
C	-2.304497	-1.270266	-0.225009
H	-2.291733	-2.276712	-0.635277
C	-3.524606	-0.641930	0.064722
C	-3.491394	0.675874	0.587885
C	-2.260915	1.304437	0.789029
H	-2.248026	2.295158	1.240660
C	-1.047022	0.688790	0.456157
C	0.393563	-2.840626	1.007614
H	-0.626358	-3.252248	0.944311
C	1.408685	-3.927627	0.664072
H	2.437283	-3.532652	0.657748
H	1.202253	-4.386867	-0.315670
H	1.352229	-4.717954	1.431788
C	0.619956	-2.204299	2.372019
H	-0.127967	-1.426548	2.592337
H	1.624986	-1.756526	2.437084
H	0.534786	-2.983267	3.150200
C	0.561173	2.847049	-0.671164
H	1.602061	3.183444	-0.540199
C	-0.389782	4.014217	-0.422312
H	-0.178824	4.808207	-1.159299
H	-0.261544	4.442401	0.583978
H	-1.443459	3.716826	-0.551478
C	0.397655	2.207413	-2.043485
H	0.534859	2.981964	-2.817875
H	-0.609508	1.777053	-2.173247
H	1.148170	1.419368	-2.211193

Table S15, Continued. Final Atomic Coordinates for Optimized $[((NC)_2C_2S_2)Pd(tptbz)]^0$.

Atom	Coordinates (Angstroms) X	Y	Z
C	-5.039155	2.963914	-0.114821
H	-4.082890	3.495382	0.018458
C	-6.179827	3.871780	0.346399
H	-7.152889	3.356069	0.280009
H	-6.226237	4.762424	-0.303032
H	-6.037323	4.211265	1.384607
C	-5.186666	2.522000	-1.567403
H	-5.166052	3.406199	-2.229912
H	-6.140419	1.992689	-1.725168
H	-4.368745	1.851098	-1.873939
C	-4.754647	-3.123516	-0.765886
H	-3.960017	-3.068607	-1.526406
C	-4.330164	-4.025693	0.393789
H	-3.436259	-3.646638	0.913333
H	-5.139273	-4.108631	1.137616
H	-4.102285	-5.039222	0.018307
C	-6.033466	-3.625535	-1.444080
H	-6.299785	-3.009368	-2.316810
H	-5.883659	-4.663329	-1.786256
H	-6.886464	-3.623482	-0.744272

Table S16. Final Atomic Coordinates for Optimized $[((NC)_2C_2S_2)Pd(tptbz)]^{1+}$.

Atom	Coordinates (Angstroms) X	Y	Z
Pd	2.154950	0.001274	-0.045378
S	3.898032	1.496039	0.490284
S	3.813963	-1.601721	-0.521707
S	0.435120	1.621921	0.400739
S	0.413721	-1.603287	-0.445161
S	-5.126696	-1.352204	-0.593184
S	-5.090730	1.504331	0.504653
N	7.559793	1.746363	0.754318
N	7.455598	-2.140122	-0.512941
C	6.555848	1.201266	0.527842
C	5.312811	0.559541	0.257263
C	5.277458	-0.781438	-0.177259
C	6.483114	-1.517899	-0.359414
C	-1.085121	0.678495	0.196030
C	-2.292793	1.351141	0.409394
H	-2.259887	2.382389	0.753245
C	-3.527291	0.723848	0.209263
C	-3.543626	-0.634829	-0.235436
C	-2.326824	-1.302012	-0.414786
H	-2.322393	-2.328220	-0.772893
C	-1.099576	-0.666480	-0.184201
C	0.367545	2.766508	-1.115546
H	-0.641973	3.194525	-1.010968
C	1.409316	3.866000	-0.936097
H	2.436284	3.479774	-1.034361
H	1.311696	4.377841	0.034016
H	1.262430	4.616729	-1.730405
C	0.480612	2.005825	-2.428495
H	-0.284722	1.219784	-2.517796
H	1.478403	1.549814	-2.542474
H	0.332791	2.712736	-3.263001
C	0.571376	-2.730180	1.083075
H	1.617936	-3.052390	0.963063
C	-0.347715	-3.938855	0.936957
H	-0.107037	-4.657073	1.739025
H	-0.207365	-4.450681	-0.027494
H	-1.407514	-3.662714	1.048322
C	0.399023	-1.972572	2.391837
H	0.561811	-2.672450	3.228963
H	-0.618272	-1.561361	2.492101
H	1.126911	-1.151564	2.491138

Table S16, Continued. Final Atomic Coordinates for Optimized $[((NC)_2C_2S_2)Pd(tptbz)]^{1+}$.

Atom	Coordinates (Angstroms)		
	X	Y	Z
C	-4.847978	-3.189753	-0.692076
H	-3.990141	-3.357129	-1.362226
C	-6.099744	-3.764973	-1.362012
H	-7.005069	-3.557940	-0.767319
H	-5.994914	-4.859455	-1.443412
H	-6.245367	-3.356814	-2.373963
C	-4.590698	-3.799810	0.684732
H	-4.384671	-4.879637	0.581941
H	-5.471293	-3.678814	1.335788
H	-3.731935	-3.333627	1.193121
C	-4.720490	3.310029	0.764385
H	-3.905680	3.372143	1.503157
C	-4.332958	4.013130	-0.536068
H	-3.448394	3.560009	-1.011760
H	-5.160690	3.974791	-1.262106
H	-4.105220	5.073864	-0.330974
C	-5.985192	3.905087	1.391993
H	-6.227561	3.426617	2.353290
H	-5.826706	4.981010	1.572763
H	-6.854000	3.801879	0.720664

Table S17. Final Atomic Coordinates for Optimized $[((NC)_2C_2S_2)Pt(tptbz)]^0$.

Atom	Coordinates (Angstroms) X	Y	Z
Pt	1.975545	-0.012236	0.062468
S	3.608343	1.564577	0.575264
S	3.654947	-1.462550	-0.643924
S	0.244191	1.562999	0.625959
S	0.244065	-1.615389	-0.392776
S	-5.288919	1.390215	0.759984
S	-5.295022	-1.473708	-0.458086
N	7.236140	2.191705	0.427938
N	7.301825	-1.744154	-1.070510
C	5.104877	0.760347	0.093812
C	5.124390	-0.514478	-0.398821
C	6.290560	1.529259	0.270712
C	6.334824	-1.171803	-0.762471
C	-1.277483	0.647908	0.322031
C	-1.274776	-0.679785	-0.120895
C	-2.488910	-1.333799	-0.354665
H	-2.457384	-2.348736	-0.743565
C	-3.720083	-0.708361	-0.127270
C	-3.722721	0.623734	0.367750
C	-2.503696	1.268478	0.591334
H	-2.498612	2.271647	1.011603
C	0.331311	2.806868	-0.806314
H	1.390951	3.092212	-0.712818
C	-0.553144	4.018300	-0.526576
H	-0.327181	4.801389	-1.270672
H	-0.369473	4.439398	0.474240
H	-1.624026	3.775502	-0.620948
C	0.089465	2.166926	-2.165863
H	-0.944410	1.795870	-2.263577
H	0.787792	1.335261	-2.345711
H	0.250114	2.927377	-2.949650
C	0.164052	-2.823420	1.068814
H	-0.836678	-3.268086	0.945913
C	1.231732	-3.890007	0.844197
H	2.245958	-3.462664	0.894758
H	1.111157	-4.393688	-0.128130
H	1.141883	-4.651426	1.637559
C	0.270109	-2.126014	2.417013
H	0.142694	-2.875965	3.217632
H	-0.510205	-1.359885	2.546916
H	1.255695	-1.648650	2.537665

Table S17, Continued. Final Atomic Coordinates for Optimized $[((NC)_2C_2S_2)Pt(tptbz)]^0$.

Atom	X	Y	Z
C	-5.047124	3.188432	0.339157
H	-4.188845	3.553067	0.924739
C	-4.796781	3.401914	-1.151469
H	-5.663819	3.071380	-1.746417
H	-3.911968	2.848182	-1.502407
H	-4.627567	4.475062	-1.351511
C	-6.304952	3.908017	0.832433
H	-6.211099	4.987929	0.628747
H	-6.452444	3.775117	1.915847
H	-7.205863	3.543088	0.310990
C	-4.915582	-3.267028	-0.764418
H	-4.098275	-3.310181	-1.501310
C	-4.525782	-4.005774	0.515798
H	-5.356125	-3.993259	1.240421
H	-3.646521	-3.557846	1.004518
H	-4.286946	-5.058803	0.282967
C	-6.173218	-3.856862	-1.410494
H	-6.009494	-4.927758	-1.617201
H	-6.416161	-3.355722	-2.360355
H	-7.045515	-3.773689	-0.740234

Coordinates (Angstroms)

Table S18. Final Atomic Coordinates for Optimized $[((NC)_2C_2S_2)Pt(tptbz)]^{1+}$.

Atom	Coordinates (Angstroms) X	Y	Z
Pt	-1.959049	0.003403	0.040884
S	-3.617222	-1.600285	0.486042
S	-3.686692	1.502727	-0.494622
S	-0.224929	-1.596530	0.443281
S	-0.250214	1.628377	-0.366497
S	5.317919	-1.343786	0.572377
S	5.274560	1.509224	-0.530395
N	-7.257526	-2.136553	0.451322
N	-7.342844	1.768944	-0.796771
C	-5.078846	-0.774815	0.138171
C	-5.107855	0.567532	-0.284921
C	-6.284484	-1.513045	0.308013
C	-6.344066	1.217458	-0.563806
C	1.289203	-0.659743	0.188569
C	1.272313	0.684300	-0.191484
C	2.477357	1.357889	-0.413992
H	2.441375	2.389584	-0.756189
C	3.713183	0.730495	-0.222354
C	3.732411	-0.627497	0.226181
C	2.516969	-1.295132	0.413779
H	2.515147	-2.321595	0.770968
C	-0.359298	-2.738120	-1.083063
H	-1.421404	-3.018222	-1.005190
C	0.508202	-3.975166	-0.875857
H	0.278184	-4.697578	-1.677200
H	0.306050	-4.465883	0.088622
H	1.581529	-3.739759	-0.943085
C	-0.100984	-2.003894	-2.390246
H	0.936188	-1.637651	-2.453150
H	-0.789232	-1.155341	-2.527680
H	-0.258866	-2.708716	-3.224233
C	-0.165842	2.748770	1.172348
H	0.821233	3.215952	1.027481
C	-1.255563	3.808650	1.053803
H	-2.260353	3.381220	1.198097
H	-1.224676	4.333022	0.085802
H	-1.098048	4.557730	1.847646
C	-0.192821	1.965023	2.475331
H	-0.029104	2.666424	3.311552
H	0.601609	1.204509	2.517837
H	-1.167950	1.473504	2.626716

Table S18, Continued. Final Atomic Coordinates for Optimized $[((NC)_2C_2S_2)Pt(tptbz)]^{1+}$.

Atom	Coordinates (Angstroms)		
	X	Y	Z
C	5.036452	-3.179095	0.701376
H	4.185418	-3.334161	1.383091
C	4.763535	-3.809179	-0.663431
H	5.637755	-3.700491	-1.325145
H	3.900921	-3.348621	-1.170473
H	4.555975	-4.886782	-0.542098
C	6.294010	-3.747046	1.366688
H	6.187813	-4.839977	1.465235
H	6.450891	-3.324395	2.370961
H	7.193511	-3.550737	0.759662
C	4.902651	3.312450	-0.805570
H	4.081694	3.366763	-1.538108
C	4.526253	4.028968	0.490842
H	5.360033	3.997777	1.210252
H	3.645538	3.581318	0.978691
H	4.297218	5.087609	0.276686
C	6.162201	3.900526	-1.449900
H	6.002492	4.974636	-1.640088
H	6.396158	3.412318	-2.408363
H	7.036663	3.803777	-0.784981

References

(**1**) (a) *APEX2*, Bruker-AXS, Inc., Madison, Wisconsin, USA, 2015. (b) *APEX3*, Bruker-AXS, Inc., Madison, Wisconsin, USA, 2020. (c) *APEX4*, Bruker-AXS, Inc., Madison, Wisconsin, USA, 2021.

(**2**) (a) *SAINT*, Bruker AXS, Inc., Madison, Wisconsin, 2015. (b) *SAINT*, Bruker AXS, Inc., Madison, Wisconsin, 2020. (c) *SAINT*, Bruker AXS, Inc., Madison, Wisconsin, 2021.

(**3**) Sheldrick, G, M. *CELL_NOW*, University of Göttingen, Göttingen, Germany, 2008.

(**4**) Sheldrick, G, M. *TWINABS*, University of Göttingen, Göttingen, Germany, 2009.

(**5**) *SADABS*, Bruker AXS, Inc., Madison, Wisconsin, 2016.

(**6**) Krause, L.; Herbst-Irmer, R.; Sheldrick, G.M.; Stalke; D. Comparison of Silver and Molybdenum Microfocus X-ray Sources for Single-Crystal Structure Determination. *J. Appl. Cryst.* **2015**, *48*, 3-10.

(**7**) (**a**) Sheldrick, G. M. *SHELXS-97*, University of Göttingen, Göttingen, Germany, **2008**. (**b**) Sheldrick, G. M. A Short History of SHELX. *Acta Crystallogr., Sect. A: Foundations Adv.* **2008**, *64*, 112-122.

(**8**) Sheldrick, G. M. *SHELXT* – Integrated Space-Group and Crystal-Structure Determination. *Acta Crystallogr., Sect A* **2015**, *71*, 3-8.

(**9**) (**a**) Sheldrick, G. M. A Short History of *SHELX*. *Acta Crystallogr., Sect. A* **2008**, *64*, 112-122. (**b**) Sheldrick, G. M. *SHELXL-2018/1*. University of Göttingen, Göttingen, Germany, 2018.

(**10**) (a) *SHELXTL*, Bruker-AXS, Madison, WI, 2015. (b) *SHELXTL*, Bruker-AXS, Madison, WI, 2020. (c) *SHELXTL*, Bruker-AXS, Madison, WI, 2021.

(**11**) Parsons, S.; Flack, H. D.; Wagner, T. Use of Intensity Quotients and Differences in Absolute Structure Refinement. *Acta Crystallogr., Sect. B* **2013**, *69*, 249-259.

(**12**) See http://checkcif.iucr.org/

(**13**) *Gaussian 09*, Revision D.01. Frisch, M. J.; Trucks, G. W.; Schlegel, H. B.; Scuseria, G. E.; Robb, M. A.; Cheeseman, J. R.; Scalmani, G.; Barone, V.; Petersson, G. A.; Nakatsuji, H.; Li, X.; Caricato, M.; Marenich, A.; Bloino, J.; Janesko, B. J.; Gomperts, R.; Mennucci, B.; Hratchian, H. P.; Ortiz, J. V.; Izmaylov, A. F.; Sonnenberg, J. L.; Williams-Young, D.; Ding, F.; Lipparini, F.; Egidi, F.; Goings, J.; Peng, B.; Petrone, A.; Henderson, T.; Ranasinghe, D.; Zakrzewski, V. G.; Gao, J.; Rega, N.; Zheng, G.; Liang, W.; Hada, M.; Ehara, M.; Toyota, K.; Fukuda, R.; Hasegawa, J.; Ishida, M.; Nakajima, T.; Honda, Y.; Kitao, O.; Nakai, H.; Vreven, T.; Throssell, K.; Montgomery, Jr., J. A.; Peralta, J. E.; Ogliaro, F.; Bearpark, M.; Heyd, J. J.; Brothers, E.; Kudin, K. N.; Staroverov, V. N.; Keith, T.; Kobayashi, R.; Normand, J.; Raghavachari, K.; Rendell, A.; Burant, J. C.; Iyengar, S. S.; Tomasi, J.; Cossi, M.; Millam, J. M.; Klene, M.; Adamo, C.; Cammi, R.; Ochterski, J. W.; Martin, R. L.; Morokuma, K.; Farkas, O.; Foresman, J. B.; Fox, D. J. Gaussian, Inc., Wallingford, CT, 2016.

(**14**) Becke, A. D. Density-Functional Thermochemistry. III. The Role of Exact Exchange. *J. Chem. Phys.* **1993**, *98*(7), 5648-5652.

(**15**) Lee, C.; Yang, W.; Parr, R. G. Development of the Colle-Salvetti Correlation-Energy Formula into a Functional of the Electron Density. *Phys. Rev. B* **1988**, *37*(2), 785-789.

(**16**) https://www.basissetexchange.org/ (accessed 21 June 2022).

(**17**) Schäfer, A.; Horn, H.; Ahlrichs, R. Fully Optimized Contracted Gaussian Basis Sets for Atoms Li to Kr. *J. Chem. Phys.* **1992**, *94*(4), 2571-2577.

(**18**) *Jmol: an open-source Java viewer for chemical structures in 3D*. http://www.jmol.org.
